# Capturing excited-state structural snapshots of evolutionary green-to-red photochromic fluorescent proteins

**DOI:** 10.3389/fchem.2023.1328081

**Published:** 2023-12-07

**Authors:** Taylor D. Krueger, J. Nathan Henderson, Isabella L. Breen, Liangdong Zhu, Rebekka M. Wachter, Jeremy H. Mills, Chong Fang

**Affiliations:** ^1^ Department of Chemistry, Oregon State University, Corvallis, OR, United States; ^2^ Center for Molecular Design and Biomimetics, The Biodesign Institute, Arizona State University, Tempe, AZ, United States; ^3^ School of Molecular Sciences, Arizona State University, Tempe, AZ, United States

**Keywords:** photoconvertible fluorescent proteins, photoswitchable fluorescent proteins, ultrafast spectroscopy, X-ray crystallography, femtosecond stimulated Raman, structural dynamics, biophysical chemistry and biomolecules

## Abstract

Photochromic fluorescent proteins (FPs) have proved to be indispensable luminous probes for sophisticated and advanced bioimaging techniques. Among them, an interplay between photoswitching and photoconversion has only been observed in a limited subset of Kaede-like FPs that show potential for discovering the key mechanistic steps during green-to-red photoconversion. Various spectroscopic techniques including femtosecond stimulated Raman spectroscopy (FSRS), X-ray crystallography, and femtosecond transient absorption were employed on a set of five related FPs with varying photoconversion and photoswitching efficiencies. A 3-methyl-histidine chromophore derivative, incorporated through amber suppression using orthogonal aminoacyl tRNA synthetase/tRNA pairs, displays more dynamic photoswitching but greatly reduced photoconversion *versus* the least-evolved ancestor (LEA). Excitation-dependent measurements of the green anionic chromophore reveal that the varying photoswitching efficiencies arise from both the initial transient dynamics of the bright *cis* state and the final *trans*-like photoswitched *off* state, with an exocyclic bridge H-rocking motion playing an active role during the excited-state energy dissipation. This investigation establishes a close-knit feedback loop between spectroscopic characterization and protein engineering, which may be especially beneficial to develop more versatile FPs with targeted mutations and enhanced functionalities, such as photoconvertible FPs that also feature photoswitching properties.

## 1 Introduction

Photoconvertible and photoswitchable fluorescent proteins (FPs) are genetically encodable phototransformable biomarkers that can irreversibly and reversibly alter their electronic absorption and emission spectral features, respectively, upon light exposure of specific wavelengths (Patterson and Lippincott-Schwartz, 2002; Fernández-Suárez and Ting, 2008; Chudakov et al., 2010; Jung, 2012; Zhou and Lin, 2013; Adam et al., 2014; Milo and Phillips, 2015; Acharya et al., 2017). A photoconvertible FP (pcFP) typically redshifts the steady-state electronic features due to an extension of the chromophore conjugation upon light activation, while a reversibly photoswitchable FP (rsFP) can be toggled back and forth between dark and bright fluorescent states upon light illumination of two distinct wavelengths (Habuchi et al., 2005; Andresen et al., 2007; Henderson et al., 2009; Meech, 2009; Bourgeois and Adam, 2012; Deo, 2012; Dedecker et al., 2013). These unique photochromic biomolecules have been instrumental toward the development of modern imaging techniques with technical innovations such as super-resolution imaging, where the exquisite external control afforded by these luminous bioprobes allows for sophisticated techniques capable of achieving resolution below the light diffraction limit (Betzig et al., 2006; Chudakov et al., 2007; Moerner, 2007; Fernández-Suárez and Ting, 2008; Blum and Subramaniam, 2009; Huang et al., 2010; Nienhaus and Nienhaus, 2014; Shcherbakova et al., 2014). While it is rare to have an FP with both photoconvertible and photoswitchable properties, such FPs are desirable for imaging measurements because they give an extra element of external control to manipulate the emitting populations, which can lead to even more sophisticated imaging techniques including the dynamic tracking of cellular processes and pulse-chase localization-based imaging advances (Ando et al., 2007; Moerner, 2007; Adam et al., 2008; Andresen et al., 2008; Shroff et al., 2008; Subach et al., 2009; Subach et al., 2010; Gunewardene et al., 2011; Nienhaus and Nienhaus, 2014; Shcherbakova et al., 2014).

Among the broad array of phototransformable FPs that span the visible spectrum, green-to-red pcFPs are renowned for imaging techniques that rely on stochastic photoactivation with relatively low illumination powers due to their superior brightness, photostability, photoconversion efficiency, and signal contrast, also coupled with their efficient protein maturation rates and effective separation between the photoconverting, imaging, and emitting wavelengths (Ando et al., 2002; Mizuno et al., 2003; Rust et al., 2006; Shaner et al., 2008; Yampolsky et al., 2008; Wachter et al., 2010; Shcherbakova et al., 2012; Subach and Verkhusha, 2012; Gorbachev et al., 2020). Green-to-red pcFPs can enable multicolor imaging measurements where multiple FPs with different properties are tagged to specific targets and imaged simultaneously yet individually, which have been extensively studied for Dendra2 (Adam et al., 2009; Fron et al., 2013; Makarov et al., 2014), EosFP (Ivanchenko et al., 2005; Nienhaus et al., 2005; De Zitter et al., 2020; Fare et al., 2020; Nienhaus and Nienhaus, 2021), Kaede (Hosoi et al., 2006; Li et al., 2010; Fron et al., 2014), and other Kaede-like FPs (Adam et al., 2008; Habuchi et al., 2008; Tsutsui et al., 2009; Kim et al., 2013; Colletier et al., 2016). Among them, there are a select few that display both photoconvertible and photoswitchable properties including IrisFP (Adam et al., 2008; Colletier et al., 2016) and mEos4b (Ivanchenko et al., 2005; Nienhaus et al., 2005; Berardozzi et al., 2016; De Zitter et al., 2020; Fare et al., 2020; Nienhaus and Nienhaus, 2021). IrisFP, named after the Greek goddess personifying the rainbow due to its unique photochromism, is a tetrameric variant of EosFP that undergoes green-to-red photoconversion besides photoswitching of the green and red chromophores, representing three distinct photoinduced processes. A systematic comparison between IrisFP and mEos4b found that the more dynamic photoswitching of the latter FP was correlated to a reduced number of hydrogen (H)-bond partners and a less stable chromophore in the photoswitched *off* state (Berardozzi et al., 2016; De Zitter et al., 2020). Another green-to-red pcFP named the least-evolved ancestor (LEA) (Kim et al., 2013; Kim et al., 2015) was also discovered to display pronounced photoswitching of both the green and red forms (Krueger et al., 2020; Krueger et al., 2023b). In fact, the prominent photoswitching of the green chromophore was exploited to enhance the photoconversion rate, red:green contrast, and red yield of LEA using a novel dual-illumination strategy with 400 and 505 nm light, compared to traditional photoconversion solely with 400 nm light (Krueger et al., 2020).

Several photoconversion mechanisms have been proposed for the Kaede-like pcFPs, and most mechanisms involve a β-elimination reaction and backbone breakage to extend the conjugation between Gly64 and His62 that forms the red chromophore, although the complete photocycle remains largely unanswered (Mizuno et al., 2003; Wachter et al., 2010; Shcherbakova et al., 2012; Subach and Verkhusha, 2012; Kim et al., 2015; Krueger et al., 2023a; Krueger et al., 2023b). In most Kaede-like FPs, the crystal structure, protein chromophore, and local environment display remarkable similarities before and after the photoconversion, which implies that fleeting transient interactions between the chromophore and surrounding residues can induce the necessary conformational changes during photoconversion (Wachter et al., 2010; Kim et al., 2015). The complex photophysical/chemical behavior warrants further investigations to unravel the highly intertwined photoresponse of these fascinating biomolecules. In particular, this current investigation will evaluate steady-state properties of LEA in relation to two parent proteins, in addition to a single-site mutant and a derivative engineered by incorporating a noncanonical amino acid (ncAA) into the chromophore structure. This unique series of FPs culminate in five biomolecules each with distinct photophysical and photochemical properties that display diverse behaviors upon light irradiation. Structural characterization of the typically dominant resting state with a green *cis*-anionic chromophore was enabled by ground-state femtosecond stimulated Raman spectroscopy (FSRS) (Fang et al., 2009; Dietze and Mathies, 2016; Fang and Tang, 2020), which provides complementary information to the recently acquired crystal structures of LEA-A69T (PDB ID: 8THS) and LEA-H62X (PDB ID: 8UB6). Ultrafast spectroscopy on the femtosecond (fs)-to-picosecond (ps) timescale is key to understanding the primary excited-state events *in situ* during photoswitching (Fron et al., 2007; Colletier et al., 2016; Coquelle et al., 2018; Laptenok et al., 2018; Tang et al., 2021; Tang and Fang, 2021) and photoconversion (Hosoi et al., 2006; Roy et al., 2011; Fron et al., 2013; Fron et al., 2014; Makarov et al., 2014; Coughlan et al., 2021; Addison et al., 2023; Krueger et al., 2023b; Krueger et al., 2023c), besides the fundamental fluorescence process (Fang et al., 2009; Meech, 2009; Tonge and Meech, 2009; van Thor, 2009; Zimmer, 2009; Taylor et al., 2019; Jones et al., 2021), wherein a succinct comparison between LEA and LEA-H62X reveals strikingly similar electronic transient dynamics upon excitation of the anionic green chromophore. Finally, the excited-state FSRS measurements of LEA, LEA-A69T, and ALL-Q62H elucidate the nonequilibrium structural changes on ultrafast timescales. We envision that such mechanistic characterization with chemical bond precision and sub-ps temporal resolution of a unique set of contrasting FPs will be broadly applicable to green-to-red pcFPs, especially those that display both photoconvertible and photoswitchable properties with greater potential to advance bioimaging and life science applications.

## 2 Materials and methods

### 2.1 Protein sample preparation and X-ray crystallography

Following the published protocols, the Wachter Lab prepared the engineered FP samples including LEA, ALL-GFP, and ALL-Q62H (Kim et al., 2013; Kim et al., 2015). Details for the preparation and X-ray crystallography of LEA-A69T and LEA-H62X protein variants are presented below (Sections 2.1.1–2.1.4). The FP samples were shipped from ASU to OSU and then stored in a −80°C freezer (U535 Innova, Eppendorf). Prior to all the steady-state or time-resolved spectroscopic experiments reported in this work, the samples were gradually thawed in a −4°C fridge before warming to room temperature (22°C). Special care was taken to limit the samples from ambient/fluorescent light exposure before experiments. To remove precipitate and reduce aggregated proteins, the samples went through a 0.22 µm filter prior to spectral data collection to reduce laser light scattering. When necessary, the protein sample was centrifuged to form a precipitate pellet and the resulting supernatant was pipetted and used for future experiments with further reduced light scattering. All spectroscopic measurements were performed in a pH 7.9 buffer solution: 50 mM 2-[4-(2-hydroxyethyl)-1-piperazinyl]ethanesulfonic acid (HEPES)-NaOH with 150 mM NaCl and 0.1 mM EDTA.

#### 2.1.1 Molecular cloning and mutagenesis

A synthetic LEA construct containing a C-terminal 6xHis-tag and the H62TAG mutation, with His62 mutated to the amber stop codon, was ordered from Blue Heron Biotech, LLC and inserted between the XbaI and EcoRI sites of pET28a(+). Primers were designed and ordered from Integrated DNA Technologies, Inc. to introduce the A69T mutation into the parent LEA pET28a(+) plasmid using a modified Quick Change method (Xia et al., 2015). Plasmid sequences were confirmed by Sanger sequencing (Azenta Life Sciences).

#### 2.1.2 Protein expression and purification of LEA-A69T and LEA-H62X (X = 3 mH)

Chemically competent *Escherichia coli* BL21*(DE3) cells were heat-shock transformed with the pET28a(+) LEA-A69T plasmid or co-transformed with pET28a(+) LEA-H62TAG and pEVOL_PylRS(NMH), recovered for 1 hour in Super Optimal Broth with Catabolite repression (SOC) media, plated on Luria-Bertani (LB) Broth + 50 μg/mL kanamycin + agar or 50 μg/mL kanamycin (Kan) + 34 μg/mL chloramphenicol (Cam) + agar, respectively; then grown overnight at 37°C. The pEVOL_PylRS(NMH) plasmid codes for an engineered *Methanosarcina mazei* pyrrolysyl-tRNA synthetase (mmPylRS) containing the L305I, Y306F, L309G, C348F, and Y384F mutations (Xiao et al., 2014; Green et al., 2016), which allow the incorporation of *N*_δ_-methyl histidine (NMH) hereafter referred to as 3-methyl-histidine (3 mH) for LEA-H62X. Single BL21*(DE3) transformant colonies were used to inoculate 5 mL 2×YT (Yeast Extract Tryptone medium) + 50 μg/mL kanamycin (2×YT + Kan) or, in the case of co-transformants, 2×YT + 50 μg/mL kanamycin +34 μg/mL chloramphenicol (2×YT + Kan + Cam) cultures were then grown at 37°C and 250 rpm overnight. For LEA-A69T expression, overnight cultures were transferred to a 2.8 L baffled Fernbach flask containing 1 L 2×YT + Kan and growth was continued at 30°C and 250 rpm until the O.D._600_ (optical density at 600 nm) reached 0.6. Isopropyl β-D-1-thiogalactopyranoside (IPTG) was then added to a concentration of 1 mM and growth continued for 16 h at 30°C and 180 rpm. LEA-H62TAG overnight cultures were pelleted at 7,808 g for 15 min, the supernatant decanted and the pellet suspended in 2 mL of 2×YT + Kan + Cam, subsequently transferred to a 1 L baffled Erlenmeyer flask containing 200 mL of 2×YT + Kan + Cam supplemented with 0.1 M tris(hydroxymethyl)aminomethane (Tris)-HCl pH 7.5, the growth continued at 30°C and 250 rpm until the O.D._600_ reached 0.6. Therein, 3 mH was added to a concentration of 2 mM and growth continued for 15 min. L-arabinose was added to 0.2% (w/v) and growth continued for 15 min. IPTG was then added to a concentration of 1 mM and growth continued for 16 h at 30°C and 180 rpm. The overnight expression cultures were pelleted by centrifugation at 7,808 g for 15 min, the supernatant removed, the pellets thoroughly suspended in 4 mL 25 mM Tris-HCl pH 8.0 + 20 mM NaCl + 10 mM imidazole pH 8.0 + 0.1 M ethylenediaminetetraacetic acid (EDTA) per gram of wet cell paste and the suspended pellets frozen at −80 °C.

The frozen cell pellets were thawed on ice. Phenylmethylsulfonyl fluoride (PMSF) prepared at 100 mM in isopropyl alcohol was added to a final concentration of 1 mM and hen egg white lysozyme was added to 0.25 mg/mL. The thawed pellet suspensions were incubated at room temperature with agitation for 20 min. Cells were lysed by sonication and the lysate clarified via centrifugation at 48,889 g for 45 min at 4 °C. The supernatant was then separated from the cellular debris, 5 M NaCl was added to a final concentration of 300 mM and 2-mercaptoethanol (2 ME) was added to 1 mM. The LEA-A69T and LEA-H62X (where X = 3 mH) variants were purified from the supernatant using Hi-Trap Ni-NTA (Cytiva) affinity chromatography columns with elution in a gradient of imidazole. Fluorescent fractions eluted in high imidazole concentration (>100 mM), were pooled and dialyzed into 20 mM 2-(N-morpholino)ethanesulfonic acid (MES) pH 5.5 + 10 mM NaCl before further purification using a Hi-Trap SP HP (Cytiva) cation exchange chromatography column. Eluted samples were centrifugally concentrated and dialyzed into 50 mM HEPES-NaOH pH 7.9, 150 mM NaCl, 0.1 mM EDTA, and 1 mM dithiothreitol (DTT) for spectroscopic studies or 10 mM HEPES-NaOH pH 7.5 + 75 mM NaCl for crystallization (see below). During all the protein expression, purification and storage steps, samples were kept wrapped in aluminum foil as much as possible to minimize exposure to ambient light. The efficiency of 3 mH incorporation within the LEA-H62X variant was assessed by electrospray ionization quadrupole time-of-flight mass spectrometry (ESI-QTOF-MS) using an Agilent 6530 Quadrupole TOF LC/MS system (Agilent Technologies, Inc.), see Section 3.2 below and the Supplementary Material for more discussions about the protein chromophore maturation.

#### 2.1.3 Protein crystallization, diffraction data collection and processing

Crystals of LEA-A69T grew at room temperature by hanging drop vapor diffusion with drops containing 2 μL of protein at 20 mg/mL in 10 mM HEPES-NaOH pH 7.5 + 75 mM NaCl and 2 μL of well solution containing 30%–35% polyethylene glycol (PEG) 3350, 0.1–0.3 M potassium nitrate. LEA-H62X was crystallized by hanging drop vapor diffusion at room temperature with drops containing 2 μL of protein at 15 mg/mL in 10 mM HEPES-NaOH pH 7.5 + 75 mM NaCl and 2 μL of well solution containing 23%–27% PEG 3350 + 0.1–0.3 M magnesium chloride. Crystallization trays were wrapped in aluminum foil immediately following setup to minimize light exposure. Prior to flash freezing with liquid nitrogen, single crystals were transferred to a solution of either 40% PEG 3350 + 0.2 M potassium nitrate (for LEA-A69T) or 40% PEG 3350 + 0.2 M magnesium chloride (for LEA-H62X) and equilibrated in the dark for 20 min. X-ray diffraction data were collected at 100 K, ambient pressure on Stanford Synchrotron Radiation Lightsource (SSRL) Beamline 9-2 with a Dectris Pilatus 6M detector. The crystallographic data were processed using the XDS software package (Kabsch, 2010).

#### 2.1.4 Structure determination and refinement

Structures were determined by molecular replacement using the program Phaser (McCoy et al., 2007) with Protein Data Bank (PDB) entry 4GOB (Kim et al., 2013) as the search model. Crystallographic models were constructed from iterative cycles of model building using the program Coot (Emsley and Cowtan, 2004) and refinement using REFMAC (Murshudov et al., 1997). The figures of the crystallographic models were made using PyMOL molecular graphics software (Schrödinger, 2012).

### 2.2 Steady-state spectroscopy

**FIGURE 1:**
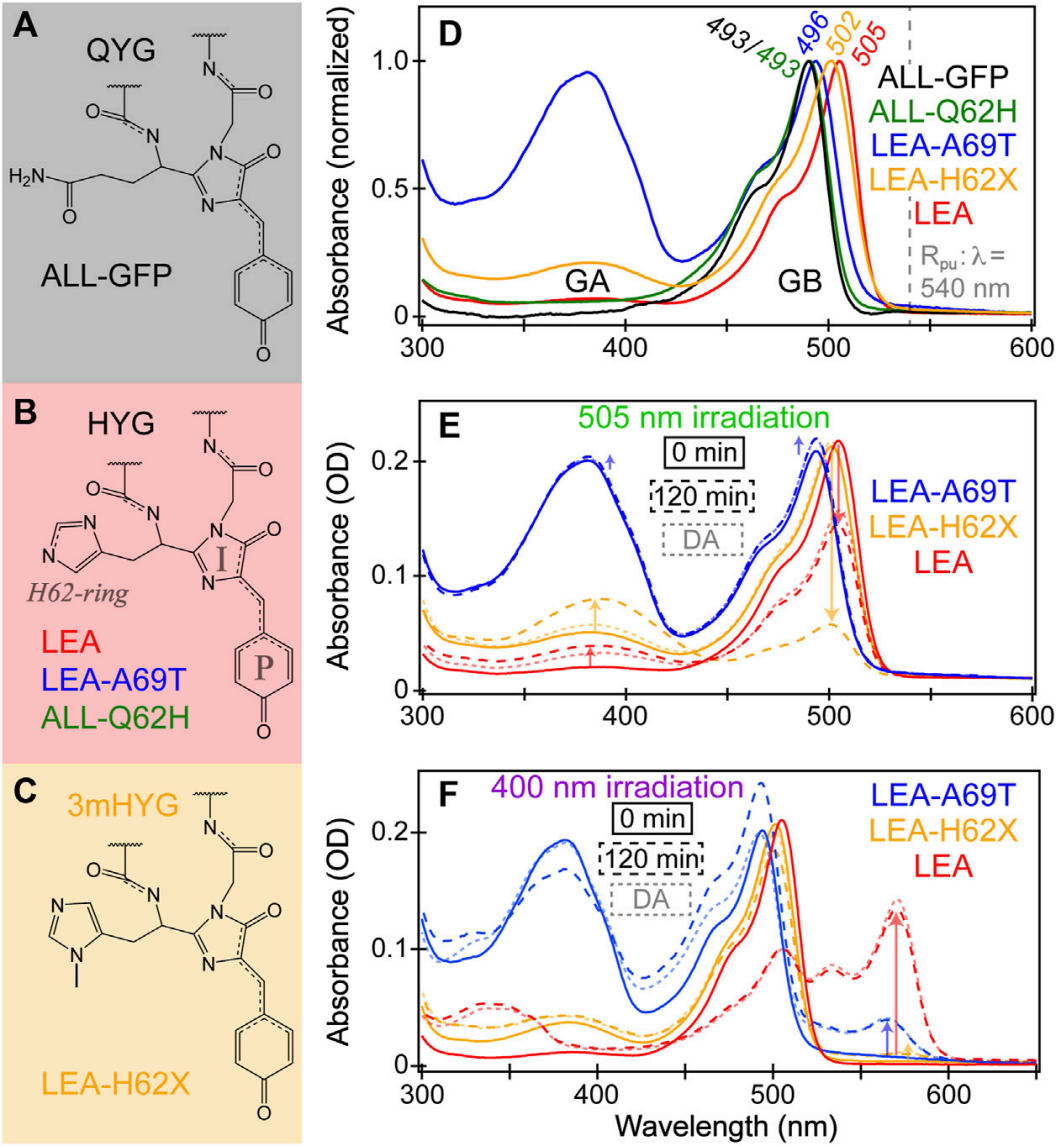
Chromophore structures of **(A)** ALL-GFP with a QYG chromophore, **(B)** LEA, LEA-A69T, and ALL-Q62H with an HYG chromophore, and **(C)** LEA-H62X (X = 3-methyl-histidine, 3 mH) with a 3mHYG chromophore. In panel **(B)**, the phenolate (P)-ring, imidazolinone (I)-ring, and sidechain imidazole ring of histidine residue of the chromophore are denoted in gray. **(D)** Normalized steady-state absorption spectra of ALL-GFP (black), ALL-Q62H (green), LEA-A69T (blue), LEA-H62X (orange), and LEA (red) in pH 7.9 buffer. GA and GB refer to the neutral and anionic green chromophores, respectively. The GB absorption peak wavelengths are listed, and the Raman pump (R_pu_) wavelength is indicated by the dashed gray line. Absorption spectra of LEA-A69T, LEA-H62X, and LEA at 0 minutes (solid) and after 120 min (dashed) of **(E)** 505 nm and **(F)** 400 nm irradiation. The semi-transparent dotted traces display the equilibrated spectral profiles after a 24-h dark adaptation (DA). In **(E, F)**, the arrows highlight the pertinent spectral changes during photoswitching and photoconversion, respectively.

**FIGURE 2:**
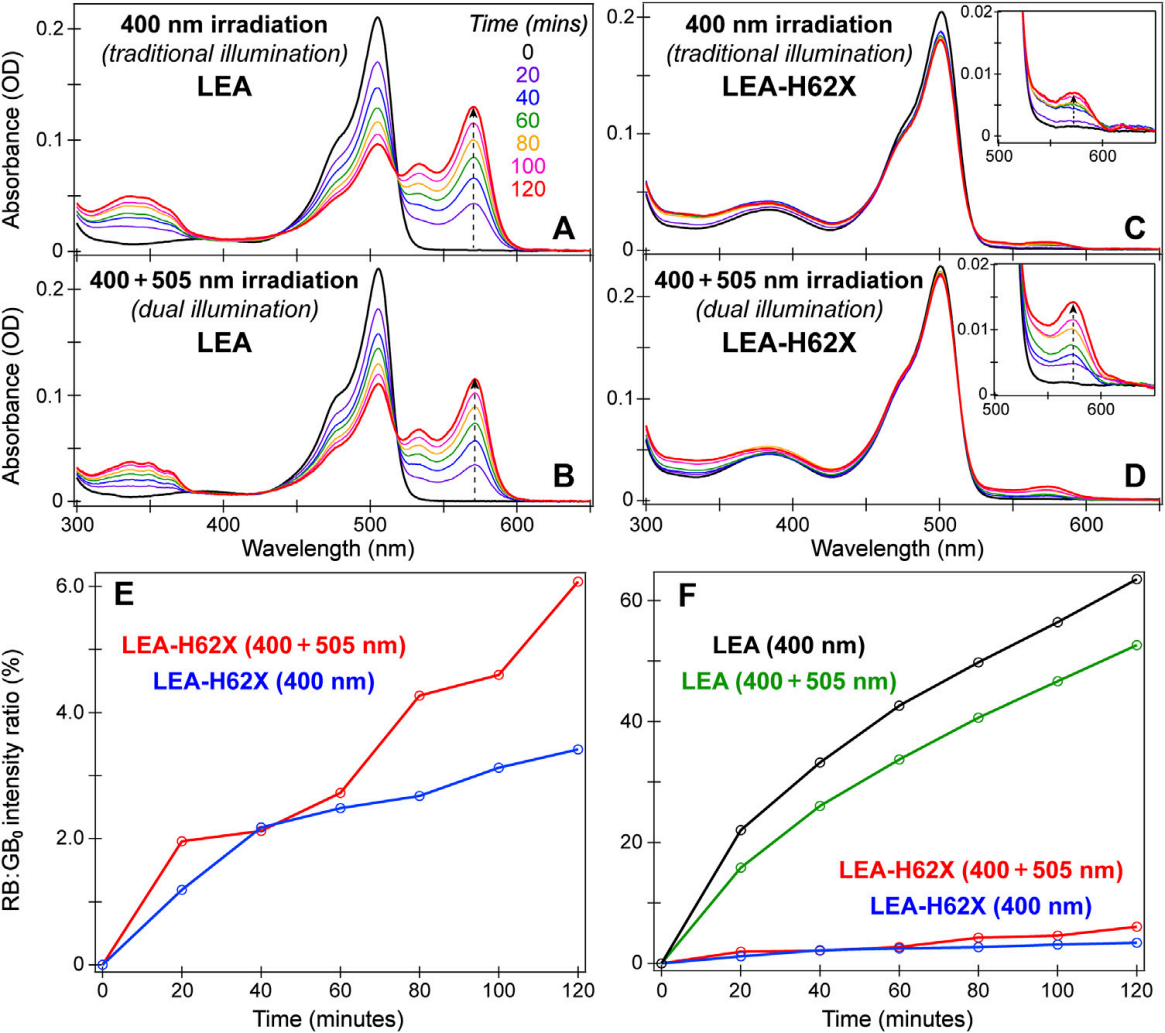
Time-resolved steady-state absorption spectra of LEA **(A, B)** and LEA-H62X **(C, D)** under traditional 400 nm and dual 400 + 505 nm illumination, respectively. The absorption spectra were collected in pH 7.9 buffer solution. The color-coded irradiation times in minutes are listed in **(A)**. In **(C)** and **(D)**, the insets display the rise of the absorption peak from the photoconverted red anionic chromophore. **(E)** Peak intensity ratios of the red anionic chromophore (RB) to the original green anionic chromophore (GB_0_) under dual (red) and traditional (blue) illumination of LEA-H62X at various time points (open circles). The peak intensities were recorded at the respective RB/GB_0_ peak wavelengths. **(F)** The RB:GB_0_ peak intensity ratios of LEA-H62X/LEA under traditional (blue/black) and dual (red/green) illumination. Note that the photoconverting power (i.e., 400 nm LEDs) is doubled in 400 nm irradiation case compared to the dual (400 + 505 nm) illumination case.

The steady-state electronic absorption data were collected using a Thermo Scientific Evolution 201 UV/Visible (UV/Vis) spectrophotometer. The steady-state fluorescence data were acquired using a Shimadzu RF-6000 spectrofluorophotometer. The protein sample was housed in a 1-mm-pathlength quartz cuvette (Spectrosil 1-Q-1, Starna Cells, Inc.) and a four-sided rectangular quartz cuvette with a 5-mm pathlength, respectively, for the absorption, excitation, and emission measurements at room temperature (see various spectral data plots in Figures 1, 2; Supplementary Figures S1, S2).

The wavelength-dependent light-emitting diode (LED) illumination experiments for the time-resolved steady-state electronic absorption measurements were performed in a homebuilt 3D-printed LED box to monitor the photoconversion and photoswitching in a controlled environment (see Figures 1, 2; Supplementary Figures S1, S2). The details regarding the LED box and incident light conditions on the sample were reported; in particular, the box dimensions are 10 × 10 × 6 cm^3^ (L × W × H) with the sample stored in a vial or cuvette which was held in place by a ring stand for even illumination (Krueger et al., 2020). One through-hole LED was placed in a 5-mm-diameter hole centered on each face (four in total) with each LED (four in total) ∼5 cm from the box center where the sample was positioned.

The LEDs were wired in series to maintain a constant current running through and to enable precise control of the LED emitting power by adjusting the voltage from the power supply (Tektronix CPS250). Two LED types were used with different emitting wavelengths, ∼400 and 505 nm. The 400 nm LEDs (Bivar, Inc.) were used for photoconversion experiments including traditional (400 nm only) and dual (400 + 505 nm) illumination. The tight tolerance (±2.5 nm) 400 nm LEDs require an optimal voltage and current of 3.4 V and 20 mA per bulb to give the best performance. To achieve photoconversion with four 400 nm LEDs and to match the experimental conditions for the previous reports (Krueger et al., 2020; Krueger et al., 2023b), the power supply was tuned to a total voltage of ∼13 V and 20 mA, resulting in a measured emitting power of ∼9 mW per bulb and 36 mW total at the sample spot. The 505 nm LEDs (Avago Technologies) were used for the photoswitching (505 nm only) and dual-illumination measurements. The 505 nm LEDs have a broader spectral bandwidth (±30 nm) and require 3.2 V and 30 mA per bulb for optimal performance. For the photoswitching measurements, four 505 nm LEDs were used with the power supply set to ∼13 V and 30 mA, resulting in a measured emitting power of ∼9 mW per bulb and 36 mW total at the sample spot. For the dual-illumination measurements, two 400 nm LEDs and two 505 nm LEDs were used and placed on opposite faces of the 3D box. The power supply was then set to ∼13 V and 25 mA resulting in a measured emitting power of ∼9 mW per bulb and 36 mW total at the sample spot. The LED powers were measured using a Thorlabs power meter with a S302C Thermal Power Sensor Head at the sample position. The average LED power density from all four LEDs was estimated to be ∼0.05 W/cm^2^ at the sample spot, which can be considered gentle; for example, it is much lower than the 7 W/cm^2^ irradiation power density at 488 nm illumination in a previous study on a related pcFP mEos4b (De Zitter et al., 2020).

### 2.3 Femtosecond transient absorption (fs-TA) and femtosecond stimulated Raman spectroscopy (FSRS)

The ultrafast spectroscopic measurements were conducted using a homebuilt nonlinear optical setup, a detailed description can be found elsewhere (Zhu et al., 2014; Han et al., 2016; Liu et al., 2016; Chen et al., 2018; Tang et al., 2018). In brief, the fundamental laser output originates from a Ti:sapphire regenerative amplifier (Legend Elite-USP-1K-HE, Coherent, Inc.) that is seeded by a mode-locked Ti:sapphire oscillator (Mantis-5, Coherent, Inc.) to produce a pulse train (∼800 nm center wavelength, ∼35 fs pulse duration) with an average power of ∼3.6 W operating at a 1 kHz repetition rate. The actinic pump wavelength was tuned to 490 nm for all the fs-TA and excited-state (ES)-FSRS measurements on LEA and ALL-FPs. For the tunable actinic pump generation, a two-stage noncollinear optical parametric amplifier (NOPA) was followed by a chirped-mirror pair (DCM-12, 400–700 nm, Laser Quantum, Inc.) to achieve an output pulse duration below ∼100 fs full-width-at-half-maximum (fwhm).

The tunable Raman pump was generated by an fs-NOPA that generates the seed pulse at the desired wavelength, followed by a two-stage ps-NOPA to amplify the beam to an appreciable power for FSRS data collection. For both the ground-state (GS) and ES-FSRS, a 540 nm Raman pump was used for all the FPs studied. The broadband probe for both fs-TA and FSRS was generated by focusing a small portion of the ∼800 nm fundamental pulse onto a 2-mm-pathlength quartz cuvette filled with deionized water to obtain a stable supercontinuum white light. The probe was compressed via a chirped mirror pair (DCM-9, 450–950 nm, Laser Quantum, Inc.) to a pulse duration <100 fs (Tang et al., 2018).

For fs-TA measurements, the actinic pump and probe beams were directed onto a parabolic mirror to focus onto the sample housed in a 1-mm-pathlength quartz cuvette. For GS-FSRS, the Raman pump and probe were incident on the sample; whereas for ES-FSRS, the actinic pump (A_pu_), Raman pump (R_pu_), and Raman probe (R_pr_) were all directed onto the sample. A chopper was placed in the A_pu_ beampath during fs-TA measurements, albeit in the R_pu_ beampath for GS and ES-FSRS measurements. Additionally, for ES-FSRS, an electronic shutter was placed in the A_pu_ beampath for signal generation. In all cases, the chopper operated at half the frequency (i.e., 500 Hz) of the fundamental laser repetition while the phase was adjusted to maximize the signal intensity according to the data collection scheme. Post the sample cell, the R_pr_ beam was collimated and focused into a spectrograph (IsoPlane SCT-320, Princeton Instruments, Inc.) that used a reflective grating (300 grooves/mm, 300 nm blaze wavelength for fs-TA; 1200 grooves/mm, 300 nm blaze wavelength for FSRS) for dispersion onto a CCD array camera (PIXIS:100F, Princeton Instruments, Inc.) to image and collect data at the exit focal plane. For the wavelength axis (in nm unit) and frequency axis (in cm^–1^ unit) calibration, a mercury:argon lamp was used for fs-TA and the cyclohexane solvent was used as the standard for FSRS, respectively.

**FIGURE 3:**
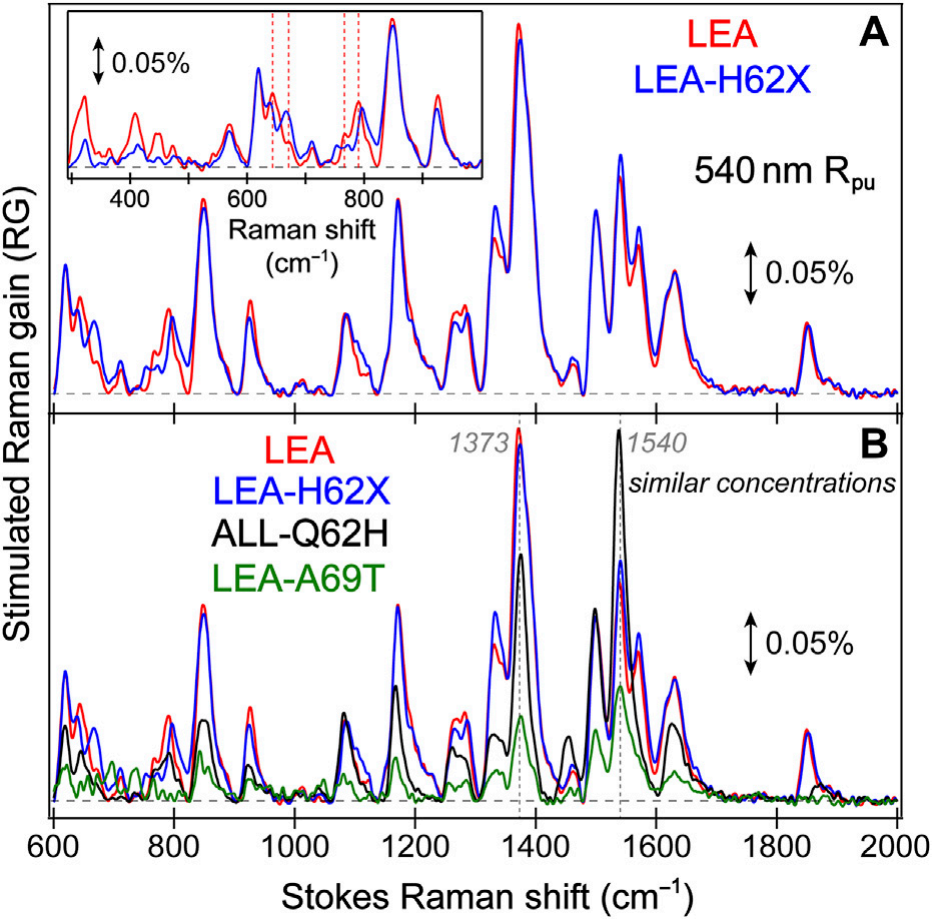
Ground state (GS)-FSRS spectra of **(A)** LEA (red) and LEA-H62X (blue), and **(B)** LEA, LEA-H62X, ALL-Q62H (black), and LEA-A69T (green) collected with a 540 nm Raman pump (R_pu_, ∼2 mW average power) and a redder Raman probe on the Stokes side. The Raman gain (RG) magnitude of 0.05% is indicated by the double-sided arrow. The inset in **(A)** displays the GS-FSRS spectra of LEA and LEA-H62X from ∼300 to 1000 cm^−1^ with vertical dashed lines to highlight shifted peak frequencies. The horizontal dashed lines denote the zero-intensity level. Two vertical dotted gray lines in **(B)** denote two prominent vibrational marker bands.

For the fs-TA and ES-FSRS measurements, the 490 nm A_pu_ power was set to an average power of ∼0.25 mW. For GS-FSRS, the 540 nm R_pu_ power was set to ∼2 mW, and the experimental Raman spectra and mode frequencies are compared in Figure 3; Supplementary Table S1, respectively. For ES-FSRS, the 540 nm R_pu_ power was set to ∼2.5 and 4.0 mW on average. The ES-FSRS data were intentionally collected with these two pump powers to compare the R_pu_ power influence on transient vibrational intensity dynamics (see Section 3.3 below). The sample optical density (OD) for fs-TA experiments on LEA and LEA-H62X was ∼0.5/mm at 500–505 nm. The OD for the GS-FSRS measurements on LEA, LEA-H62X, and ALL-Q62H was ∼1.0/mm at 490–505 nm, and ∼0.4/mm at 495 nm for LEA-A69T due to its elevated p*K*_a_. To achieve high signal-to-noise ratio, the OD for the ES-FSRS measurements on LEA was ∼1.1/mm, LEA-A69T was ∼1.0/mm, and ALL-Q62H was ∼1.1/mm at the GB absorption peak (i.e., 490–505 nm). The protein samples, except for LEA-H62X, were continuously flowed during ultrafast spectroscopic experiments to ensure sample stability using a home-built peristaltic pump and a 1-mm-pathlength quartz cuvette (48-Q-1, Starna Cells, Inc.). The limited sample quantity due to low expression yields (∼1 mg of protein per L of expression culture) of the ncAA-incorporated LEA-H62X did not produce enough proteins to be continuously flowed at the necessary volume and OD for ultrafast measurements; therefore during ultrafast spectroscopy, LEA-H62X was continuously stirred using a magnetic stir bar and motor. The cross-correlation time of the optical setup was ∼120 fs, enabling us to capture the excited-state spectral features starting from the Franck-Condon region accessed by photoexcitation and to retrieve initial temporal components on the ∼100 fs timescale (Dietze and Mathies, 2016; Fang et al., 2018; Fang and Tang, 2020).

Notably, each GS-FSRS spectrum (Figure 3) is the average of 75,000 spectra (1,500 per set × 50 sets). Moreover, we compared the sample-to-sample variability from different protein batches for LEA, LEA-A69T, and ALL-Q62H. These spectra can be found in literature (Krueger et al., 2020; Krueger et al., 2023b), and while there are some differences due to resonance conditions incurred by varying Raman pump wavelengths (which again hints at the sensitivity of FSRS) (Dietze and Mathies, 2016; Fang et al., 2019), the Raman peak frequencies vary by 1–2 cm^–1^ while maintaining similar peak patterns and intensities. The cases where the peak frequencies, intensities, or patterns vary significantly between the reported GS-FSRS spectra and current work were explicitly mentioned (see Section 3.2 below). For each fs-TA or ES-FSRS measurement, six separate datasets with all the time delay points were collected back-to-back and averaged. Moreover, the UV/Vis spectra were collected before and after each measurement to monitor the protein sample stability under various laser irradiation conditions, which typically yield <5% spectral change for our line of inquiry using a flow system (Fang et al., 2009; Fang and Tang, 2020) or the stirring sample solution for LEA-H62X.

### 2.4 Quantum calculations and PyMOL illustration

The quantum chemical calculations were performed using Gaussian 16 software (Frisch et al., 2016) to calculate the ground-state Raman mode frequencies and intensities of the anionic HYG chromophore *in vacuo*. Density functional theory (DFT) was used with the RB3LYP functional and 6-311G+(d,p) basis sets. The chromophore was capped with peptide bonds and methyl groups at both ends to mimic the protein environment in a computationally feasible manner. Two types of calculations were conducted and compared. The first one took the chromophore structure directly from crystal structures of LEA (PDB: 4DXN), LEA-A69T (PDB: 8THS), and LEA-H62X (PDB: 8UB6), and ran a frequency calculation to predict the vibrational normal mode frequencies without any further geometrical optimization. The second set of calculations took the chromophore structure directly from the crystal structures of LEA, LEA-H62X, and LEA-A69T, and then performed a DFT-based geometrical optimization. In particular, three dihedral angles around the $C_{\alpha }-C_{\beta }$ bridge between the I-ring and His62 (see Figure 1B below for the ring labels) were confined to represent the mostly planar structure of the chromophore in the protein pocket. Without this confinement *in vacuo*, the His62 sidechain ring would rotate to a nonplanar conformation that is unrealistic in the protein matrix (Krueger et al., 2023b). All the other coordinates were left free for the optimization step. Following the optimization, the vibrational normal mode frequencies and intensities were calculated. The output Raman spectra were displayed and compared in Supplementary Figures S3–S5 and tabulated in Supplementary Tables S2–S5 including the major mode assignment. A frequency scaling factor of 0.98 was used for the optimized structures’ calculated frequencies (Merrick et al., 2007; Taylor et al., 2019), while no frequency scaling factor was used for the calculated frequencies directly from the crystal structures without optimization (Supplementary Figure S5). The PyMOL software (Schrödinger, 2012) was used to visualize the chromophore and local environment residues on the basis of the aforementioned LEA, LEA-H62X, and LEA-A69T crystal structures (PDB atomic coordinates).

## 3 Results and discussion

LEA was engineered to represent the evolutionary node that acquired the unique ability to photoconvert from the ancestral green fluorescent protein (ALL-GFP), which was engineered on the basis of ancestral gene reconstruction from the great star coral *Montastraea cavernosa* (Ugalde et al., 2004; Field and Matz, 2010; Kim et al., 2013; Kim et al., 2015). ALL-GFP possesses a Gln-Tyr-Gly (QYG) chromophore (Figure 1A) and cannot photoconvert. Perhaps the most important mutation to make LEA photoconvertible is modifying the chromophore to a His-Tyr-Gly (HYG) chromophore, making it a Kaede-type FP. Kaede, Japanese for Maple leaf, was the first green-to-red FP discovered from the stony coral *Trachyphyllia geoffroyi* (Ando et al., 2002). A majority of green-to-red pcFPs possess an HYG chromophore which is considered crucial to maintain the photoconversion ability. However, the incorporation of His62 into the chromophore-bearing tripeptide via the Q62H mutation does not make ALL-Q62H photoconvertible (Figure 1B). In fact, 12 other mutations and one deletion from ALL-Q62H were necessary to make the resultant LEA photoconvert efficiently (Kim et al., 2013). About half of these mutations are in close proximity to the chromophore, many of these mutations involve the replacement of large amino acids with smaller ones to promote the chromophore flexibility. The corroborating molecular dynamics (MD) simulations found that LEA possesses a more spacious and flexible local environment around the chromophore compared to ALL-Q62H (Kim et al., 2015).

A notable reverse single-site mutant (LEA-A69T) involves modifying Ala69 back to Thr69, which ALL-GFP and ALL-Q62H both possess. The presence of Ala69 was identified early on as another key residue to maintain the photoconversion efficiency (Kim et al., 2013; Krueger et al., 2023b). Interestingly, this residue has been implicated to play an important role in not only photoconversion, but also photoswitching of LEA and many other FPs (Henderson and Remington, 2005; Nienhaus et al., 2005; Remington et al., 2005; Kikuchi et al., 2008; Adam et al., 2009; De Zitter et al., 2020; Nienhaus and Nienhaus, 2021; Krueger et al., 2023b). To round out the set of related FPs, a new FP with a novel chromophore via ncAA incorporation is presented as LEA-H62X, wherein X = 3-methyl-histidine (Figure 1C). The modification of His62 was specifically performed to help address some lingering questions about the photoconversion mechanism. We note that all the FPs in this work contain the same Tyr63-Gly64 chromophore unit with alterations of its first residue (X62).

### 3.1 Tracking photochromism of phototransformable fluorescent proteins under various illumination conditions with steady-state electronic spectroscopy

The steady-state electronic absorption spectra of FPs (Figure 1D) exhibit subtle changes due to the aforementioned modifications. The anionic green chromophore, termed GB, of ALL-GFP and ALL-Q62H both absorb the bluest at ∼493 nm, indicating that the unconjugated first residue (i.e., Q62 or H62) does not significantly affect the absorption of green deprotonated chromophore, although ALL-Q62H does display a more pronounced vibronically-coupled shoulder at ∼460 nm. The GB form of LEA absorbs the reddest at ∼505 nm, with LEA-A69T (496 nm) and LEA-H62X (502 nm) lying in between the ALL-FPs and LEA. Several possible reasons for the observed shift in the GB absorption can be summarized by a unifying theme: the absorption band can be blueshifted by stabilizing the negative charge on the P-ring or destabilizing the negative charge on the I-ring (Henderson and Remington, 2005; Adam et al., 2009; Lin et al., 2019; Chen et al., 2023). The chromophore within green fluorescent protein (GFP) and many other FPs is known to display charge transfer from the phenolate (P)-ring to the imidazolinone (I-ring, see Figure 1B for ring labels) upon photoexcitation (Kummer et al., 2002; Litvinenko et al., 2003; Martin et al., 2004; Altoè et al., 2005; Olsen and Smith, 2008; Chen et al., 2019; Taylor et al., 2019; Jones et al., 2021; Addison et al., 2023). Charge transfer (CT) is well-known to redshift the absorption spectra of chromophores, so by stabilizing the charge on P-ring or destabilizing the charge on I-ring, the CT magnitude is reduced and thus the absorption band shifts to the blue. This trend is also observed in steady-state excitation and emission spectra of LEA, LEA-H62X, and LEA-A69T (Supplementary Figure S1A, B), where the emission peak wavelengths are the reddest for LEA and bluest for LEA-A69T. Further evidence for the varying CT magnitudes will be discussed in relation to the recently acquired protein crystal structures (see below).

The neutral green chromophore (GA) absorbs below 400 nm and is most visible in LEA-A69T and LEA-H62X. The A69T mutation has been documented to cause large p*K*_a_ changes (Adam et al., 2009; Berardozzi et al., 2016; De Zitter et al., 2020), which is clearly the case for LEA-A69T (p*K*_a_ = 8.4) *versus* LEA (p*K*_a_ = 6.3) (Kim et al., 2013; Krueger et al., 2023b). Interestingly, the incorporation of an extra methyl group in LEA-H62X leads to an elevated p*K*_a_ compared to LEA (Figure 1D). The protonated chromophore is often referred to as a dark state due to its weak fluorescence; in Kaede-like pcFPs, GA exists nearly exclusively in the *trans* conformation that resides in an undeveloped H-bond network relative to a “resting” network experienced by the *cis* conformation of the bright anionic chromophore (Wachter et al., 2010; Shcherbakova et al., 2012; Subach and Verkhusha, 2012). In addition to the reduced oscillator strength of a *trans* chromophore, the protonated population prefers nonradiative relaxation after photoexcitation, which include excited-state proton transfer (ESPT), Förster resonance energy transfer (FRET), and ring-twist-induced isomerization or internal conversion (Hosoi et al., 2006; Meech, 2009; Roy et al., 2011; Fron et al., 2013; Fron et al., 2014; Fang and Tang, 2020; Krueger et al., 2023b). The weak fluorescence band from GA* appears around 440–450 nm (Supplementary Figures S1C, D), while the dominant emission still occurs from GB*, implying that ESPT, FRET, and/or isomerization occurs upon excitation of the neutral chromophore population (i.e., GA). Note that we use the “neutral” and “protonated” chromophore interchangeably to compare with the “anionic” and “deprotonated” chromophore, highlighting the protonation state at the chromophore’s P-ring end.

A previous investigation discovered that LEA was capable of photoswitching upon 505 nm illumination (Krueger et al., 2020). Photoswitching typically involves conformational change of the chromophore; in particular, *cis*↔*trans* isomerization of the P-ring represents the most common photoswitching mechanism to result in a p*K*_a_ increase/decrease and thus a larger population of the protonated/deprotonated chromophore (Nienhaus and Nienhaus, 2014; Tang and Fang, 2022). The *on*→*off* photoswitching of LEA under 505 nm illumination qualifies as negative photoswitching, where the FP chromophore population directly under illumination goes away. This behavior can be viewed spectroscopically in Figure 1E, where the GB absorption peak displays a moderate decrease after 2 hours of gentle 505 nm LED illumination with a concomitant increase of the protonated chromophore. Interestingly, the photoswitched *off* state of LEA is remarkably long-lived, only showing a minor return to original state upon dark-adapt equilibration for 24 h. The lack of spontaneous re-equilibration of the photoswitched, essentially permanently trapped *off* state (unless under another light irradiation) invoked a new chromophore population, GA’, referring to this much longer-lived photoswitched green neutral chromophore. The GA’ state of LEA is unique when compared to many other photoswitchable chromophores, especially in Kaede-like pcFPs, since their many photoswitched long-lived *off* states eventually return to the original resting state (Nienhaus and Nienhaus, 2014; Berardozzi et al., 2016; De Zitter et al., 2020; Krueger et al., 2020; Tang and Fang, 2022). In the case of LEA, the GA’ state never fully recovers the GB population spontaneously, even over the course of days to weeks, implying that this long-lived state differs from GA because the chemically (pH)-induced GA would otherwise eventually equilibrate back to GB at the same buffer pH. Further evidence for differentiating GA/GA’ includes that the GA’ absorption peak is redshifted from the original GA absorption peak after 2 hours of 505 nm illumination (Figure 1E, red) and the GA’ species is significantly more prone to photoswitch back to GB compared to the GA species, observed in both steady-state and transient absorption spectroscopies. The only way to switch GA’ back to GB in LEA after 505 nm illumination is through 400 nm illumination, which rapidly recovers the original GB population within minutes (Supplementary Figure S2). This is reminiscent of many photoswitchable FPs, where the *on*→*off* photoswitching is inefficient with a low photoswitching quantum yield (psQY) *versus* the efficient *off*→*on* transition with a high psQY (Bourgeois and Adam, 2012; Nienhaus and Nienhaus, 2014). A detailed comparison of the *off*→*on* photoswitching and photoconversion of the chemically- and light-induced protonated species within LEA and related mutants will be the topic of a future investigation.

Currently, there is no crystal structure available for the photoswitched *off* state in LEA; however, the GA’ chromophore structure may reside in a *trans*-like distorted structure reminiscent of IrisFP and mEos4b (Adam et al., 2008; Berardozzi et al., 2016; Colletier et al., 2016; De Zitter et al., 2020). Something may go awry during the *cis*→*trans* isomerization and *on*→*off* photoswitching that traps the protein chromophore in a frustrated conformation. Prior works on the photoswitched *off* states in related pcFPs suggested that the GA’ state of LEA may be stabilized by H-bonds and electrostatic interactions particularly well (De Zitter et al., 2020; Krueger et al., 2020). Interestingly, LEA-A69T is not photoswitchable under 505 nm illumination (Figure 1E, blue), which is considered to be a marker for a primed-convertible FP (Mohr et al., 2016; Mohr et al., 2017). Primed conversion relies on two incident lights to induce photoconversion: a priming wavelength of ∼490 nm and a converting wavelength above 600 nm. Most primed-convertible FPs have a threonine at position 69 and are not photoswitchable, which LEA-A69T satisfies (Mohr et al., 2017). A key difference between traditional photoconversion and primed conversion is that the former case starts with a protonated chromophore as the sole photoconvertible species, whereas the latter case is initiated from the anionic chromophore.

A rather surprising finding is that the 3-methyl-histidine (3 mH)-bearing chromophore in LEA-H62X leads to pronounced *on*→*off* photoswitching (Figure 1E, orange), where the GB absorption peak exhibits a large intensity decrease after 2 hours of 505 nm illumination, significantly more than LEA, together with a large increase in the GA’ population. This is quite unexpected because the extra methyl group on the unconjugated His62 ring is far from the P-ring proposed to undergo *cis*→*trans* isomerization during photoswitching. Three closely related FPs with varying *on*→*off* photoswitching magnitudes, LEA-H62X > LEA > LEA-A69T, offers a promising avenue for ultrafast spectroscopies to delineate key indicators and properties for photoswitching on molecular timescales. Another notable difference between the photoswitching behavior of LEA-H62X and LEA is that the GA’ species within the former FP is much more dynamic than the latter, as it spontaneously recovers the GB population almost fully within the 24-h dark adaptation at thermal equilibrium (Figure 1E). This finding suggests that the GA’ species of LEA-H62X may experience more H-bonding partners and increased polar contacts than the GA’ species of LEA to resemble IrisFP with a faster thermal recovery and higher *on*↔*off* switching both ways (De Zitter et al., 2020). Moreover, the GA’ species within LEA and LEA-H62X likely experience more H-bonding partners than the corresponding GA species, evidenced by the more dynamic *off*→*on* photoswitching of GA’ (see Supplementary Figure S2A, B, D).

Furthermore, the photoconversion efficiency also varies among the three pcFPs (Figure 1F), with LEA displaying the fastest photoconversion rate and largest yield of the red anionic chromophore (RB) with a 573 nm absorption peak. For comparison, LEA-A69T displays a much reduced photoconversion efficiency (∼80% lower) than that of LEA under identical light irradiation conditions, followed by LEA-H62X that manifests a photoconversion yield ∼95% lower than LEA after 2 hours of 400 nm irradiation. In particular, upon 400 nm irradiation of the GA species in LEA-A69T, pronounced *off*→*on* photoswitching from GA to GB species is observed, which is corroborated by the telltale decrease/increase of the neutral/anionic chromophores’ absorption peaks within 5 min (Supplementary Figure S2C), similar to the GA’ states of LEA and LEA-H62X (Supplementary Figure S2B, D). Such behavior is not observed in the chemically-induced neutral GA species in LEA (Supplementary Figure S2A) and LEA-H62X (Figure 1F), revealing that the prominent GA population in LEA-A69T (due to the aforementioned chromophore p*K*_a_ increase) is more prone to photoswitching, likely involving isomerization and ESPT (Krueger et al., 2023b); this GA state with a *trans*-protonated chromophore likely establishes more effective H-bonds at the P-ring end than the GA state in LEA, corroborated by the blueshifted GA absorption peak of LEA-A69T *versus* LEA (Figure 1D) (De Zitter et al., 2020; Krueger et al., 2020; Chen et al., 2023). Interestingly, the red chromophore of LEA after photoconversion displays *on*→*off* photoswitching between the anionic and neutral forms under light irradiation; however, the photoswitched *off* state of the red chromophore can spontaneously recover the bright resting *on* state in contrast to the green photoswitching behavior (Krueger et al., 2020). In addition, there is preliminary evidence that photoexcitation of the neutral red chromophore can transiently form the anionic species on ultrafast timescales. The timescale and mechanism for *off*→*on* photoswitching in both the green/red forms is currently under investigation to determine whether they mainly occur in the ground or excited state(s).

Since 400 nm light can initiate photoconversion from the neutral green chromophore, while 505 nm light can induce *on*→*off* photoswitching to produce a larger neutral green chromophore population, a dual-illumination strategy was proposed to accelerate photoconversion and enhance the red yield (Krueger et al., 2020). In this context, we monitored the photoconversion of LEA and LEA-H62X under traditional (400 nm only) and dual (400 + 505 nm) illumination with steady-state electronic absorption spectroscopy over 2 hours of gentle, low-power LED irradiation (Figure 2). The spectra were collected in a time-resolved manner every 20 min as RB species accumulates. Comparing the photoconversion of LEA and LEA-H62X under traditional illumination (Figures 2A, C) reveals a stark difference and slowdown of the photoconversion rate upon the incorporation of an extra methyl group on the histidine ring in LEA-H62X. Meanwhile, the photoconversion of LEA-H62X becomes notably more efficient under dual (Figure 2D) *versus* traditional (Figure 2C) illumination that is not reflected by comparing the LEA spectra (Figures 2A, B), which substantiates the more pronounced *on*→*off* photoswitching of LEA-H62X under 505 nm light than LEA.

Another interesting difference comparing the time-resolved spectra of LEA *versus* LEA-H62X is that the GA/GA’ absorption region (i.e., ∼375–425 nm) remains stagnant for LEA (Figures 2A, B) whereas it becomes elevated for LEA-H62X (Figures 2C, D). Under traditional illumination of LEA, the rate at which GA is consumed (photoconverted) must be competitive with the chemical equilibrium inducing the GB→GA transition; in fact, the small GA peak increase over 2 h is indicative of a positive photoswitcher. Similarly, the rate at which GA/GA’ is consumed under dual illumination of LEA must still remain competitive with both the chemical equilibrium and GB→GA’ photoswitching induced by the extra 505 nm light. In contrast, under dual illumination of LEA-H62X, the GA/GA’ absorption band increase from the time-zero spectrum can be attributed to the following two reasons. First, the photoconversion rate is clearly slower in LEA-H62X than LEA, so GA/GA’ is consumed less efficiently. Second, the *on*→*off* photoswitching of LEA-H62X is much more efficient than LEA. Therefore, the combination of slower photoconversion and faster photoswitching can lead to a buildup of neutral chromophore species over the course of dual illumination.

A more quantitative examination of the red:green contrast during photoconversion is presented by plotting the ratio of absorbance from the red anionic chromophore (RB) *versus* that of the original green anionic chromophore at time zero (GB_0_) as a function of irradiation time (Figures 2E, F). Notably, the p*K*_a_ difference between LEA and LEA-H62X may underestimate the photoconversion efficiency of LEA-H62X *versus* LEA because relatively more neutral red chromophores (RA) may be formed in the mutant pcFP (but not directly tracked due to spectral overlap with the remaining GB species) while the photoconverted RB species can be undercounted. The RA chromophore absorbs at ∼460 nm, which can be difficult to observe unless the photoconversion is completed or the buffer pH is altered (Krueger et al., 2020). Nevertheless, Figure 2E confirms that dual illumination of LEA-H62X produces a larger red yield and better color contrast than traditional illumination. While the overall illumination power remains similar for the two conditions, the traditional illumination has double the photoconverting power (with four 400 nm LEDs) *versus* dual illumination (two 400 nm LEDs plus two 505 nm LEDs), thus exposing the specific influence of 505 nm illumination on the photoconversion cycle.

While this is an interesting observation on its own merit, the photoconversion efficiency of LEA-H62X still pales in comparison to LEA (Figure 2F), since LEA achieves an RB:GB_0_ intensity ratio of 50%–60% *versus* 3%–6% over 2 hours of illumination of LEA-H62X. Upon comparing the two illumination conditions for LEA, an opposite trend compared to LEA-H62X was observed with traditional illumination outperforming dual illumination. A previous investigation found that with the same photoconverting power (e.g., two 400 nm LEDs), dual illumination (with two additional 505 nm LEDs) produces a 55% greater photoconversion contrast than traditional illumination (Krueger et al., 2020). However, why does traditional photoconversion of LEA outperform dual illumination with the same total power? The multi-faceted answer involves several factors summarized as a shelving effect where the 505-nm-photoswitched GA’ is less prone to photoconvert but more prone to photoswitch back to GB than the native GA population. Furthermore, a close inspection of time-resolved UV/Vis spectra reveals that GA’ is more susceptible to photobleaching that reduces the overall red yield. These factors are all compounded by the less efficient GB→GA’ photoswitching of LEA relative to LEA-H62X (Figure 1E); if LEA displayed the same *on*→*off* photoswitching efficiency as LEA-H62X, the dual-illumination condition would likely have a larger red yield than traditional photoconversion.

The noncanonical incorporation of an extra methyl group on His62 was designed and performed for several reasons. First and foremost, the photoconversion mechanism has been proposed to primarily involve processes between His62 and the local environment resulting in the peptide cleavage and conjugation extension (Tsutsui et al., 2009; Kim et al., 2015). Hence, observing the influence of this chromophore modification on the photoconversion efficiency can provide deep insights. In particular, there are largely two contrasting photoconversion schemes proposed for LEA where His62 either remains planar or twists to facilitate proton transfer and enable photoconversion (Kim et al., 2013; Kim et al., 2015). It was hypothesized that the extra methyl group in LEA-H62X may cause the His62 ring to become twisted, reminiscent of a His62 rotamer that completely abolishes photoconversion in a LEA mutant, LEA-Q38A. Meanwhile, the extra methyl group could inhibit twisting due to the bulkier size, resulting in more steric hindrance or modified electrostatic interactions with the local environment. Given the clearly reduced photoconversion efficiency of LEA-H62X compared to LEA, one of these mechanisms can explain the reduced red yield following the seemingly innocuous incorporation of a methyl group to the nonconjugated moiety of the initial green chromophore structure (GA, GB). The transient electronic dynamics from fs-TA data of LEA and LEA-H62X help to address this question.

There are several benefits to dual illumination that can maintain or exceed the photoconversion efficiency *versus* traditional illumination; most notably, the harmful near-UV (400 nm) irradiation can lead to undesirable processes including the chromophore bleaching and phototoxicity that could induce cell death, which is likely reduced by the usage of lower-energy 505 nm light with a deeper penetration depth (Gurskaya et al., 2006; Premi et al., 2015; Mohr et al., 2016; Krueger et al., 2020). Furthermore, one can always crank up the 400 nm power to achieve the desired photoconversion rate while incorporating the extra 505 nm light for an additional boost. In fact, many imaging schemes have already employed dual illumination by using a 400 nm converting light and a ∼500 nm imaging light for the bright green chromophore (Betzig et al., 2006; Fernández-Suárez and Ting, 2008; Blum and Subramaniam, 2009; Huang et al., 2010; Nienhaus and Nienhaus, 2014; Shcherbakova et al., 2014). Since ∼500 nm illumination can induce the GB→GA’ transformation, the key parameter of red:green contrast is improved during dual illumination because the bright green anionic state is constantly being switched to the weakly fluorescent protonated state in addition to the improved RB yield. Therefore, the purposive dual illumination provides an alternative photoconversion scheme with great benefits that warrants further development and improvement. For example, if the photoswitching efficiency of LEA-H62X can be combined with the photoconversion efficiency of LEA through rational design and chromophore modification, dual illumination can be an even more desirable photoconversion scheme.

### 3.2 Structural characterization of chromophore interactions with the local environment via X-ray crystallography and femtosecond stimulated Raman spectroscopy

To help answer why LEA-H62X can photoswitch so efficiently yet the photoconversion magnitude is greatly reduced, we implemented GS-FSRS to evaluate the bright green anionic chromophore of LEA and the related FPs. FSRS is particularly suitable to study FPs with structural inhomogeneity because the R_pu_ can be tuned to a wavelength near the ground-state absorption peak to pre-resonantly enhance vibrational signal from the species of interest (Dietze and Mathies, 2016; Fang et al., 2018; Fang and Tang, 2020); in this case, a 540 nm R_pu_ was used to enhance the GB signal. There is a remarkable similarity between LEA and LEA-H62X Raman modes (Figure 3A), consistent with both pcFPs possessing the same chromophore except for an extra methyl group on His62 in LEA-H62X. Therefore, any differences in the GS-FSRS spectra are due to the extra methyl group, and the altered chromophore structures with chromophore-environment interactions. The largest differences occur in the low-frequency region (Figure 3A inset and Supplementary Table S1), which mainly involves global skeletal motions of the chromophore. Interestingly, the vibrational peaks below 500 cm^−1^ are noticeably stronger in LEA than LEA-H62X. Quantum calculations predict that the nonconjugated His62 motions lead to several low-frequency peaks (Supplementary Figures S3–S5; Supplementary Tables S2–S5), including the ∼645 cm^−1^ mode that was discussed as a marker band for pcFPs (Krueger et al., 2020; Krueger et al., 2023b). In support of the experimental spectra, quantum calculations reveal that the major differences between the calculated Raman spectra of LEA and LEA-H62X lie in the low-frequency regime, especially between 600 and 850 cm^−1^.

For example, the low-frequency peak assigned to the chromophore P-ring hydrogen out-of-plane (HOOP) and breathing motions is blueshifted in LEA-H62X (793 cm^−1^) relative to LEA (790 cm^−1^), implying a slightly twisted P-ring in the mutant to reduce the chromophore ring coplanarity (Krueger et al., 2020). Moreover, two low-frequency peaks are redshifted in LEA-H62X (668, 643 cm^−1^) compared to LEA (674, 645 cm^−1^). Among them, the experimental peak frequency of LEA-H62X (668 cm^−1^) matches the calculated frequency (668 cm^−1^) assigned to CNC bending with the extra methyl group motions on the 3-methyl-histidine derivative, directly using the crystal structure (Supplementary Table S5). The corresponding peak in LEA observed experimentally (674 cm^−1^) is greatly reduced in intensity and likely involves HOOP motions of His62, matching a calculated frequency of ∼668 cm^−1^ (Supplementary Table S2). In support of this assignment, the predicted Raman activity of the LEA-H62X mode at 668 cm^−1^ is 13 *versus* 0.3 for the 668 cm^−1^ mode of LEA, verifying the experimental trend that the LEA-H62X peak is more than two-fold stronger than that of LEA. Next, the 767 cm^−1^ peak of LEA appears to split into a doublet in LEA-H62X (770, 753 cm^−1^; Figure 3A). The higher-frequency peak can be assigned to I-ring OOP deformation and bending motions of the C–C bridge between I-ring and His62, and the blueshifted frequency in LEA-H62X may reflect the shifted His62X (in crystal structure) away from Glu211 compared to LEA (Kim et al., 2013). Furthermore, the lower-frequency peak of LEA-H62X (753 cm^−1^) appears to have no direct analog in LEA, hence this peak could arise from methyl motions on His62X akin to the 668 cm^−1^ mode (see Supplementary Table S5). Overall, the largely similar vibrational spectra suggest that the chromophore and local environment, especially near the conjugated P- and I-rings that contribute greatly to the FSRS spectra (of GB herein), are conserved with potential differences arising near the His62 ring due to incorporation of the extra methyl substituent. The similar spectra with nuanced differences also imply that the His62 orientation remains unchanged between LEA and LEA-H62X in regard to the ring coplanarity with the rest of the chromophore. This notion is corroborated by the coordinate-dependent quantum calculations that predict more drastic changes to peak frequencies and intensity patterns in the low-frequency region if His62 ring becomes clearly twisted.

In Figure 3B, the GS-FSRS spectra of all four FPs with HYG chromophore are compared with the inclusion of LEA-A69T and ALL-Q62H. In general, the ALL-Q62H and LEA-A69T peaks are weaker due to the resonance conditions with a 540 nm R_pu_ that is farther away from their blueshifted ground-state absorption peaks (Figure 1D). Furthermore, the LEA-A69T spectrum is noticeably weaker due to a greatly reduced GB population at physiological pHs (caused by the increased p*K*_a_ of the chromophore phenolic group). From a birds-eye perspective, the Raman spectra of all four FPs are similar with some exceptions. In LEA and LEA-H62X the ∼1373 cm^−1^ peak is the most intense, while the ∼1540 cm^−1^ peak is the most intense in the other two FPs. The altered peak intensities are largely influenced by the residue at position 69, made apparent by comparing the spectra of LEA and LEA-A69T that only differ by an alanine and threonine, respectively, at this position. The structural origin for this altered intensity pattern can be gleaned from the ∼1540**–**1550 cm^−1^ mode assignment, calculated to primarily involve a C=N/C=O stretching motion on the I-ring. In LEA and LEA-H62X, Arg66 H-bonds to the C=O group on the I-ring; whereas in LEA-A69T and ALL-Q62H, the larger Thr69 causes Arg66 to reorient and H-bond with Thr69 instead of the I-ring C=O group (Krueger et al., 2023b). The H-bonded C=O group in LEA and LEA-H62X constrains the bond stretch and reduces the electric polarizability, resulting in the mode intensity decrease *versus* LEA-A69T and ALL-Q62H. While this marker band is stronger in ALL-Q62H and LEA-A69T, the ∼1570 cm^−1^ peak mainly assigned to the I-ring C=N stretch is nearly absent, in contrast to its clear presence in LEA and LEA-H62X (Figure 3B). This result supports an altered interaction between Arg66 and the I-ring for these two sets of FPs, where the reduced H-bonding between Arg66 and I-ring in ALL-Q62H and LEA-A69T leads to a “merged” I-ring double-bond stretching band with less distinction between the C=N and C=O bonds. Recently, a systematic comparison of various FP chromophores in solution and the protein matrix was studied via GS-FSRS which revealed a prominent double-bond stretching mode (∼1530**–**1565 cm^−1^) that is especially sensitive to the conformations, electronic structures, and chromophore-environment interactions conserved in many biomolecules including the LEA and ALL-FPs (Chen et al., 2023).

Interestingly, the ∼1850 cm^−1^ mode is much stronger in LEA and LEA-H62X than ALL-Q62H and LEA-A69T. Since the latter two FPs contain Thr69, while the former FPs do not, the intensity of this mode should be influenced by residue at position 69. Furthermore, there is a clear frequency variation of this peak (Supplementary Table S1) among the four FPs studied, as well as an intensity “negative” correlation between this peak and the ∼1540 cm^−1^ peak (Figure 3B) while the latter mode consists of the I-ring C=N and C=O stretch (Supplementary Tables S2-5). The calculated spectra do not predict a strong peak at this frequency (Supplementary Figures S3, S5), so the mode assignment remains less definitive. To better understand the GS-FSRS spectra, the LEA and ALL-FPs would benefit from advanced quantum mechanics/molecular mechanics (QM/MM) simulations that account for various interactions with the local environment as well as the inhomogeneous chromophore conformations. The adjacent protein residue(s) that may be affected by the photoexcited chromophore could contribute to a GS-FSRS peak. In addition, to holistically evaluate the photocycle and provide deeper insights into the competition between photoconversion and the *off*→*on* photoswitching, GS-FSRS spectra of the *trans* protonated chromophore (*off* state) needs to be collected under a suitable experimental condition and compared.

The various differences in the photoresponse of LEA, LEA-A69T, and LEA-H62X, as well as subtle differences in the GS-FSRS spectra, can be evaluated and supplemented by the crystal structures of LEA-A69T and LEA-H62X that were collected and compared for the first time. The protein crystal structures reflect the dominant population at physiological pHs, so they represent the anionic green chromophore. LEA-A69T crystallized in space group *P*2_1_2_1_2_1_ with a full tetramer in the asymmetric unit, while LEA-H62X crystallized in *I*222 with a single protomer per asymmetric unit (Supplementary Table S6); both crystal forms were described previously for LEA (Kim et al., 2013). LEA-A69T and LEA-H62X each crystallized in solutions buffered at pH 7.5 from samples kept in the dark (see Section 2.1.3 above) and, based on their respective ground-state absorbance spectra (Figure 1D), both protein structures should contain a mixture of chromophore species in the GA and GB states. Nevertheless, there is no indication of structural heterogeneity within the LEA-A69T and LEA-H62X electron density maps (Supplementary Figure S6) to suggest that the GA/GB ground state acid/base equilibrium is accompanied by large conformational rearrangement of sidechains in the vicinity of the chromophore (primarily in the *cis* state). This useful finding is consistent with previously reported structures of LEA at high and low pH values where significant differences in sidechain positions between the structures were not observed (Kim et al., 2013). Moreover, the crystal structures reveal minimal perturbations to the chromophore environment when compared with high (PDB ID: 4DXN) and low (PDB ID: 4GOB) pH structures (Kim et al., 2013) of the parent LEA (respective α-carbon root-mean-squared deviations *versus* 4DXN and 4GOB of 0.23 and 0.34 Å for LEA-A69T as well as 0.20 and 0.35 Å for LEA-H62X).

Prior to crystallization, ESI-QTOF-MS analysis of the LEA-H62X sample (see Section 2.1.2 above) showed a major peak at ∼26,295 daltons, which is ∼20 daltons greater than expected for the full-length, mature protein containing the His62 to 3 mH substitution (Supplementary Figure S7). The difference corresponds to the mass of one H_2_O molecule plus two hydrogen atoms, lost during the chromophore maturation through post-translational modification steps including cyclization/condensation and auto-oxidation/dehydrogenation (Sniegowski et al., 2005), respectively. This result indicates that 3 mH was correctly incorporated at position His62 by the PylRS(NMH)-tRNAPyl pair (Section 2.1.2) and that doing so decreased the efficiency of chromophore maturation. A close inspection of Supplementary Figure S7 reveals the presence of a smaller peak at ∼26,275 daltons that corresponds to the fully mature LEA-H62X, which helps to explain the low expression yields of this particular protein variant (Section 2.3). In contrast, electron density maps obtained from the diffraction of crystals of the same LEA-H62X protein sample (Supplementary Figure S6) present no evidence for the presence of immature chromophore intermediates. This finding implies that crystallization preferentially separated the fully mature protein from the immature form or, alternatively, the crystallization conditions may have facilitated chromophore maturation. We note that no significant chromophore maturation issues were observed for the other FP mutants with canonical amino acids, consistent with their high expression yields *versus* LEA-H62X. In fact, any immature chromophore, regardless of the relative population, would not be expected to interfere with the spectroscopic studies as it is effectively invisible. This point is corroborated by the conserved experimental conditions (e.g., excitation wavelength and power, resonance condition, probe region) and systematic spectral comparisons between all the LEA FP samples in this work (Sections 3.1–3.3).

**FIGURE 4:**
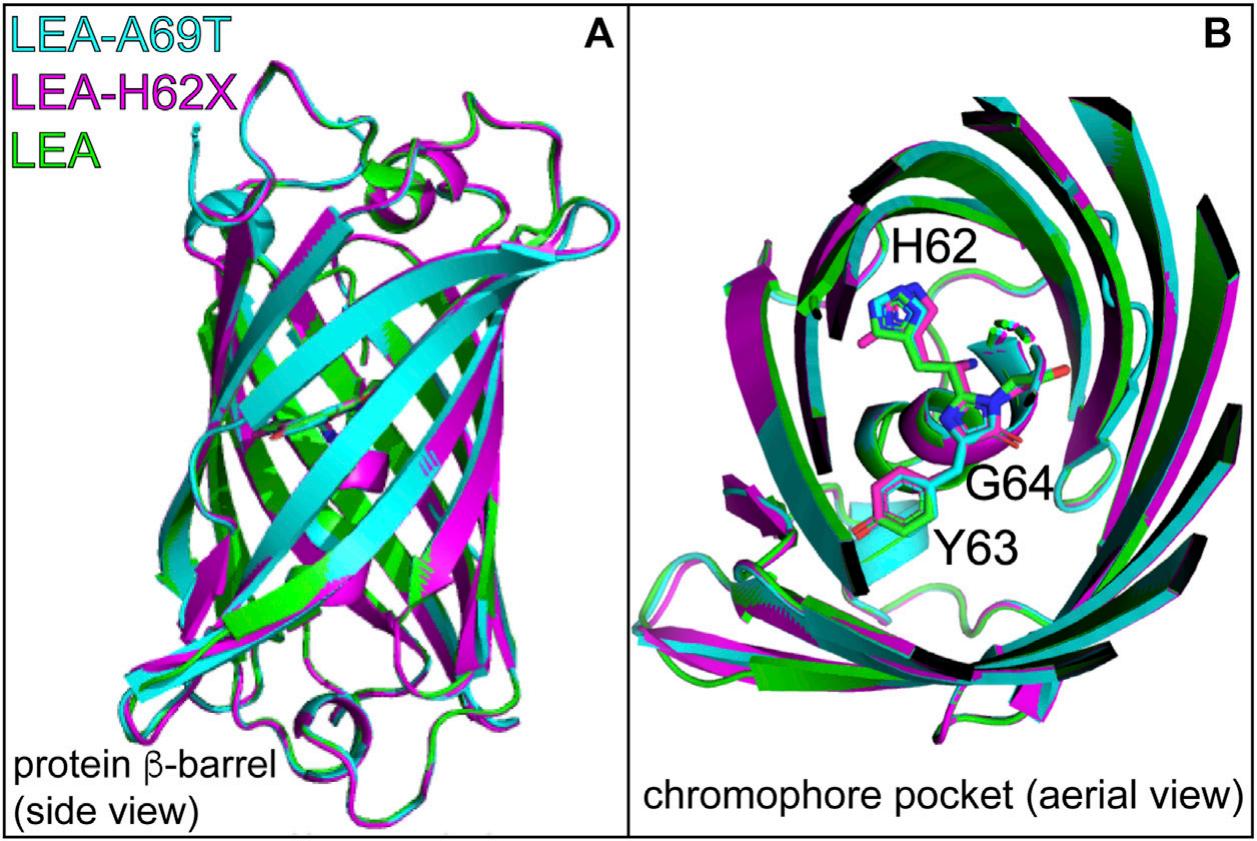
**(A)** Overlaid crystal structures of LEA (green, PDB ID: 4DXN), LEA-H62X (pink, PDB ID: 8UB6), and LEA-A69T (cyan, PDB ID: 8THS). **(B)** Top-down perspective of the LEA, LEA-H62X, and LEA-A69T crystal structures with the H62-Y63-G64 chromophore residing in the middle region of the β-barrel. The carbon backbone is shown in green (LEA), pink (LEA-H62X), and cyan (LEA-A69T). Residue abbreviations: H62, His62; Y63, Tyr63; G64, Gly64. Atomic colors: nitrogen, blue; oxygen, red. H atoms are omitted for clarity. See Supplementary Figure S6 for the electron density maps of the chromophore environments from crystal structures.

Notably, an overlay of the FP β-barrel and top-down perspective provides a glimpse of the HYG chromophores within the protein scaffold (Figures 4A, B), reinforcing the overall similarity among the three FPs. However, there are differences of the chromophore structure (including the ring coplanarity) and key residues surrounding the chromophore at both the P-ring and His62 ends, which are relevant towards understanding the drastic changes in the *on*→*off* photoswitching behavior.

**FIGURE 5:**
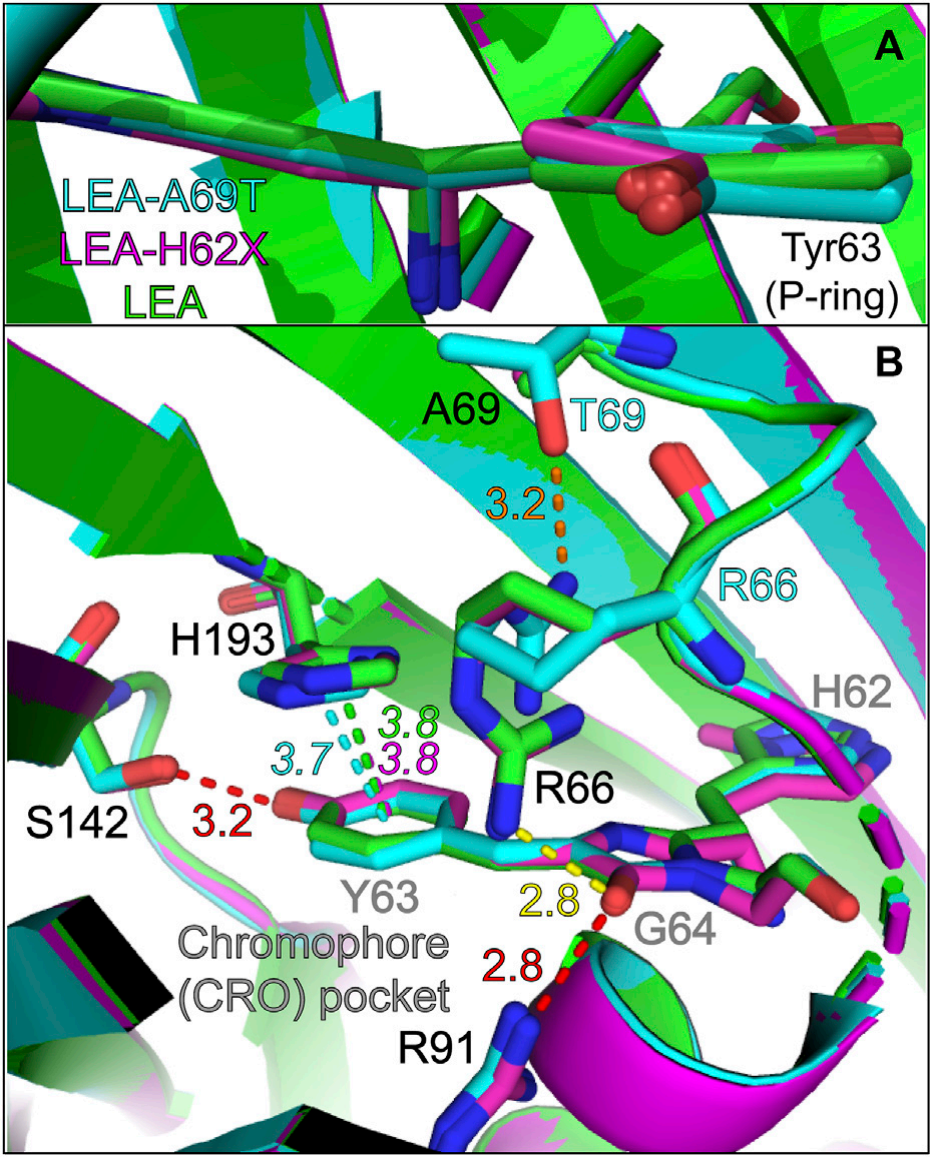
Overlaid crystal structures visualizing the **(A)** chromophore and **(B)** chromophore interactions with local environment residues near the phenolate (P-ring) of the chromophore. The chromophore and key residues in the chromophore pocket are shown for LEA (green), LEA-H62X (pink), and LEA-A69T (cyan). The green, pink, and cyan dashed lines represent the ring-to-ring distances between the P-ring/Y63 and H193 for LEA, LEA-H62X, and LEA-A69T, respectively. The dashed red lines indicate conserved polar interactions for all three pcFPs, the dashed yellow line indicates a polar interaction for LEA and LEA-H62X, and the dashed orange line highlights a unique polar interaction in LEA-A69T (between T69 and R66). The color-coded numbers near the dashed lines denote the respective distances in angstrom unit. For distinction in **(B)**, the chromophore residues are labeled in gray. Atomic colors: nitrogen, blue; oxygen, red. H atoms are omitted for clarity.

In Figure 5A, the P-ring orientation is displayed as an overlay of three chromophore structures. Relative to I-ring anchoring to the protein backbone, the P-ring of LEA (green) is more coplanar than LEA-A69T (cyan) and LEA-H62X (magenta). The P-to-I-ring dihedral angles (shown in Supplementary Figure S4 and Supplementary Table S7) decrease from LEA, LEA-H62X, to LEA-A69T. While many factors contribute to the ground-state absorption profile, the P-ring coplanarity with respect to the rest of the chromophore plays a considerable role (Zimmer, 2002; Altoè et al., 2005; Fang et al., 2009; Acharya et al., 2017; Tang and Fang, 2021). For example, the degree of planarity correlates with the absorption peak wavelength: LEA/LEA-A69T display the reddest/bluest absorption peaks with LEA-H62X lying in-between (Figure 1D). The LEA-A69T absorption is further influenced by the threonine mutation, as ALL-Q62H (∼179.5° bridge dihedrals) has a similar P-ring planarity to LEA yet also absorbs the bluest (in part) due to the presence of Thr69. However, the local environment surrounding the P-ring of LEA and LEA-H62X are essentially indistinguishable (Figure 5B); therefore, the blueshifted absorption of LEA-H62X likely stems from the slightly twisted structure that results in a reduced conjugation between the P- and I-rings, consistent with the blueshift according to the particle-in-a-box principle (McHale, 1999; Subach and Verkhusha, 2012; Acharya et al., 2017; Fang et al., 2019). Moreover, the differences between the GS-FSRS spectra of LEA and LEA-H62X, specifically those peaks comprised of the P-ring motions, are likely influenced by the planarity. Finally, the varying fluorescence quantum yield (FQY) values of GB among the FPs can be understood by the P-ring planarity of the bright green anionic chromophore. For example, LEA and ALL-Q62H have similar dihedral angles between the P- and I-rings and display similar FQY values of 0.81 and 0.79, respectively, upon excitation of the anionic chromophore (Kim et al., 2015). In contrast, with a slightly twisted P-ring conformation, LEA-A69T displays the lowest FQY of 0.73 upon 490 nm excitation. This is likely influenced by a reduced oscillator strength of the twisted chromophore in LEA-A69T. We note that the correlation between increasing FQY values and decreasing absorption peak wavelengths observed in many other FPs (Krueger et al., 2023a; Chen et al., 2023), including mCherry variants (Mukherjee et al., 2022), is not observed in the LEA and ALL-FPs. For instance, LEA possesses the highest GB* FQY while maintaining the reddest electronic features among the five FPs studied herein, supporting a photophysical interpretation beyond the energy-gap model (Englman and Jortner, 1970).

Regarding the drastic change to the photoswitching behavior under 505 nm irradiation, the pre-twisted P-ring of LEA-H62X may enable the substantially more efficient *on*→*off* photoswitching than LEA. If so, why does LEA-A69T not display any *on*→*off* photoswitching even though it has the most nonplanar structure? The lack of photoswitching in LEA-A69T is likely influenced by the π-stacked His193 that helps to maintain bright fluorescence of the anionic green chromophore. In LEA and LEA-H62X, His193 is effectively in an identical conformation and location with a ring-to-ring distance of 3.8 Å (Figure 5B). In LEA-A69T, His193 is slightly shifted horizontally resulting in a larger angle between the two rings yet maintaining a closer ring-to-ring distance of 3.7 Å (Supplementary Table S7). Moreover, the lack of photoswitching in ALL-Q62H with a ring-to-ring distance of 3.6 Å (Kim et al., 2015; Krueger et al., 2023b) and the greatly reduced photoswitching in LEA-A69T imply that steric hindrance can inhibit photoswitching to a great extent in addition to electrostatic effects via π-stacking interactions. We note that Thr69 mutation has been discussed greatly in relation to Kaede-like pcFPs (Henderson and Remington, 2005; Nienhaus et al., 2005; Remington et al., 2005; Kikuchi et al., 2008; Adam et al., 2009; Berardozzi et al., 2016; De Zitter et al., 2020; Krueger et al., 2023b) due to its inhibition of photoswitching and relevance towards primed conversion (Mohr et al., 2016; Mohr et al., 2017). In particular, Thr69 causes Arg66 to reorient and H-bond to the bulkier Thr69 in LEA-A69T and ALL-Q62H, instead of the I-ring C=O group in LEA and LEA-H62X via the reoriented Arg66 without an H-bond to the smaller Ala69 (Figure 5B). The correlation of the ring-to-ring distance from LEA/LEA-H62X > LEA-A69T > ALL-Q62H to the macroscopic photoswitching substantiates this structural parameter as a sensitive marker for photoswitching. A word of caution is that the photoswitching capability cannot simply be evaluated by the initial GB state alone; as proposed in a systematic study of mEos4b and IrisFP, the H-bonding network around the chromophore in the photoswitched *trans*-*off* state greatly influences the magnitude of photoswitching and lifetime of the *off* and *on* states (De Zitter et al., 2020).

**FIGURE 6:**
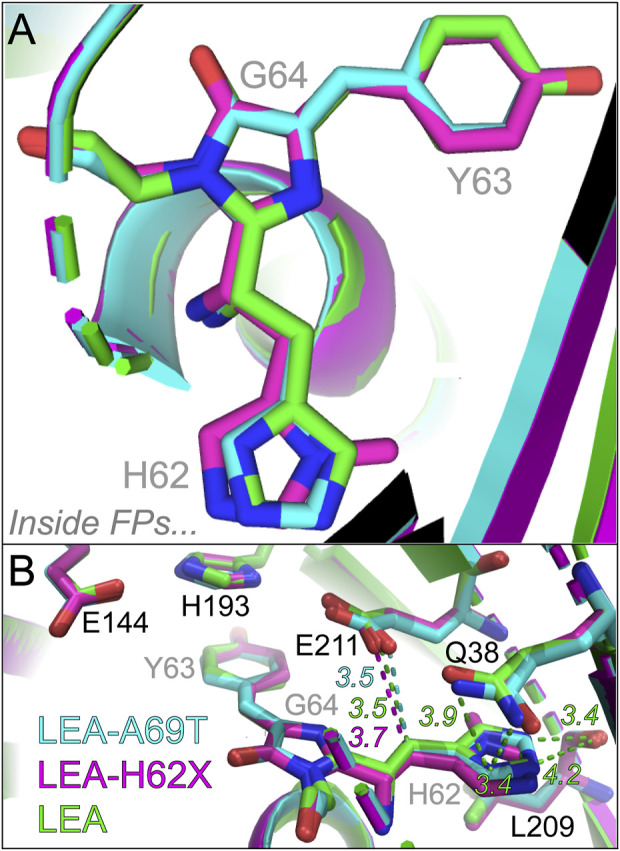
Overlaid crystal structures visualizing the histidine ring end of the chromophore. **(A)** Top-down perspective of the H62-Y63-G64 chromophore from crystal structures. **(B)** Chromophore interactions with local environment near the H62 end. The chromophore and key residues in the chromophore pocket are shown for LEA (green), LEA-H62X (pink), and LEA-A69T (cyan). The green, pink, and cyan dashed lines represent the distances between the carbon bridge and E211 for LEA, LEA-H62X, and LEA-A69T, respectively. Representative interactions and distances between the H62 ring and key local environment residues are shown as green dashed lines for LEA, the corresponding distances and interactions are shown for LEA-H62X and LEA-A69T in Supplementary Figure S8B, C, respectively. The color-coded numbers near the dashed lines denote the respective distances in angstrom unit. The chromophore residues are labeled in gray to contrast with several nearby residues labeled in black. Atomic colors: nitrogen, blue; oxygen, red. H atoms are omitted for clarity.

There are significantly more differences near the His62 end of the chromophore, manifested by the relative location of His62 via the overlaid chromophores (Figure 6A): the His62 ring in LEA-A69T and LEA-H62X shifts horizontally away from the P-ring compared to LEA. The extra methyl group causes the largest horizontal shift of the imidazole ring in LEA-H62X relative to LEA, with LEA-A69T lying between them. The photoconversion efficiency trend, LEA > LEA-A69T > LEA-H62X, correlates with the His62 horizontal shift magnitude that places LEA/LEA-H62X closest/farthest to/from the catalytically relevant Glu211 (Figure 6B) (Kim et al., 2013; Kim et al., 2015). The distance between Glu211 and the α -carbon comprising the bridge between the I-ring and His62 is 3.7 Å in LEA-H62X *versus* 3.5 Å in LEA (Supplementary Table S7). The Thr69 mutation in LEA-A69T causes Glu211 and Gln38 to shift from the identical residues in LEA-H62X and LEA (essentially superimposable), while the slightly shifted His62 maintains an α -carbon-to-Glu211 distance of 3.5 Å in LEA-A69T (Supplementary Figure S8C).

There are several other differences residing around the chromophore His62 end (Supplementary Figure S8). The crystal structures show that both His62 and the nearby Gln38 are rotated by ∼180° in LEA-H62X and LEA-A69T *versus* LEA, which changes the local environment at this end, including longer distances for polar interactions between the His62 nitrogen and Gln38 amide in LEA-H62X (4.5, 3.5 Å) and LEA-A69T (4.3, 3.6 Å) *versus* LEA (3.9, 3.4 Å). The reduced polar contacts around His62 in the pcFP mutants may allow the transient formation of a His62 rotamer that quenches the excited state and photoconversion, in accord with a non-photoconvertible Q38A mutant wherein Gln38 is replaced by a smaller residue (Ala38) to form such a rotamer (Kim et al., 2013). Meanwhile, the distances between Leu209 and His62 remain similar among three pcFPs, while the internal charge network of Glu211, His193, Glu144, and Arg66 (Supplementary Figure S9) also features similar distances and orientations in LEA and LEA-H62X. Though the P-ring local environment seems to be unaffected by the extra methyl group in LEA-H62X, it could change in the *trans* protonated state that affects the photoconversion efficiencies. Therefore, the largely invariant local environment supports the similar GS-FSRS spectra focusing on GB species and suggests that the different photoswitching behaviors arise from the final *trans* protonated state instead of the initial *cis* anionic state for the *on*→*off* photoswitching. In contrast, the distances within the internal charge network change drastically in LEA-A69T due to the Thr69-induced changes, wherein the distances between Glu144 and Arg66 increases from 4.4 to 6.2 Å (due to the reoriented Arg66) and the distance between Glu211 and His193 increases from 2.6–2.7 Å to 3.3 Å, while the Arg66 to Glu211 distance shrinks from 4.4–4.6 Å to 2.8 Å (Supplementary Figure S9C). Such a shortened distance between Arg66 and the catalytic base Glu211 (highly conserved among LEA FPs) and the resultant disruption to the internal charge network may underlie the significantly hindered photoconversion of LEA-A69T (Kim et al., 2015); however, more rationales need to be developed for the further reduction of photoconversion efficiency in LEA-H62X (Figures 1F; Supplementary Figure S9B).

Notably, the coplanarity of His62 ring remains similar for all three FPs (Supplementary Figure S4), which helps to address one major research question: does the extra methyl substituent cause His62 to form a rotamer akin to the LEA-Q38A mutant that inhibits photoconversion (Kim et al., 2013)? The crystal structures and overall similarity of GS-FSRS spectra indicate that such a rotamer does not form, at least for the equilibrated GB species. Notably, it is reasonable to evaluate crystal structures at the His62 end and analyze subtle differences among the LEA protein mutants (see above), while more prominent crystal structure/local environment changes are expected near the P-ring upon comparing the *cis* (*on* state) *versus trans* (*off* state) structures (Bourgeois and Adam, 2012; Nienhaus and Nienhaus, 2014; Tang and Fang, 2022) which will benefit from future crystallographic studies about the green *off* state. However, it is possible that His62 begins as a mostly coplanar structure in the ground state of GB, but transiently twists in the excited state. To address this question, we performed fs-TA experiments to examine initial dynamics of the anionic green chromophore in contrasting pcFPs upon photoexcitation.

### 3.3 Tracking excited-state chromophore dynamics with ultrafast electronic and vibrational spectroscopies: ring-twisting motions dictate the energy dissipation pathways

**FIGURE 7:**
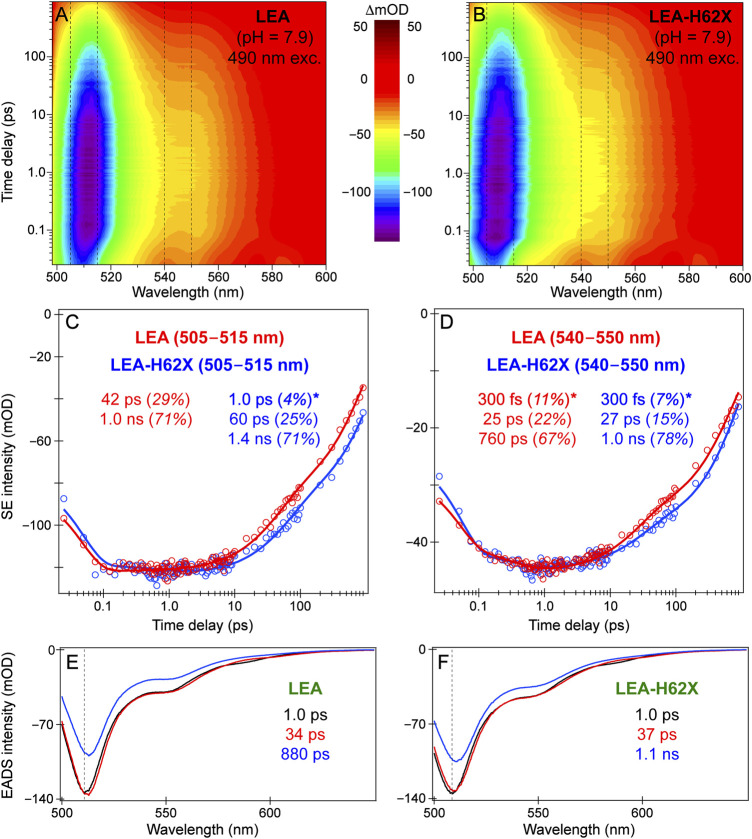
Femtosecond transient absorption (fs-TA) of LEA and LEA-H62X in pH 7.9 buffer upon 490 nm excitation. The semilogarithmic contour plots of fs-TA spectra of **(A)** LEA and **(B)** LEA-H62X. The color-coded intensity levels are shown in milli-optical density (mOD) units between the panels. The probe-dependent stimulated emission (SE) peak intensity dynamics of LEA (red) and LEA-H62X (blue) with integration regions from **(C)** 505–515 and **(D)** 540–550 nm. The data points (hollow circles) are overlaid with the least-squares multiexponential fits (solid curves). The time constants and weighted amplitude percentages are listed, the asterisk denotes a rise time constant. The evolution-associated difference spectra (EADS) in mOD units are plotted for **(E)** LEA and **(F)** LEA-H62X. The retrieved color-coded lifetimes from global analysis are listed, the vertical dashed line indicates the initial SE peak position.

For LEA and LEA-H62X upon 490 nm excitation, we present the fs-TA contour plots, probe-dependent dynamics, and global analysis results (Figure 7) to retrieve the onset/primary events during *on*→*off* photoswitching from GB species. The fs-TA spectra (Figures 7A, B) up to 900 ps reveal a strong stimulated emission (SE) peak at ∼508 and 510 nm for LEA-H62X and LEA, close to their fluorescence peaks (Supplementary Figure S1B). In steady-state absorption and fluorescence spectra, LEA-H62X is blueshifted from LEA by ∼2–3 nm which remains consistent in the ultrafast spectra. A clear shoulder is present in the SE band to the red side of the peak maximum for both LEA and LEA-H62X from ∼540 to 560 nm, typical for vibronic coupling in FPs (Tachibana et al., 2018; Fang et al., 2019).

Reminiscent of the GS-FSRS spectral similarity, the fs-TA spectra of LEA and LEA-H62X display common features involving a prompt SE peak rise and then decay after photoexcitation (Figures 7C, D). A minor ∼1 ps rise of the SE peak was observed in LEA-H62X, attributed to solvation by internal water molecules within the protein scaffold (Fang and Tang, 2020; Wang et al., 2020). Such a rise has been observed in LEA experiments (Krueger et al., 2023b), which represents a minor component that is not especially relevant for photoswitching. Next, the 1.0–1.4 ns component tracks the apparent fluorescence lifetime (τ_FL_), an average of radiative and nonradiative processes, and is a good measure of the FQY. The longer lifetime of LEA-H62X than LEA suggests that the noncanonical incorporation of 3 mH-His62 may increase the brightness of the green anionic chromophore; however, a similar τ_FL_ was observed from previous fs-TA experiments on LEA (∼1.3 ns). There could be some variations of kinetic components between experiments; however, we rely on comparative experiments under identical experimental conditions for a reliable comparison between two similar pcFPs.

The most interesting fs-TA result is the intermediate component: 60 ps (25%) for LEA-H62X and 42 ps (29%) for LEA. This component was assigned to a chromophore twist by comparing fs-TA spectra of LEA, LEA-A69T, ALL-Q62H, and ALL-GFP upon 490 nm excitation (Krueger et al., 2023b). The primary chromophore twisting coordinate likely involves the P-ring, although the kinetic TA component does not track the full *cis*→*trans* isomerization expected to occur on longer timescales. The intermediate component likely also involves a holistic twist of the entire chromophore including His62. Our previous fs-TA data on the non-photoswitchable ALL-GFP/ALL-Q62H revealed that Gln62/His62 undergoes conformational motions to nonradiatively dissipate the photoexcitation energy with a faster component of ∼15–30 ps (Krueger et al., 2023b). In contrast, the intermediate component of LEA-A69T is similar to LEA (40–60 ps) but occurs with a drastically reduced weight *versus* LEA, which indicates that the chromophore twist in LEA-A69T is significantly subdued by the threonine mutation that is correlated to the greatly reduced photoswitching of this mutant pcFP (Kim et al., 2015).

Over several fs-TA experiments on LEA upon 490 nm excitation (Krueger et al., 2023b), a 40–60 ps component was retrieved for the main SE peak of LEA that is similar to the 60 ps decay of LEA-H62X, although the decay dynamics of LEA-H62X are consistently longer than LEA (Supplementary Figure S10). This is counterintuitive for LEA-H62X: the prominent and rapid *on*→*off* photoswitching of LEA-H62X *versus* LEA (Figure 1E) should be reflected by a faster intermediate decay and/or an intermediate decay with a larger weight. However, the intermediate decay of LEA-H62X may occur with a reduced weight relative to LEA (Figures 7C, D) because of a pre-twisted P-ring in the LEA-H62X crystal structure Figure 5; Supplementary Figure S4). The equilibrated, twisted structure of LEA-H62X likely possesses a reduced oscillator strength for the downward transition (Kumpulainen et al., 2017; Fang et al., 2019) upon photoexcitation relative to LEA; thus, any further twist of the LEA-H62X chromophore in the excited state will result in a reduced change to the oscillator strength compared to LEA. In other words, LEA enters the excited state with a nearly coplanar P-ring conformation where any twist from this starting point can result in a more noticeable reduction in the GB* SE peak intensity than LEA-H62X.

The overall similarity between the fs-TA spectra of LEA-H62X and LEA upon 490 nm excitation implies that the onset of photoswitching remains similar; thus, differences in the photoswitching behavior likely occur on longer timescales, which is supported by literature (Laptenok et al., 2018; Woodhouse et al., 2020; Tang and Fang, 2022) and the overall similar “equilibrated” GS-FSRS spectra and crystal structures obtained for these two pcFPs. We reckon that different photoswitching behaviors can arise from different local environments experienced by the *trans*-like chromophore after the *cis*→*trans* isomerization, and the protonated *trans* chromophore needs to be investigated by a multitude of techniques including GS/ES-FSRS, fs-TA, X-ray crystallography, and MD simulations. Besides future work, there is one current observation supporting this idea: the photoswitched *off* state of LEA-H62X (GA’) spontaneously recovers the initial resting state (GB) at thermal equilibrium in the dark, while the GA’ species in LEA remains stable in the photoswitched *off* state at thermal equilibrium (Figure 1E). The highly dynamic GA’ state in LEA-H62X resembles comparative investigation of mEos4b and IrisFP (De Zitter et al., 2020), which proposed spontaneous recovery of the photoswitched *off* state to be largely controlled by the H-bonding network around the protonated *trans* chromophore: the more H-bonds the protonated *trans* chromophore experiences, the lower energy barrier for isomerization in both excited and ground states, thus the more likely it is to spontaneously recover to the original resting state (i.e., LEA-H62X in this work, reminiscent of IrisFP). We note that the differences in H-bonding patterns between the *cis* and *trans* states matter. Further evidence that the relevant differences between LEA and LEA-H62X arise from the protonated state is a dramatic reduction in photoconversion efficiency of LEA-H62X *versus* LEA, since the protonated chromophore is the sole photoconvertible species in the LEA pcFPs (Kim et al., 2013; Kim et al., 2015). As analyzed above, the protonated chromophore is stabilized by more H-bonds in the *off* state and inhibits photoconversion of LEA-H62X (also displaying a redshifted absorption peak *versus* LEA in Figure 1E), reminiscent of the redshifted absorption peak of GA’ (with more H-bonds, see Section 3.1 above) *versus* GA in the *off* state of LEA.

A substantial difference is present in the vibronically-coupled shoulder peak of LEA and LEA-H62X (Figure 7D). Overall, the fs-TA dynamics are even more similar in this region: both pcFPs reveal a sub-ps (300 fs) initial rise likely due to solvation and a minor redshift of the SE peak (i.e., toward the shoulder band). The apparent fluorescence lifetime is slightly faster, but the trend where LEA possesses a faster $\tau_{FL}$ than LEA-H62X still holds. The largest change to the dynamics occurs in the intermediate component, which is shortened from ∼40 to 60 ps to 25–27 ps. Similar to the main SE peak, the amplitude weight of this intermediate component is reduced in LEA-H62X, supporting the pre-twisted P-ring interpretation with a reduced transition oscillator strength. We note that the faster intermediate decay in this spectral region has a similar time constant to that in ALL-Q62H (29 ps) and ALL-GFP (16 ps), attributed to conformational motions of H62/Q62 (Krueger et al., 2023b). It is reasonable that similar motions may occur in LEA and LEA-H62X, which affect the degree of vibronic coupling to result in an apparent decay of this shoulder peak. This mechanism means that the His62 far-imidazole motions may better couple to the lower-energy, weak portion of SE (transient) and fluorescence (equilibrium) band, consistent with a higher tendency to result in a nonradiative decay with the aforementioned His62 rotamer to effectively change the His62 ring location/conformation (Kim et al., 2013; Krueger et al., 2020).

The evolution-associated difference spectra (EADS) with a sequential kinetic model retrieved from global analysis (Berera et al., 2009; Snellenburg et al., 2012) are presented in Figures 7E, F. The lifetimes remain similar to the probe-dependent fits, where the second (34–37 ps) and third (0.88–1.1 ns) lifetimes are averages of the main SE peak and shoulder band. LEA displays a slight rise of the SE peak on the ∼1 ps solvation timescale (black→red) but LEA-H62X shows a slight decay. This minor component tracks the initial chromophore dynamics out of the Franck-Condon/FC region (Wang et al., 2020; Tang et al., 2022), since both EADS reveal a slight (∼2 nm) redshift of the SE band on the 1 ps timescale, indicative of solvation-induced vibrational cooling that slightly relaxes the S_1_ state. A minor redshift (∼1 nm) of the SE band continues on the intermediate timescale (red→blue), indicative of further conformational relaxation that likely involves a chromophore twist. Why does LEA-H62X display a slightly longer intermediate decay than LEA? The bulkiness introduced by the extra methyl group can cause steric effects that slow the conformational motions to a degree, especially near His62. The electrostatic interactions may also be affected by the ncAA incorporation, but a methyl substituent is not expected to incur a large electrostatic change, supported by the overall similar GS-FSRS spectra (Figure 3). Therefore, the retrieved time constants tracking conformational motions of the chromophore (∼25–60 ps) are reflective of the entire chromophore and not just the P-ring. Otherwise, one may expect that the pre-twisted P-ring alone should experience a faster decay and reduced weight in LEA-H62X because it starts from a twisted geometry for the *on*→*off* photoswitching; this is not the case here.

**FIGURE 8:**
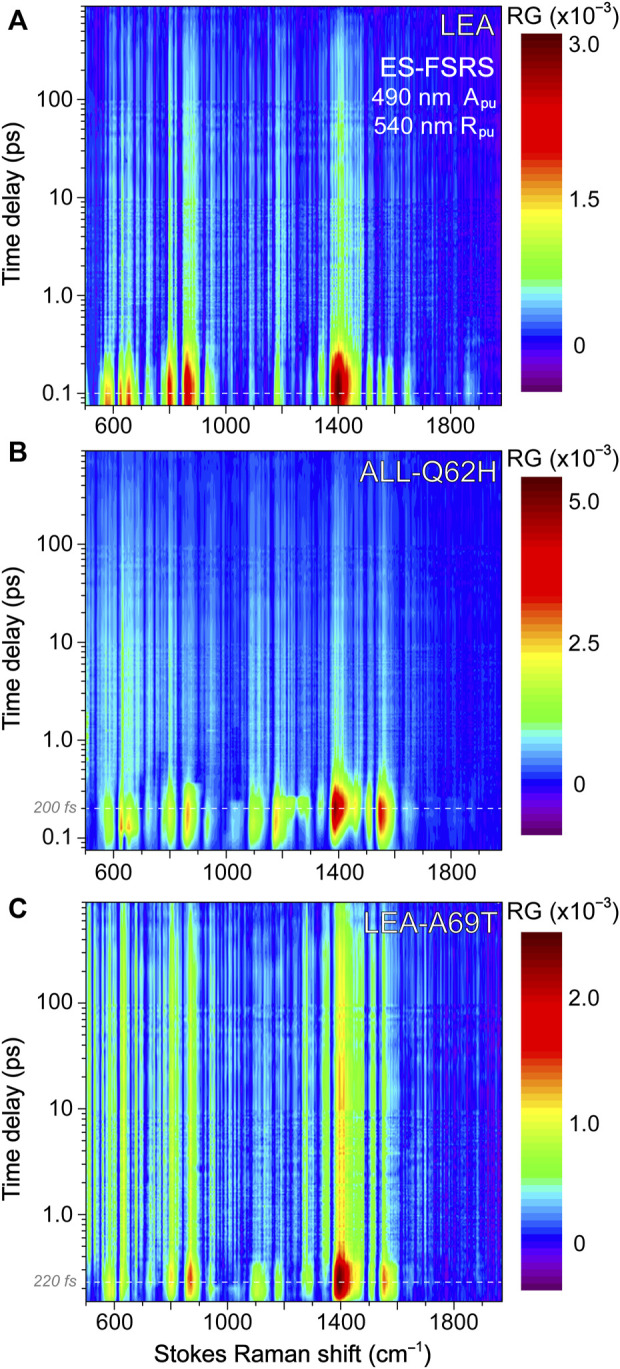
Two-dimensional contour plots of the excited state (ES)-FSRS spectra of **(A)** LEA, **(B)** ALL-Q62H, and **(C)** LEA-A69T in pH 7.9 buffers. The time-resolved spectra were collected with actinic pump (A_pu_) and Raman pump (R_pu_) wavelengths of 490 and 540 nm, respectively, with an average Raman pump power of ∼4 mW. The Raman probe was redder than R_pu_, hence the spectra are all on the Stokes side. The color-coded intensity levels are shown to the right of each panel as Raman gain (RG, ×10^–3^). The apparent experimental time zero (ca. 100–220 fs) on different experimental days is denoted by the white horizontal dashed line in each panel.

Inspecting the fs-TA dynamics in this spectral region is especially relevant for the ES-FSRS measurements of LEA and LEA-A69T because the Raman pump was tuned to 540 nm near the shoulder band. ES-FSRS is a powerful experimental technique in revealing the excited-state evolution of Raman peaks, which expose transient structural changes with site specificity via vibrational motions and enhance spectral signatures of targeted species by tuning the Raman pump to a specific wavelength of interest (Fang et al., 2009; Dietze and Mathies, 2016; Fang et al., 2018). The contour plots of ES-FSRS spectra for LEA, ALL-Q62H, and LEA-A69T (Figure 8) delineate the excited-state vibrational evolution of the GB* population with actinic and Raman pumps of 490 and 540 nm, which excite the GB ground-state population and are resonant with the GB* SE feature, respectively. With a detection window spanning ∼500–2000 cm^−1^, the spectral similarities from three pcFPs are apparent.

**FIGURE 9:**
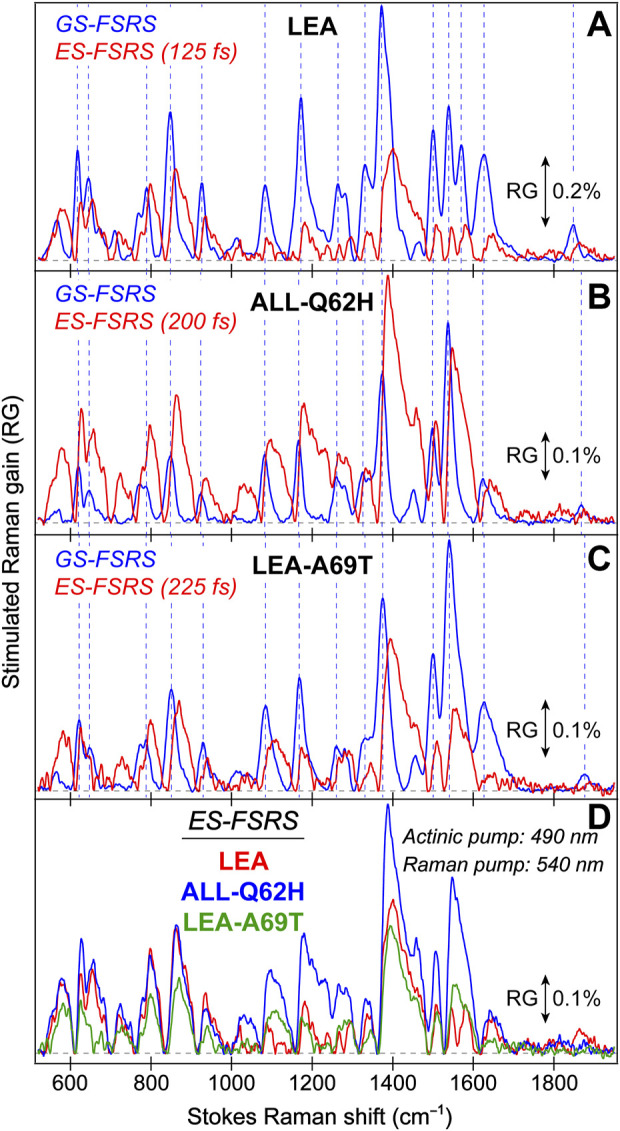
A comparison of the ground state (GS)-FSRS and excited state (ES)-FSRS spectra of **(A)** LEA, **(B)** ALL-Q62H, and **(C)** LEA-A69T. In **(A–C)**, the GS spectra (blue traces) were collected with a 540 nm Raman pump, and the ES spectra (red traces) were collected with actinic and Raman pump wavelengths of 490 and 540 nm, respectively, with a redder Raman probe on the Stokes side. The blue dashed vertical lines highlight the differences in GS peak frequencies between the FPs, as well as the shifted ES peaks from their GS counterparts. The ES-FSRS spectra were selected at time points near time zero where the peaks reach maximum intensity; these time points shift slightly between the FPs based on the experimental conditions. In **(D)**, the unnormalized ES-FSRS spectra from **(A–C)** were plotted to compare LEA (red), ALL-Q62H (blue), and LEA-A69T (green). In **(A–D)**, the average Raman pump power was set to ∼4 mW to improve the signal-to-noise ratio, and the double-sided arrows represent the stimulated Raman gain (RG) magnitudes of 0.2% or 0.1%.

Focusing on the Franck-Condon region, the GS and ES-FSRS spectra are compared (Figure 9) with representative ES-FSRS spectra selected around the peak maximum (100–200 fs) of a pronounced Raman marker band (∼1400 cm^−1^). The spectra in Figures 8, 9 were collected with a relatively high Raman pump power of 4 mW, resulting in clear ES-FSRS peaks that allow for easy comparison. In general, the ES Raman peaks are blueshifted from their GS counterparts for all three FPs. As discussed in Figure 3, the most intense GS-FSRS peak for ALL-Q62H and LEA-A69T is ∼1540 cm^−1^, while the most intense peak is ∼1370 cm^−1^ for LEA due to Ala69 and the reoriented Arg66 (Figure 5B). A comparison of the ES-FSRS spectra (Figure 9D) highlights similar GB* vibrational signatures near the FC regions of three pcFPs with varying chromophore conformations and local environments. This pattern is reminiscent of fs-TA spectra comparing the electronic dynamics of LEA *versus* LEA-H62X (Figure 7), implying that different photoswitching behaviors arise on longer timescales and may be influenced more by the final *trans*-like photoswitched *off* state than the initial *cis*-like *on* state.

There are several notable spectral differences, especially when comparing the photoswitchable LEA to the nonphotoswitchable ALL-Q62H and LEA-A69T. Similar to the GS-FSRS spectra (Figure 3B), the low-frequency ES-FSRS peaks of LEA are generally stronger than the other two FPs (Figure 9D) due to a more spacious and flexible chromophore pocket in LEA resulting in a polarizability increase, consistent with the crystal structures (Figures 5, 6; Supplementary Figure S6) (Kim et al., 2015; Krueger et al., 2023b). These low-frequency vibrational peaks involve relatively delocalized motions throughout the chromophore, so they are a good measure of free space experienced by a photosensing “active” site. In particular, several low-frequency peaks involve skeletal motions centered on the P-ring, which resides in a more spacious pocket in LEA (Figure 5B). Additionally, the 540 nm Raman pump and broadband probe are slightly more resonant in LEA due to its redshifted SE band *versus* the other two FPs, which could contribute to stronger ES-FSRS peaks of LEA; however, this effect is likely minor due to the observed peak intensities (Figure 9D) not being uniformly larger for LEA than the other FPs.

**FIGURE 10:**
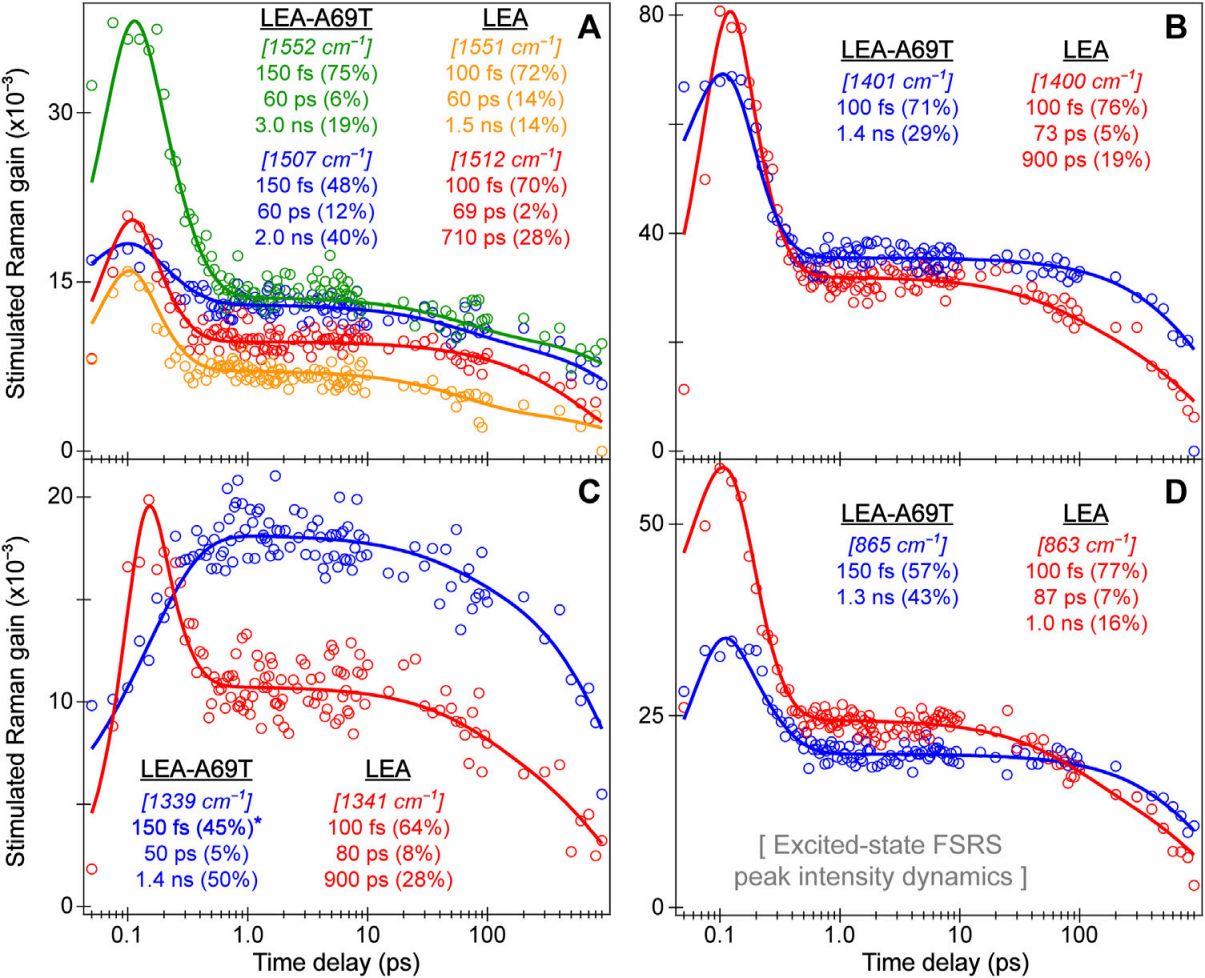
Intensity dynamics from ES-FSRS measurements on LEA and LEA-A69T collected with a 490 nm actinic pump and a 540 nm Raman pump, with an average Raman pump power of ∼2.5 mW. Intensity dynamics from ES-FSRS peaks at **(A)** ∼1550 and 1510 cm^−1^ from LEA-A69T (green, blue) and LEA (orange, red), **(B)** 1400 cm^−1^, **(C)** 1340 cm^−1^, and **(D)** 865 cm^−1^ are plotted for direct comparison. Data points are shown as hollow circles with the least-squares exponential fits overlaid as solid curves. The color-coded time constants and weighted amplitudes are listed accordingly in the insets. The asterisk in panel **(C)** denotes a rise time constant. The lower Raman pump power reduces the unwanted excited-state depletion effect as shown in Supplementary Figure S12.

In stark contrast, the high-frequency peaks from ∼1500 to 1600 cm^−1^ display a greatly reduced intensity for LEA *versus* the other two FPs (Figure 9D). This trend was also observed in the GS-FSRS spectra (Figure 3B) and attributed to Thr69/Ala69-induced changes to the local environment, especially around I-ring. These peaks primarily involve C=N and C=O stretching motions on the I-ring, which are reduced in LEA because Arg66 is H-bonded to the I-ring inhibiting the extent of its vibrations. This observation discredits the resonance conditions playing a dominant role in ES-FSRS peak intensities, because the LEA SE peak is more resonant with the Raman pump-probe pair. In sum, these spectral findings reveal a more spacious chromophore pocket in LEA, enabling pronounced photoswitching and photoconversion (Krueger et al., 2020; Krueger et al., 2023b) while the I-ring is more confined as an anchor. For comparison, the more spacious local environment around the I-ring in ALL-Q62H and LEA-A69T permits its flexibility to enhance the intrinsic nonradiative decay (Litvinenko et al., 2003; Martin et al., 2004; Altoè et al., 2005; Taylor et al., 2019) which quenches potential photochromic abilities, consistent with some pertinent I-ring motions showing an intermediate “twisting” component in LEA-A69T (Figure 10A) but not for other motions (Figures 10B, D). Moreover, the peak doublet from ∼600 to 700 cm^−1^ in GS-FSRS spectra was discussed (Krueger et al., 2020; Krueger et al., 2023b) for the relatively stronger higher-frequency peak in LEA as a key conduit mode involving His62 motions as well as the bridge between I-ring and His62. Interestingly, this pattern is conserved in ES-FSRS spectra: the higher-frequency peak is slightly stronger than the lower-frequency peak in LEA (Figure 9D). In contrast, the ∼650 cm^−1^ mode is weaker in ALL-Q62H and LEA-A69T, reinforcing the assignment of this conduit mode as a vibrational marker for photochromic pcFPs.

There are several other interesting observations that could motivate future studies. The ∼1850 cm^−1^ mode is significantly more intense for LEA than the other two FPs, both in the GS and ES, and may involve the I-ring C=O stretch (see Section 3.2 above). Similarly, LEA has a stronger ∼1625 cm^−1^ mode (attributed to P-ring C=O stretch in a deprotonated chromophore) than LEA-A69T with a twisted P-ring. Notably, the ∼1550 cm^−1^ peak of LEA is greatly diminished *versus* the other two FPs with the Thr69-induced changes to the local environment, so it can be a marker band for nonphotoswitchable FPs. The pertinent mode assignment of C=N and C=O stretch on the I-ring rationalizes its reduced ES intensity due to an extra H-bond between Arg66 and the I-ring in LEA (Krueger et al., 2023b). In addition, there is a pronounced profile change of the 1370 cm^−1^ GS peak that shifts to ∼1400 cm^−1^ in the ES, wherein a clear GS blue shoulder becomes the dominant ES peak with a red shoulder at the GS peak location (Figures 9A–C), as well as the intensity pattern change at ∼1330 cm^−1^ (stronger in the GS) and ∼1470 cm^−1^ (stronger in the ES). Interestingly, this vibrational mode frequency matches the energy gap between the main SE peak and its red shoulder peak (Figures 7E, F) at ∼1400 cm^−1^ (e.g., 513 and 553 nm in LEA), which is also the strongest peak upon photoexcitation (Figure 8). The significant decay of this mode intensity on the ∼100 fs timescale exhibits a clear dependence on the R_pu_ power (Supplementary Figures S11, S12A, B), lending further support to its pronounced vibronic activity through FC region and high sensitivity to the R_pu_-induced dumping effect (see below). This Raman marker band with bridge H-rocking and I-ring in-plane deformation (see Supplementary Table S2 for LEA) thus tracks coupling between the electronic and vibrational degrees of freedom (Fang et al., 2019) as well as interactions between the chromophore and its local environment (Lin et al., 2019; Romei et al., 2020; Chen et al., 2023).

The ES-FSRS mode intensity dynamics are compared for LEA and LEA-A69T (Figure 10) to gain deeper insights into the excited-state chromophore and the effects of Thr69. Complementary to fs-TA that monitors electronic dynamics, ES-FSRS affords the structural specificity during navigation of the excited population through the potential energy surface. Using the localized and delocalized vibrations as sensitive molecular probes, one can determine which atomic motions and regions dictate the excited-state dynamics and energy dissipation pathways. The ES-FSRS data in Figure 10 were collected with a reduced R_pu_ power of 2.5 mW to track vibrational evolution with increased sensitivity, the respective ES-FSRS contour plots are shown for LEA and LEA-A69T (Supplementary Figure S11). The excited-state Raman peak dynamics with the 2.5 and 4 mW R_pu_ powers are compared (Supplementary Figure S12) to reveal that the peak intensity decay is more prominent on the sub-ps timescale with a higher R_pu_ power, so the intrinsic excited-state dynamics are better viewed with a lower R_pu_ power. Since R_pu_ is pre-resonant with the SE peak, this result indicates that R_pu_ likely dumps some of the excited-state chromophore population back to the ground state (Weigel et al., 2011; Dietze and Mathies, 2016; Fang et al., 2019).

The intensity dynamics of the ∼1550 and 1510 cm^−1^ modes are compared for LEA and LEA-A69T (Figure 10A). These excited-state Raman modes consist of similar vibrational motions of the chromophore, yet they exhibit kinetic differences. The 1550 cm^−1^ mode involves vibrations throughout the P- and I-rings including the I-ring C=N and C=O stretch, bridge C=C stretch, and P-ring C=C and C=O stretch; while the lower-frequency 1510 cm^−1^ mode primarily involves P-ring asymmetric C=C stretch and I-ring C=N stretch (Supplementary Tables S2, S3). The transient evolution of these two modes is thus intriguing because the dynamics are flipped between the two pcFPs. In LEA, the higher-frequency peak shows a larger intermediate decay than the same peak in LEA-A69T and the lower-frequency peak in LEA. In LEA-A69T, the lower-frequency peak shows a larger intermediate decay (on the twisting timescale) than the same peak in LEA and the higher-frequency peak in LEA-A69T. In essence, the intermediate decay dynamics attributed to the chromophore twist is reversed in two pcFPs.

To delve deeper into vibrational dynamics, we note that in LEA-A69T around time zero, the 1552 cm^−1^ mode is about twice as strong as the 1507 cm^−1^ mode, as well as both the 1551 and 1512 cm^−1^ modes in LEA. This is the most intense peak in the GS-FSRS spectrum of LEA-A69T (Figure 3B) and may be highly active near the FC region, supported by its prominent sub-ps decay (150 fs, 75%) *versus* the 1507 cm^−1^ mode (150 fs, 48% weight). Among all the ES-FSRS modes of LEA-A69T, the ∼1550 cm^−1^ mode exhibits the largest dependence on the ∼100–150 fs decay, in accord with its I-ring motion assignment (Supplementary Table S3) and increased flexibility around the anchoring I-ring due to Thr69 H-bonding to Arg66 (Figure 5B) (Kim et al., 2015; Krueger et al., 2023b). In fact, by ∼1 ps, the mode intensity dynamics mostly overlap; therefore, relying solely on the weighted amplitudes of the twisting components can be misleading as the decay on longer timescales is quite similar. The vibronic coupling magnitude of this peak in LEA has been shown to be reduced *versus* the other FPs, which is likely induced by the Ala69 mutation besides other cascading effects (Chen et al., 2023). On the other hand, the 1551 and 1512 cm^−1^ modes in LEA show a similar sub-ps decay magnitude and are of near equal intensity around time zero. In this case, the difference in the twisting-induced decay magnitude is more apparent, with the higher-frequency peak displaying a more pronounced decay on the ∼60 ps timescale. The mode assignment helps to rationalize this finding since the higher-frequency mode involves the bridge C=C stretch between P- and I-rings, which should be more sensitive to the chromophore ultrafast twisting process that primarily involves the P-ring twist than the lower-frequency mode (Supplementary Table S2). In contrast, the “flipped” dynamics in LEA-A69T (i.e., the higher-frequency mode is less sensitive to the twisting component) could be due to less P-ring twist but more I-ring twist, supported by the observed change for the ∼1550/1510 cm^−1^ mode frequency dynamics in LEA-A69T *versus* LEA (Supplementary Figure S13A).

The intensity dynamics of the strongest ES-FSRS mode at ∼1400 cm^−1^ allow another key comparison between LEA and LEA-A69T (Figure 10B), besides the pronounced ∼100 fs decay (71%–76% weight) that substantiates its strong vibronic coupling to photoexcitation (see above). Notably, LEA manifests an intermediate 73 ps decay component that is absent in LEA-A69T, reminiscent of the fs-TA difference between LEA and LEA-A69T (i.e., LEA-A69T shows a greatly diminished twisting component *versus* LEA). These results directly tie to the much reduced macroscopic photoswitching behavior of LEA-A69T *versus* LEA, further supported by the excitation-dependent changes to fs-TA dynamics (Krueger et al., 2023b). This conspicuous larger decay on the intermediate timescale confirms that the LEA chromophore is in a more spacious local environment, enabling characteristic twists that are essentially missing in LEA-A69T (Kim et al., 2015). Moreover, the ∼1400 cm^−1^ mode assignment of H-rocking on the carbon bridge connecting I-ring and His62 plus I-ring deformation supports this twisting component to involve the holistic chromophore twists across the chromophore framework, previously shown from fs-TA analysis of ALL-GFP and ALL-Q62H (Krueger et al., 2023b). Similarly, the ∼865 cm^−1^ mode intensity dynamics (Figure 10D) assigned to P-ring breathing, P-to-I-ring bridge CCC bending, and I-ring deformation paints a similar picture: LEA-A69T does not decay on the intermediate twisting timescale because the P-ring is more confined via electrostatic and steric interactions with an adjacent His193 residue (Krueger et al., 2023b), whereas LEA shows a 87 ps twisting time constant from a stronger mode that is a staple for the low-frequency modes in LEA (Figure 3B; Figure 9D). The much greater sub-ps decay of this mode for LEA (100 fs, 77%) than LEA-A69T (150 fs, 57%) indicates a larger displacement out of the FC region in LEA, likely involving the intramolecular charge transfer (ICT) from P- to I-ring of the chromophore (Altoè et al., 2005; Tonge and Meech, 2009; Taylor et al., 2019). Notably, this ICT should be more prominent in LEA due to the extra H-bond between Arg66 and the I-ring C=O group that effectively stabilizes the negative charge on I-ring *versus* LEA-A69T (Figure 5B). In addition, the frequency dynamics of several ES-FSRS peaks (GB*) can be compared and analyzed (Supplementary Figure S13) to further elucidate transient differences between LEA and the single-site mutant. Interestingly, the aforementioned 1400 and 865 cm^−1^ modes exhibit dominant frequency blueshift time constants of 100–150 fs without other components (Supplementary Figure S13C, E), corroborating their pronounced, major activity guiding the excited-state chromophore out of the FC region via efficient vibrational cooling. In contrast, some other Raman modes can better track the chromophore twisting on the tens of ps timescale (e.g., ∼1510 and 1340 cm^−1^ modes for LEA in Supplementary Figure S13B, D), consistent with their mode assignments of P-ring C=C stretch and methine bridge H-rocking motions (Supplementary Table S2).

The most interesting and informative ES-FSRS dynamics are presented by the ∼1340 cm^−1^ mode of LEA-A69T which manifests a delayed rise with a ∼150 fs time constant (Figure 10C). In contrast to the other ES-FSRS modes, the 1340 cm^−1^ mode is the only observed vibrational peak that rises appreciably on the 100–150 fs timescale instead of a dominant decay. This mode assignment to the P-to-I-ring bridge H-rocking, along with P-ring H-rocking and I-ring deformation, was a major focus in a previous report (Krueger et al., 2020). In brief, the corresponding GS-FSRS peak at ∼1332 cm^−1^ was shown to rise dramatically during the photoconversion of LEA under 400 nm LEDs via time-resolved GS-FSRS. The mode intensity rise was attributed to a twisted P-ring structure that accumulates over time to form a permanently trapped deprotonated green chromophore (i.e., GB’) adopting a near perpendicular P-ring orientation with respect to the rest of the chromophore, which can no longer photoswitch or replenish the protonated population. In LEA, this GB’ population forms during *off*→*on* photoswitching under 400 nm illumination (Krueger et al., 2020). In essence, during the *trans*→*cis* isomerization for photoswitching, a distorted and permanently trapped chromophore subpopulation could form by specific interactions with the dynamic local environment favoring the *on* state.

In LEA-A69T, this specific mode rises on the sub-ps timescale near FC region under 490 nm excitation, instead of on the minutes timescale under 400 nm irradiation like that observed in LEA. Therefore, the LEA-A69T GB chromophore may be more prone to reach a GB’-like conformation on ultrafast timescales in the excited state (GB’*) as tracked by this methine-bridge-sensitive vibrational probe (Tang et al., 2018; Fang and Tang, 2020). This scenario explains why the GB population in LEA-A69T cannot photoswitch and why there is a greatly reduced twisting component in its fs-TA and ES-FSRS data: a GB’* population forms on ultrafast timescales and becomes trapped so it can no longer twist, isomerize, or photoswitch effectively. Notably, this result is also supported by a previous study showing slight positive photoswitching (i.e., the population of the species under irradiation increases) of LEA-A69T under 490 nm excitation (Krueger et al., 2023b). Furthermore, the pre-twisted P-ring observed in the crystal structure (Figure 5) likely contributes to the ultrafast formation of GB’* population. Given the more confined chromophore P-ring in LEA-A69T and lack of photoswitching, the GB’* formation should differ from the significantly twisted GB’ species discovered in LEA; one distinction is the clear ∼1340 cm^−1^ peak intensity decay beyond 1 ps that likely reflects a transiently formed and semi-trapped GB’* species in LEA-A69T. The more proximal His193 in LEA-A69T (Figure 5B) may contribute to the facile formation of GB’* through polar and electrostatic interactions, without the need for a significantly twisted chromophore conformation (e.g., a perpendicular orientation like in LEA GB’), which is corroborated by the stagnant excited-state peak frequency at ∼1345 cm^−1^ beyond the initial vibrational cooling that completes by ∼1 ps in LEA-A69T (Supplementary Figure S13D, blue trace) (Kumpulainen et al., 2017; Liu et al., 2017; Fang et al., 2019). In contrast, LEA exhibits a moderate frequency redshift time constant of ∼80 ps (Supplementary Figure S13D, red trace) inferring more P-ring flexibility in its local environment (Figure 5B). Furthermore, LEA does not show this transient Raman mode intensity rise or any indication of GB’* formation after 490 nm excitation (Figure 10C, red trace), supporting the previous interpretation that the “dead-end” GB’ population forms only during *off*→*on* photoswitching under 400 nm irradiation of LEA (Krueger et al., 2020), while the GB*→GA’ (*on*→*off*) photoswitching under 505 nm irradiation facilitates photoconversion.

## 4 Conclusion

In summary, a combination of spectroscopic methods including steady-state and time-resolved electronic spectroscopy, femtosecond stimulated Raman spectroscopy, and X-ray crystallography characterized the equilibrium and transient properties of a family of five engineered LEA-FPs with varying photoconversion and photoswitching abilities. The ncAA incorporation of an extra methyl group to the chromophore histidine ring greatly reduces the photoconversion efficiency *versus* LEA and the LEA-A69T mutant, but surprisingly enhances the dynamic photoswitching under 505 nm irradiation which can result in a larger red yield and red:green contrast under dual illumination than traditional 400 nm irradiation of LEA-H62X. The fs-TA spectroscopy unveiled largely similar electronic dynamics between LEA and LEA-H62X upon excitation of the green anionic chromophore, aided by the similar GS-FSRS spectra and crystal structures between these two FPs, revealing that the altered photochromic behavior mainly arises from the *trans*-like neutral chromophore *off* state. Meanwhile, some notable spectral signatures can be correlated with the onset degree (magnitude) and speed of characteristic chromophore twists across the molecular framework during the *on*→*off* photoswitching. Therefore, delineation of the chromophore photoswitching cycle needs to go beyond the initial starting point and transient dynamics by holistically considering the entire process from beginning to end (i.e., the final photoswitched state inside the FP), especially for the green “two-way streets” in reversible photoswitchable FPs like LEA and IrisFP that are also green-to-red photoconvertible.

Despite the lack of structural specificity, spectroscopies like fs-TA remain crucial to dissect the ultrafast photoresponse evidenced by the observation of an ∼40–60 ps twisting time constant in the photoswitchable LEA and LEA-H62X that is greatly reduced in the essentially nonphotoswitchable LEA-A69T. In particular, the equilibrium structural characterization of LEA and LEA-H62X suggests that the His62 ring remains coplanar, while the ultrafast spectroscopic measurements on the deprotonated chromophore (as a relevant model) suggest that a holistic ring twisting/repositioning event may lead to formation of a transient rotamer which could disrupt photoconversion. Our findings thus support the ring coplanarity to facilitate photoconversion; however, similar measurements of the protonated chromophore are necessary for a more direct proof of the photoconversion scheme. These notions are reinforced by the ES-FSRS spectra (after 490 nm excitation) comparing the nonphotoswitchable ALL-Q62H to LEA-A69T and LEA, wherein the vibrational spectral similarity of the excited-state chromophore unveils a relatively common origin. Notably, the mode-specific intensity and frequency dynamic patterns track the characteristic chromophore ring-twisting motions about the P-ring, I-ring, or His62 ring on ultrafast timescales with varied sensitivities, which depend on the *on*→*off* photoswitching rate and efficiency in the order of LEA (decent photoswitching, mainly P-ring twist), LEA-A69T (minimal photoswitching, some I-ring twist), and ALL-Q62H (nonphotoswitchable, mainly a His62 rotamer) (Kim et al., 2015; Krueger et al., 2023b). Transient Raman mode intensity dynamics contrasting LEA-A69T and LEA again showcase a more dynamic LEA chromophore; while evidence for the ultrafast formation of a semi-trapped GB*’ chromophore emerges for LEA-A69T with a pre-twisted P-ring and more flexible I-ring, which is a signature of its unique local environment.

We envision this collaborative investigation of a series of intimately related FPs displaying various degrees of photochromism will lay the groundwork and establish the potential synergy between equilibrium and nonequilibrium spectroscopies. The spectral data can be augmented by the exquisite structural information from X-ray crystallography, achieving a powerful toolset to bridge the gap across the crystalline and solution environments within the protein matrix. We note that the traditional methodology for improving the performance of FPs relies strongly on random mutagenesis and screening of large libraries which has become rather inefficient, stagnant, and at times wasteful. To achieve paradigm-shifting breakthroughs, an emphasis on bridging physical chemistry principles to properties relevant for biological applications can produce fundamental insights to enable the informed design of the chromophore and local environment. In this context, the captured excited-state structural insights in this work should be highly relevant for many other photochromic FPs, especially Kaede-like pcFPs with the engineerable His-ring via ncAA technology, which can display multiple photoinduced processes upon wavelength-dependent excitation and other light-induced “tricks” to benefit new applications and future bioimaging advances in life and health fields.

## Access codes

The atomic coordinates and structure factors for the LEA-A69T and LEA-H62X crystal structures have been deposited in the Protein Data Bank (PDB) as entries 8THS and 8UB6, respectively.

## Data Availability

The datasets presented in this study can be found in online repositories. The names of the repository/repositories and accession number(s) can be found in the article/Supplementary Material. Further inquiries can be directed to the corresponding author.
