# Supplementary material for: Capturing excited-state structural snapshots of evolutionary green-to-red photochromic fluorescent proteins
Source: Front Chem. 2023 Dec 7;11:1328081. doi: 10.3389/fchem.2023.1328081 (PMC10748491; doi:10.3389/fchem.2023.1328081)
Supplement: Supplementary file 1 [file DataSheet1.PDF]

Supplementary Material

Capturing excited-state structural snapshots of evolutionary green-to-red photochromic fluorescent proteins

Taylor D. Krueger, J. Nathan Henderson, Isabella L. Breen, Liangdong Zhu, Rebekka M. Wachter, Jeremy H. Mills, and Chong Fang*

*** Correspondence:** Chong Fang. E-mail: Chong.Fang@oregonstate.edu

Table of Contents

1 Supplementary Figures

Figure S1. Steady-state fluorescence spectroscopy of LEA, LEA-H62X, and LEA-A69T……..S2

Figure S2. Time-dependent absorption spectra under various illumination conditions…………S4

Figure S3. Calculated Raman spectra of the optimized and unoptimized chromophores……….S6

Figure S4. The unoptimized chromophore structures from three protein crystal structures…….S7

Figure S5. Experimental GS-FSRS spectra versus calculated Raman spectra………………….S8

Figure S6. Chromophore environments in LEA-A69T and LEA-H62X crystal structures.…...S10

Figure S7. ESI-QTOF-MS analysis of purified LEA-H62X protein sample……..…………….S11

Figure S8. Chromophore-environment interactions near His62 from crystal structures……...S12

Figure S9. Interactions of the internal charge network from crystal structures………………..S14

Figure S10. SE intensity dynamics from fs-TA spectra of LEA, LEA-H62X, LEA-A69T…....S15

Figure S11. Contour plots of ES-FSRS spectra with a reduced Raman pump power……...….S16

Figure S12. Intensity dynamics from ES-FSRS with 2.5 vs. 4 mW Raman pump power….....S17

Figure S13. Frequency dynamics from ES-FSRS with the reduced Raman pump power…....S19

2 Supplementary Tables

Table S1. Ground and excited state experimental FSRS peak frequencies of various FPs…….S21

Table S2. Calculated GS Raman and experimental GS-FSRS frequencies of LEA………..........S22

Table S3. Calculated GS Raman and experimental GS-FSRS frequencies of LEA-A69T…….S23

Table S4. Calculated GS Raman and experimental GS-FSRS frequencies of ALL-Q62H……S24

Table S5. Calculated GS Raman and experimental GS-FSRS frequencies of LEA-H62X……S25

Table S6. Crystallographic statistics for the LEA-A69T and LEA-H62X structures……….…S26

Table S7. Key crystallographic observables for LEA, LEA-A69T, and LEA-H62X ……...….S27

3 Supplementary References……………………………………………………………….S28

1. Supplementary Figures

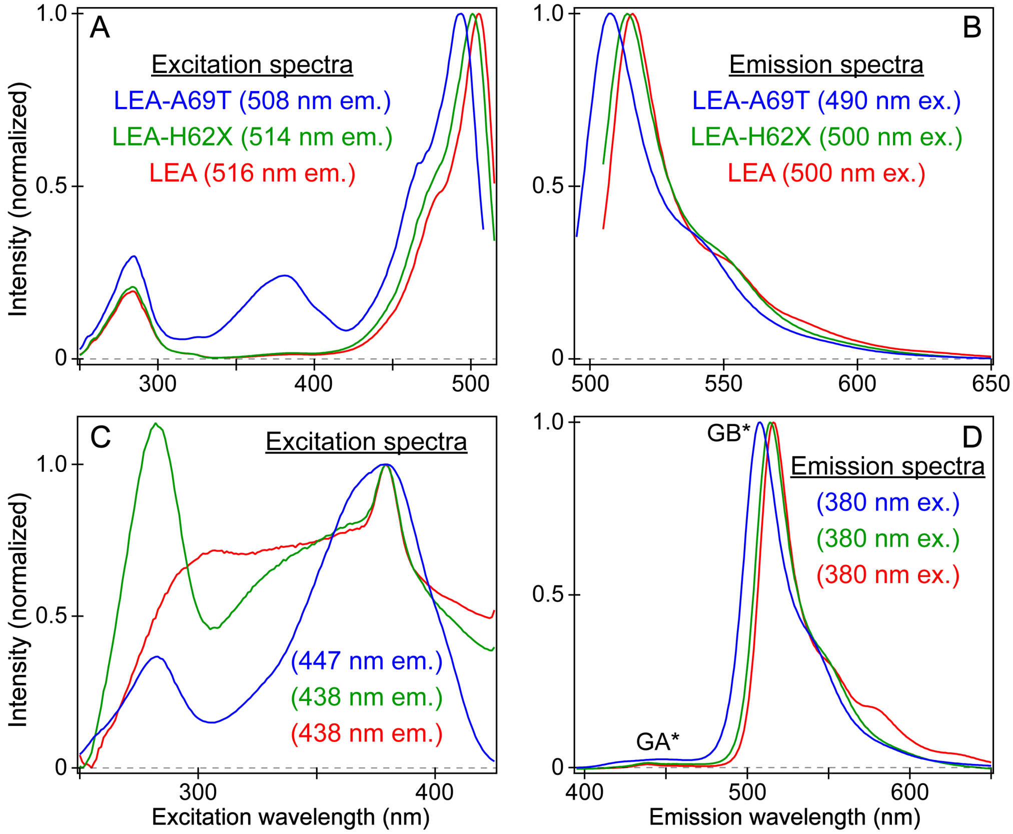

Figure S1. Normalized excitation/emission spectra of LEA (red), LEA-H62X (green), and LEA-A69T (blue) for the **(A/B)** GB* and **(C/D)** GA* states, respectively. The emission (em.) detection wavelengths for the excitation spectra and the excitation (ex.) wavelengths for the emission spectra are listed in the insets. The emissive species of GA* and GB* are labeled near their respective fluorescence bands in panel **(D)**.

Notably, the excitation and emission spectra of the deprotonated green chromophore (GB) reveal several interesting trends (Figure S1A,B). Both spectra follow the peak wavelength trend observed in the steady-state electronic absorption spectra (Figure 1 in main text), where LEA-A69T absorbs/emits the bluest light while LEA absorbs/emits the reddest light with LEA-H62X in-between. We note that the observed absorption and emission peak wavelengths herein do not follow a recently discussed trend found in many FPs, where the absorption/emission peak blueshifts and scales linearly with increasing fluorescence quantum yield (FQY) values (Mukherjee et al., 2022; Chen et al., 2023b). In contrast, LEA absorbs/emits at the reddest wavelengths yet possesses the highest FQY (0.81) from the deprotonated green chromophore (Kim et al., 2015). By observing the GB* emission from 508–516 nm, the excitation spectra reveal a dominant contribution from the GB absorption peak at ~500 nm (Figure S1A). Interestingly, a contribution from the neutral green chromophore (GA) is also observed in the excitation spectra. The contribution is weak for LEA and LEA-H62X where a minor peak at ~380 nm is observed; however, a rather strong contribution from the GA/GA* species is observed in LEA-A69T. This finding is certainly influenced by the elevated pK_a of the LEA-A69T chromophore (8.4), meaning that there is a significantly larger protonated chromophore population present at the buffer pH of 7.9 (Kim et al., 2015; Krueger et al., 2023b) and some of the populations can undergo excited-state proton transfer (ESPT) to result in the GB* emission (Fang et al., 2009; Meech, 2009).

The excitation and emission spectra from the neutral chromophore are presented in Figures S1C,D for three pcFPs in this work. In all three pcFPs, the GA* emission intensity is weak (Figure S1D) with a minor peak at ~447 nm in LEA-A69T and at ~438 nm in LEA/LEA-H62X. The weak fluorescence from the protonated form reinforces the notion that the GA* population prefers other nonradiative pathways including ESPT, conformational motions including the ring-twist-induced isomerization and internal conversion, and FRET (Meech, 2009; Subach and Verkhusha, 2012; Krueger et al., 2023a). The dominant emission peak from the I* (denoting a deprotonated chromophore in an unrelaxed protein environment) or GB* species upon excitation of GA supports at least one of these three pathways. Moreover, the I*/GB* peak maximum remains identical (i.e., 508 nm for LEA-A69T, 514 nm for LEA-H62X, and 516 nm for LEA) regardless of the excitation wavelength, either 380 or 490–500 nm (Figure S1B,D), implying that the excited-state population reaches a similar fluorescent state upon excitation of GA or GB species. Current work is ongoing to dissect these three mechanisms on ultrafast timescales and may provide useful insights regarding potential pathways that compete with the green-to-red photoconversion upon UV/near-UV excitation of the GA species. Moreover, LEA displays pronounced vibronic coupling after 380 nm photoexcitation, evidenced by the prominent shoulder bands at ~550, 580, and 625 nm in the emission spectrum (Figure S1D, red trace).

The elevated GA* emission observed for LEA-A69T upon 380 nm excitation is likely influenced by the larger GA population due to the increased pK_a; however, the GA* emission is still greatly reduced (Figure S1D) relative to the GA absorption intensity (Figure 1D). This result provides evidence that the GA/GA* population in LEA-A69T prefers the *off→on* photoswitching (which likely involves ESPT) to a larger degree than LEA/LEA-H62X, which is currently being investigated on ultrafast timescales upon 400 nm excitation (a common wavelength using a Ti:sapphire fs-laser system). By observing the weak emission from ~438–447 nm, the excitation spectra reveal a contribution from the GA species at ~380 nm in all three pcFPs (Figure S1C). Given the weak GA* emission and the minor GA population observed for LEA/LEA-H62X in pH 7.9 buffers, the excitation spectra appear broad when normalized because the absolute intensity is quite weak. However, a sharp peak is observed in LEA and LEA-H62X at ~380 nm which likely represents the specific GA population that is responsible for the GA* emission around ~438 nm. In contrast, with a much larger GA population in LEA-A69T (Figure 1D), the excitation spectrum appears more normal with a strong contribution from the GA population being observed as a band centered at ~380 nm, which essentially overlaps with the electronic absorption spectrum (colored in blue, Figure 1D). In addition, LEA-A69T may possess both the *cis*- and *trans*-protonated chromophores which can contribute to the broadness of the GA spectrum as well as the excitation spectrum (colored in blue) in Figure S1C (Tang and Fang, 2022).

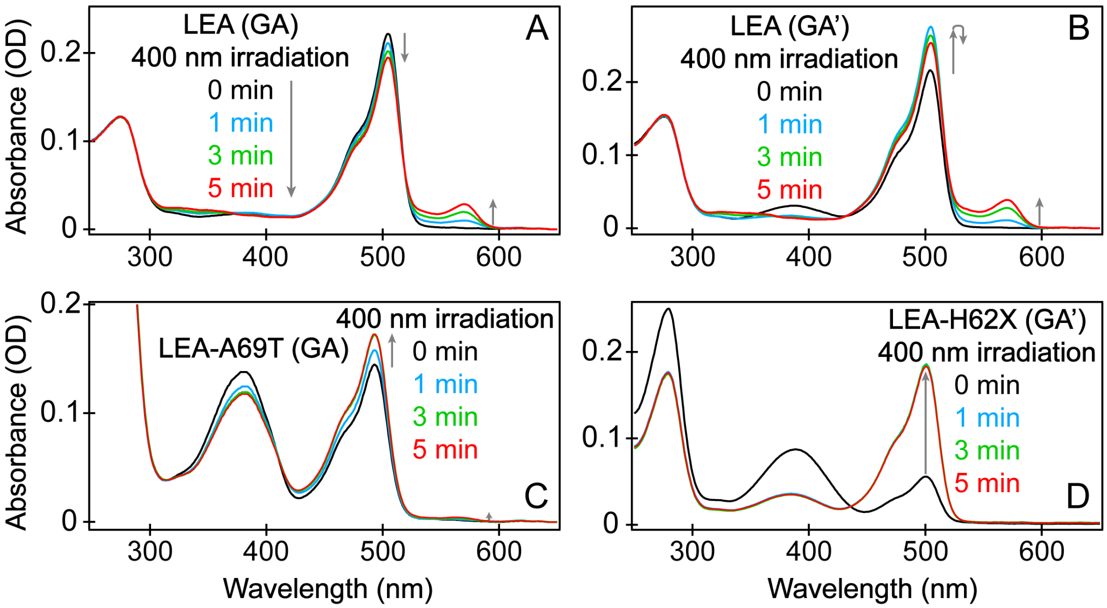

Figure S2. Time-dependent electronic absorption spectra of **(A)** LEA (GA state), **(B)** LEA (GA'), **(C)** LEA-A69T (GA), and **(D)** LEA-H62X (GA') under 400 nm LED irradiation. The absorption spectra were collected at time zero (black), 1 min (cyan), 3 min (green), and 5 min (red) of irradiation to focus on the initial photoresponse of GA/GA' without added complications from photoconversion and photoswitching. The 400 nm LED irradiation and absorption measurements on GA' (**B** and **D**) were performed after a two-hour pretreatment of 505 nm LED irradiation to generate an appreciable GA' population. See the Methods section in main text for the pertinent experimental details. The time progression is denoted by the gray arrow(s) in each panel.

The initial photoresponse of the GA and GA' states in LEA are compared (Figures S2A,B), where the chemically (pH)-induced population in LEA (termed GA) shows no evidence for the *off*→*on* photoswitching, although it likely does occur to a degree but is masked by the green-to-red photoconversion (i.e., the growth of the RB peak at ~570 nm and the ensuing GB→GA equilibrium). This interpretation is evidenced by the decay from time zero (Figure S2A, black trace) of the GB absorption peak after five minutes of 400 nm irradiation (Figure S2A, red trace). In contrast, after a two-hour pretreatment of 505 nm LED irradiation to generate an appreciable GA' population (Figure S2B), the initial photoresponse is significantly altered in LEA. In particular, after just one minute of 400 nm illumination (Figure S2B, cyan trace), a pronounced rise of the GB absorption peak is observed compared to the time zero spectrum (Figure S2B, black trace). This result shows that the GA' species is more prone to the *off*→*on* photoswitch back to the initial GB state instead of photoconversion. After the first minute of 400 nm illumination, the GB peak decreases due to photoconversion and the ensuing GB→GA equilibrium as the GA/GA' species is consumed to form the deprotonated red chromophore (RB). Although the GA' species is more prone to photoswitching, the elevated GA/GA' population after the two-hour pretreatment still produces a larger RB population, thereby proving that the GA' species can photoconvert, albeit at a reduced proclivity relative to the GA population. The prominent *off*→*on* photoswitching of GA' species in LEA explains why the sequential illumination of LEA does not significantly improve the photoconversion yield (Krueger et al., 2020): even though a large GA/GA' photoconvertible population is present after the pretreatment, the photoswitched *off* state quickly recovers the GB population after one minute of 400 nm illumination, essentially negating the pretreatment effect. In contrast, dual illumination is constantly replenishing the GA' population so the 505 nm light-induced effect on the photoconversion yield is constantly occurring for LEA.

Similarly, the initial photoresponse of the GA population in LEA-A69T and the GA' population in LEA-H62X are compared (Figure S2C,D). LEA-A69T does not show evidence for the *on→off* photoswitching or GA' formation (Krueger et al., 2023b), so only the initial photoresponse of the chemically induced protonated chromophore population (GA) can be observed in the single-site mutant. Notably, a continuous rise of the GB absorption peak is observed after one, three, and five minutes of 400 nm illumination (Figure S2C) with a minor rise of the ~570 nm peak, which indicates some photoconversion and RB formation but at a much reduced extent versus LEA. The rise of the GB absorption peak after one minute of 400 nm illumination of GA population in LEA-A69T (Figure S2C) is similar to the GB absorption peak rise after one minute of 400 nm illumination of GA' in LEA (Figure S2B), indicating that both populations prefer the *off→on* photoswitching. In contrast to the 400 nm illumination of LEA, the GB absorption peak of LEA-A69T continues to rise during 400 nm illumination. This result offers several interesting interpretations as follows. First, the GA species in LEA-A69T greatly favors *off→on* photoswitching which involves proton transfer and the chromophore *trans→cis* isomerization. Second, the GA species in LEA-A69T likely shares similar properties to the GA' species in LEA regarding their preference for the *off→on* photoswitching over photoconversion. Third, the GA species in LEA-A69T and GA' species in LEA favors photoswitching to a larger degree than the GA species in LEA (Figure S2A). For comparison, the photoresponse of GA' in LEA-H62X (Figure S2D) is similar to the GA' photoresponse in LEA (Figure S2B); however, the prominent *on→off* photoswitching in LEA-H62X results in a more dramatic effect. After the two-hour pretreatment of LEA-H62X under 505 nm light irradiation, the GA/GA' population is significantly larger than the GB population (Figure S2D, black trace). Interestingly, after just one minute of 400 nm illumination (Figure S2D, cyan trace), the pronounced GA' population is quickly photoswitched back to the GB state, which recovers most of the initial population with a greatly reduced photoconversion yield evidenced by the miniscule growth of the ~570 nm peak (also see Figure 1F in main text). The timescale and mechanism for the GA/GA'→GB transition during *off→on* photoswitching of these pcFPs are currently being investigated by fs-TA and ES-FSRS with suitable resonance conditions, given its different focus from the current work.

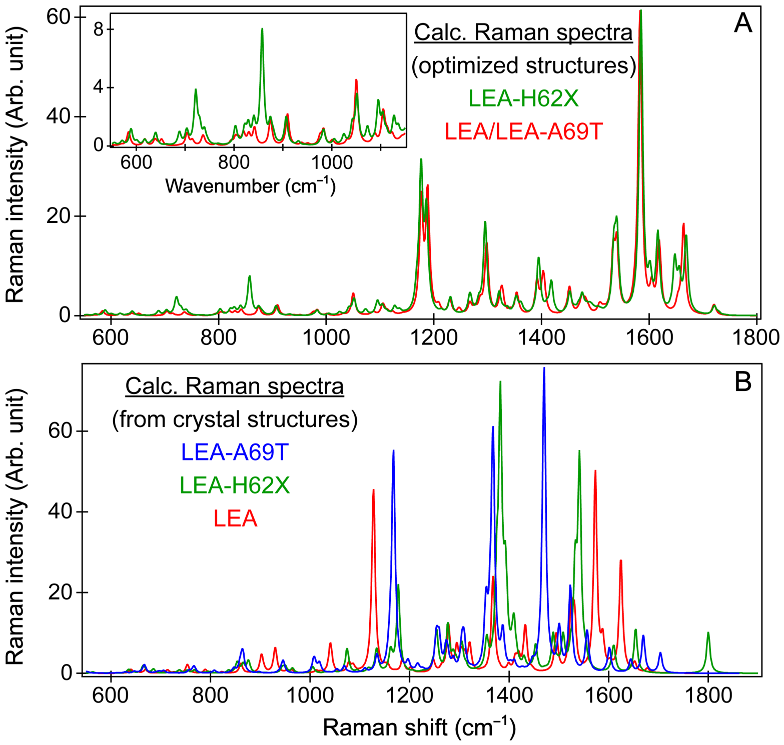

Figure S3. Calculated Raman spectra of the HYG chromophore from the **(A)** DFT-optimized structures using Gaussian and **(B)** the unoptimized structures taken directly from the crystal structures of LEA (red), LEA-H62X (green), and LEA-A69T (blue). The **(A)** inset zooms in on the calculated spectra in the low-frequency region from ~500–1150 cm^{-1}. The Raman frequency values are unscaled for direct comparison. More details regarding the DFT calculations can be found in the Experimental section.

The calculated Raman spectra of LEA, LEA-H62X, and LEA-A69T are shown for the optimized and unoptimized chromophore structures taken directly from the crystal structures (Figure S3). The calculation details can be found in the Materials and Methods section (main text). The overall similarity of the calculated spectra from the optimized structures reproduces the significant overlap of vibrational peaks for all three pcFPs in the GS-FSRS spectra (Figure 3); thus, the optimized structures were used for mode assignments. The largest differences in these calculations occur in the low-frequency region (<1000 cm^{-1}), reminiscent of the experimental spectra. This pattern can be explained by the His62 ring remaining unconjugated in the green chromophore, so it is unlikely to incur strong effects even with the extra methyl group in LEA-H62X. The optimized structures of LEA and LEA-A69T (with crystal structures as the starting points) converge to a similar structure *in vacuo*. Meanwhile, low-frequency peaks typically involve skeletal motions throughout the chromophore framework (Fang and Tang, 2020) that are sensitive to motions of the His62 ring, hence the observed differences in certain regions.

In contrast, the calculated Raman spectra taken directly from the crystal structures without further optimization differ significantly for LEA, LEA-H62X, and LEA-A69T (Figure S3B). The significant variation of the calculated spectra is not reproduced in the experimental GS-FSRS spectra (Figure 3), so these calculations were not used for the mode assignment but provide helpful insights. The altered frequencies and intensity patterns between three pcFPs from the crystal structures are representative and may indicate that the crystalline environment differs notably from the solution environment (Konold et al., 2020; Wang et al., 2023). We also note that both calculated spectra in Figure S3A,B are greatly simplified and mainly serve as qualitative reference; for a detailed mode assignment, advanced QM/MM calculations that account for various chromophore-environment interactions are necessary.

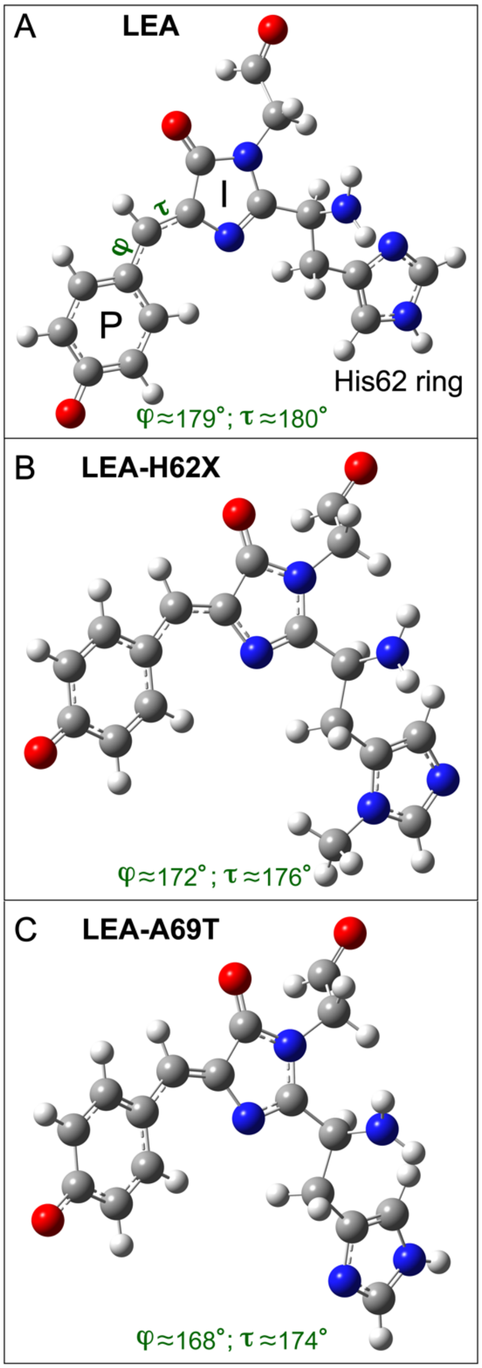

Figure S4. Depiction of the unoptimized chromophore structures taken directly from crystal structures of **(A)** LEA (PDB: 4DXN), **(B)** LEA-H62X (PDB: 8UB6), and **(C)** LEA-A69T (PDB: 8THS) (also see Figure S6). One set of frequency calculations were made directly using these chromophore structures to generate the ground-state Raman spectra in Figure S3B above and Figure S5 below. The bridge CCCC dihedral angles between the P- and I-rings (labeled in panel **A**) are denoted in each panel (φ and τ between the chromophore P- and I-rings). The dihedral angle along the CC bond adjacent to the His62 ring remains largely conserved at ~180° (i.e., coplanar: 179° in LEA, 176° in LEA-H62X, and 178° in LEA-A69T), while CCCC bridge dihedrals connecting I-ring to His62 from the unoptimized crystal structures exhibit some variations between LEA (165°), LEA-H62X (161°), and LEA-A69T (163°), in accord with the increased flexibility of that bridge. Overall, these dihedral angles show less variation than P-to-I-ring dihedral. Atomic colors: carbon, gray; nitrogen, blue; oxygen, red; hydrogen, white.

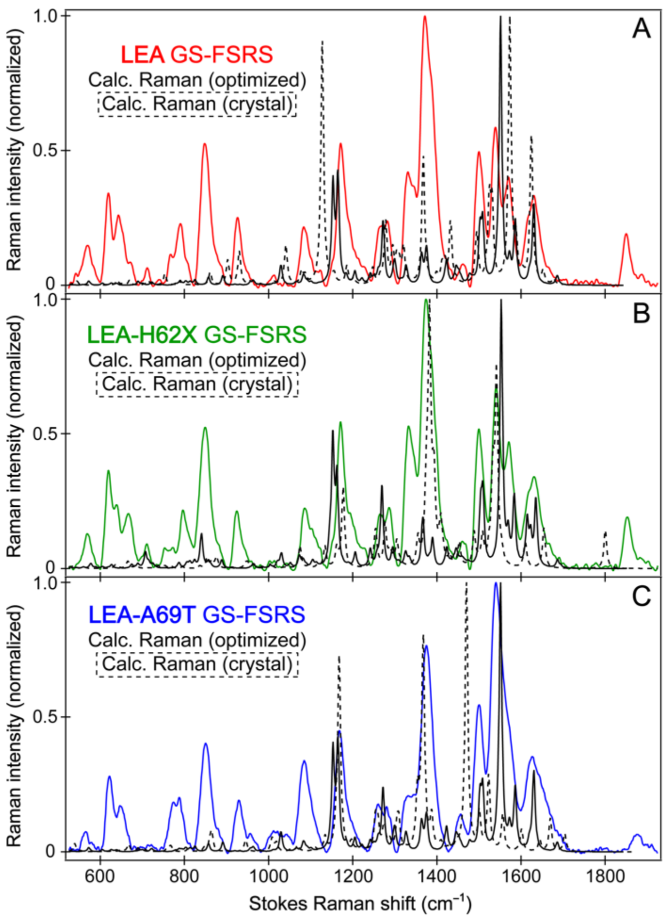

Figure S5. A comparison of the experimental GS-FSRS spectra (solid, color-coded) from **(A)** LEA (red), **(B)** LEA-H62X (green), and **(C)** LEA-A69T (blue) to the calculated Raman spectra from the DFT-optimized (solid black) and crystal (dashed black) structures. GS-FSRS spectra were collected with a 540 nm Raman pump (see Figure 3 in main text). The calculated mode frequencies of the optimized chromophore structures were scaled by 0.98 to better match the experimental spectra.

The experimental GS-FSRS spectra are compared to the calculated Raman spectra from the optimized (Frisch et al., 2016) and unoptimized chromophores taken from the recently acquired crystal structures (see Figure S6). Note that in some cases, the calculated Raman peaks from the unoptimized structures match the experimental spectra better (e.g., the ~1370 cm^{-1} peak matches better in the LEA-H62X and LEA-A69T spectra); however, overall the optimized structure calculations produce a better match. This is supported by the overall similar experimental GS-FSRS spectra and calculated Raman spectra (from the optimized structures, see Figure S3A) among the three pcFPs. The reason for a better match between the experimental and calculated spectra from the optimized structures in some cases, and a better match from the unoptimized structures in other cases, is multifold. A combination of the optimized and unoptimized structures is likely more realistic that could be achieved by advanced QM/MM simulations (Schapiro et al., 2019; Cui et al., 2021). In the DFT calculations herein, the optimized structures were performed *in vacuo* that do not account for chromophore-environment interactions. On the other hand, the unoptimized chromophore structures may change in solution versus the crystalline environment for the protein (Konold et al., 2020). We also note that the low-frequency peaks below ~1100 cm^{-1} are significantly more intense in the experimental spectra than the calculated spectra, likely due to the resonance contributions from stimulated Raman that are not accounted for in these off-resonance calculations representing the spontaneous Raman scattering (Fang et al., 2019).

Notably, a broad comparison of the LEA and LEA-H62X experimental GS-FSRS spectra shows that most of the peak frequencies are within 2–3 cm^{-1} of each other (see Table S1 below), reinforcing the FPs' similar chromophore conformation and local environment. However, several shifted Raman peaks are worth mentioning: the 1465, 1374, 1282, and 1084 cm^{-1} modes in LEA versus the 1460, 1376, 1286, and 1086 cm^{-1} modes in LEA-H62X that consist of various motions including the C=N stretch on His62, H-rocking motions and C–C stretch on the carbon bridge connecting the I-ring to His62, P-ring CC and I-ring C–N stretch with H-rocking across ring system, and H-scissoring on P-ring. Overall, these fairly delocalized modes are a good probe of local environment differences sensed by the protein chromophore. Several of these motions are more centered near the His62 end of the chromophore, which experiences larger changes between LEA and LEA-H62X (e.g., 1465 versus 1460 cm^{-1} modes), while the localized modes centered on the I- or P-rings are more similar in frequency. These subtle spectral changes indicate that the slightly altered microenvironments may influence the pronounced macroscopic photoswitching behavior (as shown in Figure 1E,F and Figure S2).

In general, the Raman spectra of the LEA and ALL-FPs can be divided into three regions (see Figure 3). First, high-frequency peaks (~1500–1650 cm^{-1}) primarily involve the localized C=C, C=N, and C=O stretching motions on the chromophore's P- and I-rings. Second, medium-frequency peaks (~1000–1500 cm^{-1}) are comprised of H-motions along with bridge C–C and C–N stretch in addition to ring deformation modes. Third, low-frequency peaks (<1000 cm^{-1}) involve more global skeletal motions throughout the chromophore from bending, HOOP to deformation modes (Esposito et al., 2001; Tozzini et al., 2003; Fang et al., 2009; Taylor et al., 2019; Fang and Tang, 2020). There are several Raman modes that show a continuous frequency shift from LEA/LEA-H62X/LEA-A69T/ALL-Q62H including the 1630/1629/1625/1624 cm^{-1} and 1465/1460/1456/1450 cm^{-1} modes (Table S1). The higher-frequency peak involves C=C stretch on the P-ring and bridge between P- and I-rings, while the lower-frequency peak involves C=N stretch on His62; therefore, these two motions can effectively probe "book-end" modes that are sensitive to opposite ends of the chromophore. The variation of the C=C stretch peak can be rationalized by the twisted P-ring (see Section 3.3 in main text) and its altered local environment between LEA/LEA-H62X and LEA-A69T/ALL-Q62H, wherein a more compact cavity with a closer His193 can promote electron delocalization (e.g., π-π stacking) in the latter FPs (Krueger et al., 2023b) and lead to the minor yet discernible mode frequency redshift. The C=N stretch peak on His62 is likely influenced by the different local environments near the histidine ring, while the effects of varied P- and I-ring conformations and their local environments on His62 motions are minor due to the unconjugated nature of the His62 ring. Furthermore, several GS-FSRS peaks can be grouped and compared for LEA/LEA-H62X vs. LEA-A69T/ALL-Q62H (Table S1), such as 1263/1264 vs. 1262/1261 cm^{-1} and 1172/1171 cm^{-1} vs. 1169/1167 cm^{-1} peaks. The mode assignment for these peaks can be found in Tables S2–S5 below for various FPs. In particular, the ~1170 cm^{-1} mode involves P-ring H-scissoring and H-rocking of the methine bridge connecting the P- and I-rings, which displays a frequency difference due to the altered chromophore P-ring interactions with His193 and the Thr69-induced changes to the local environment (Kim et al., 2015; Krueger et al., 2023b). Such detailed comparisons substantiate GS-FSRS as a sensitive structural technique for various FP chromophores, and photosensitive biomolecular systems in general (Dietze and Mathies, 2016; Fang and Tang, 2020).

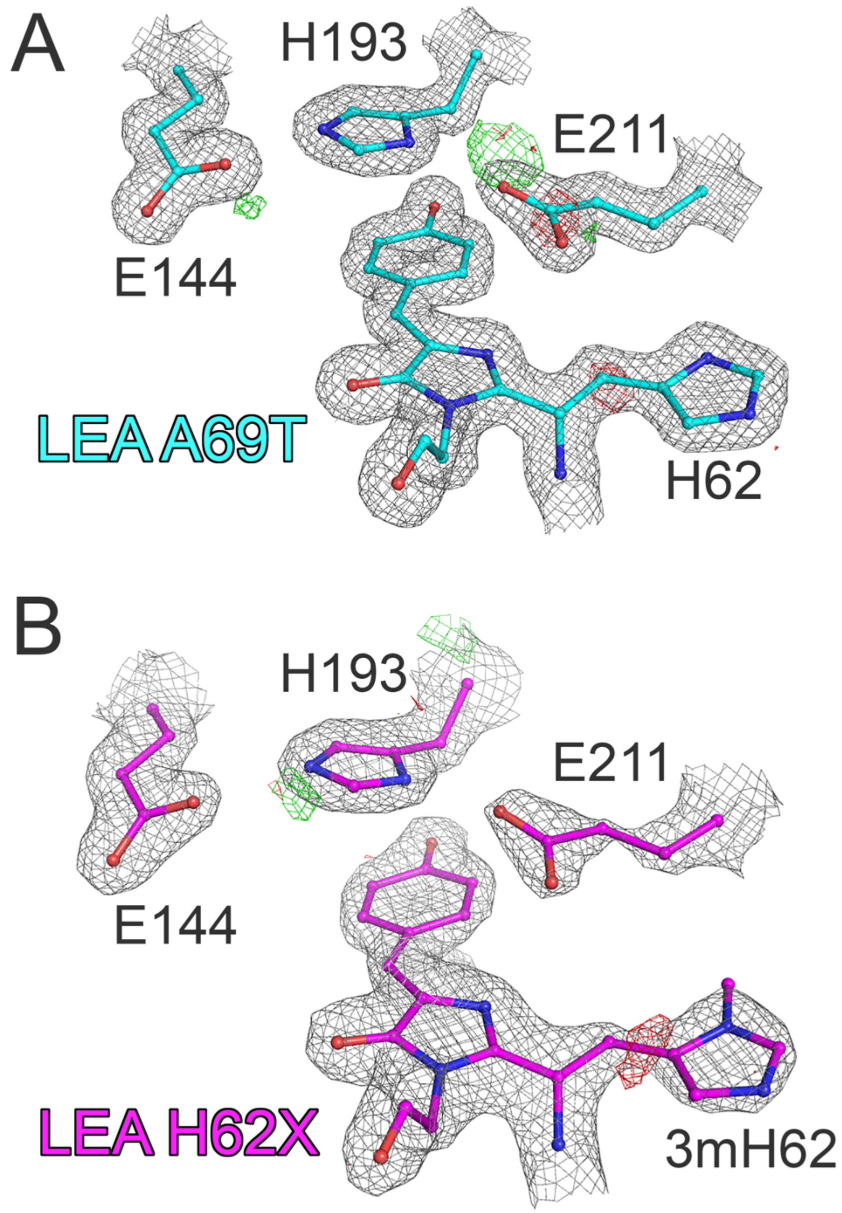

Figure S6. Depiction of the chromophore environments in the **(A)** LEA-A69T (cyan) and **(B)** LEA-H62X (magenta, 3mH62 stands for 3-methyl-histidine at residue position 62) structures with select residue sidechains and chromophore in ball-and-stick representation (cyan/magenta = carbon, blue = nitrogen, and red = oxygen). $2F_o$-F_c electron density maps (gray mesh) are shown contoured at 1.5 σ, and the F_o-F_c difference density maps (positive in green and negative in red) are contoured at 2.5 σ.

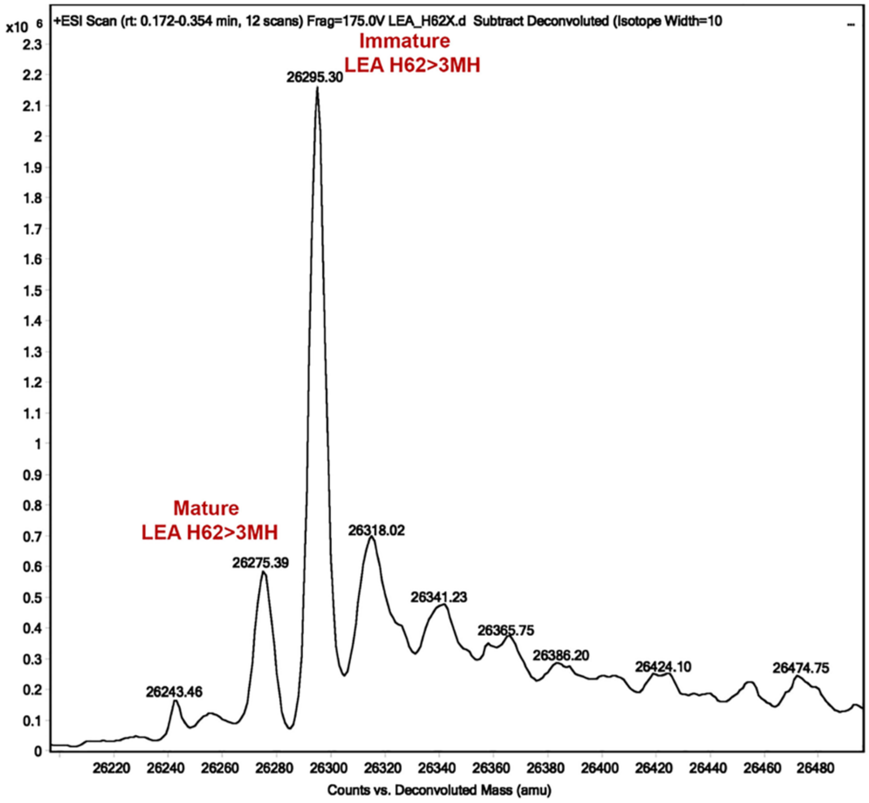

Figure S7. Electrospray ionization quadrupole time-of-flight mass spectrometry (ESI-QTOF-MS) analysis of the purified LEA-H62X (X = 3MH, 3-methyl-histidine) protein sample showing major peaks that correspond to the immature and mature forms of the chromophore (red labels by the two respective peaks).

From our mass spectrometry data on both LEA and LEA-H62X, we observed that inefficient chromophore maturation only occurs in the latter protein with the noncanonical amino acid. The primary reason for collecting the mass spectrometry data was to confirm the presence of the 3-methyl-histidine and we happened to observe that a large percentage of the protein had a measured mass consistent with immature LEA incorporating 3MH at residue 62 (see red peak labels in Figure S7, and Section 3.2 in main text). This finding helps to explain the observation that, relative to the other LEA variants, LEA-H62X exhibits a lighter green color (in its green form/state) at comparable protein concentrations. We also confirmed that both the immature and mature LEA-H62X are present in the prepared sample batch and, most importantly, their measured masses agree very well (<1 amu difference) with the corresponding predicted average atomic masses.

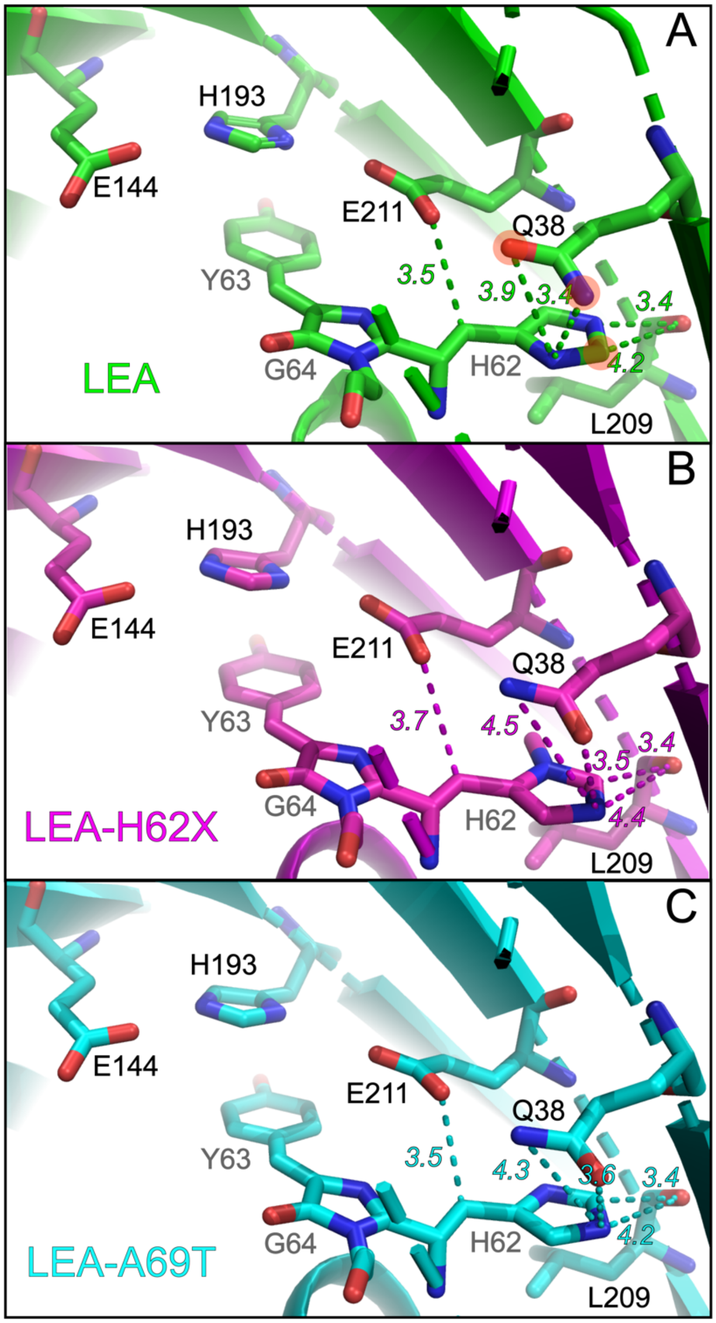

Figure S8. Key interactions with local environment near the chromophore His62 end for **(A)** LEA (green), **(B)** LEA-H62X (pink), and **(C)** LEA-A69T (cyan). The color-coded dashed lines and values highlight key chromophore-local-environment interactions with His62 and the respective distances (in Å unit). The HYG chromophore residues are labeled in gray to contrast with several nearby residues labeled in black. Atomic colors: nitrogen, blue; oxygen, red. H atoms are omitted for clarity.

The chromophore-environment interactions noticeably differ between the three pcFPs near the His62 ring (Figure S8). Interestingly, the His62 ring is flipped in the LEA-H62X and LEA-A69T crystal structures versus that of LEA, which could play a role in the greatly reduced photoconversion efficiencies of the mutant FPs versus LEA (Krueger et al., 2023b). In addition, the Gln38 residue is also flipped in the mutant pcFPs (compared to LEA) to maintain those polar interactions between the

Gln38 sidechain amide and His62 sidechain ring, wherein the distances between the His62 ring-N atom and adjacent Gln38 sidechain amide are longer (hence with higher flexibility) in the mutant pcFPs. We note that the orientation places the Gln38 nitrogen next to the Glu211 carboxylate oxygen in LEA-H62X and LEA-A69T (Figure S8B,C), which differs from LEA (Figure S8A) with a more flexible local environment and potential reverse protonation of Glu211 and His193 in concert with a flipped Gln38 conformation to possibly facilitate photoconversion (Kim et al., 2013; Kim et al., 2015). For corroboration of the new structures presented in this work at neutral pH 7.5 (Section 2.1.3), we also measured the distances between the far-end carbon atom of the His62 ring (adjacent to the N atom currently denoted in Figure S8A, so it is reminiscent of the N atom location of a flipped His62 ring in Figure S8B,C) and the Gln38 residue sidechain O and N atoms. The corresponding distances from His62 to Gln38 in LEA (4.6, 3.7 Å: see red-circle highlights in Figure S8A) are slightly larger than the distances listed in LEA-H62X (4.5, 3.5 Å) and LEA-A69T (4.3, 3.6 Å). This comparison may hint the altered interactions between His62 and Gln38 in LEA, supported by the formation of a His62 rotamer in the Q38A mutant (with a larger cavity around the His62 ring end) that disrupts photoconversion (Kim et al., 2013; Kim et al., 2015; Krueger et al., 2023b), which represents more of the cases for LEA-H62X and LEA-A69T.

Perhaps the greatest change among the three pcFPs is the distance between the C_α atom and Glu211 sidechain carboxylic group, which is the largest in LEA-H62X (3.7 Å) versus LEA and LEA-A69T (3.5 Å). This is primarily due to the extra methyl group on the histidine ring in LEA-H62X, which causes the ring to shift horizontally away from the catalytically relevant Glu211 relative to LEA and LEA-A69T (Kim et al., 2013; Kim et al., 2015). The consequence is a great reduction in the apparent photoconversion efficiency of LEA-H62X (Figure 1F) as well as its enhancement of photoswitching behavior (Figure 1E), showcasing their dependence on different factors from the crystallographic and spectral features.

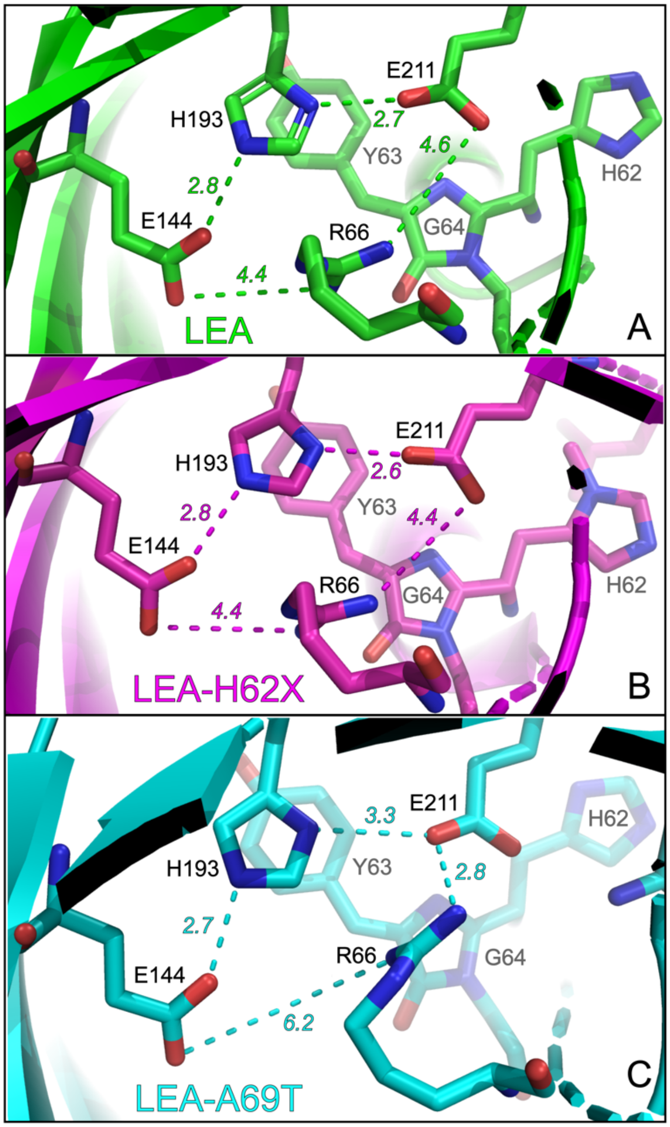

Figure S9. Key polar interactions along the charge network around the chromophore for **(A)** LEA (green), **(B)** LEA-H62X (pink), and **(C)** LEA-A69T (cyan). The color-coded dashed lines with values highlight key interactions between E144, H193, E211, and R66 with the respective distances (in Å unit). The HYG chromophore residues are labeled in gray to contrast with several nearby residues labeled in black. Atomic colors: nitrogen, blue; oxygen, red. H atoms are omitted for clarity.

The interactions between the internal charge network Glu144, His193, Glu211, and Arg66 are shown between the three pcFPs. This charge network has been invoked to play a key role to maintain the bright green fluorescence of the deprotonated chromophore in the *cis* conformation (Kim et al., 2015; Krueger et al., 2023b). The differences between LEA and LEA-H62X are relatively minor, implying that these residues and interactions with the chromophore are not the reason for the different *on→off* photoswitching behavior, which could instead arise from different H-bonding networks in the *trans* protonated conformation (i.e., *off* state). The Thr69-induced changes in LEA-A69T contribute to significant variations in the interactions between these four residues, largely influenced by a reoriented Arg66 that H-bonds with Thr69. These altered interactions can greatly contribute to the much reduced *on→off* photoswitching behavior observed under 505 nm illumination of LEA-A69T (Figure 1E). In addition, though partially influenced by a slightly different "top-down" perspective in LEA-A69T, the

better overlap between His193 and the chromophore P-ring can be seen for LEA-A69T in Figure S9C compared to LEA and LEA-H62X in Figure S9A,B. Towards the other end of the chromophore, the notably shortened polar-bond distance between Arg66 and Glu211 in LEA-A69T may inhibit Glu211 from playing a prominent role in the photoconversion mechanism that has been invoked previously (Kim et al., 2015).

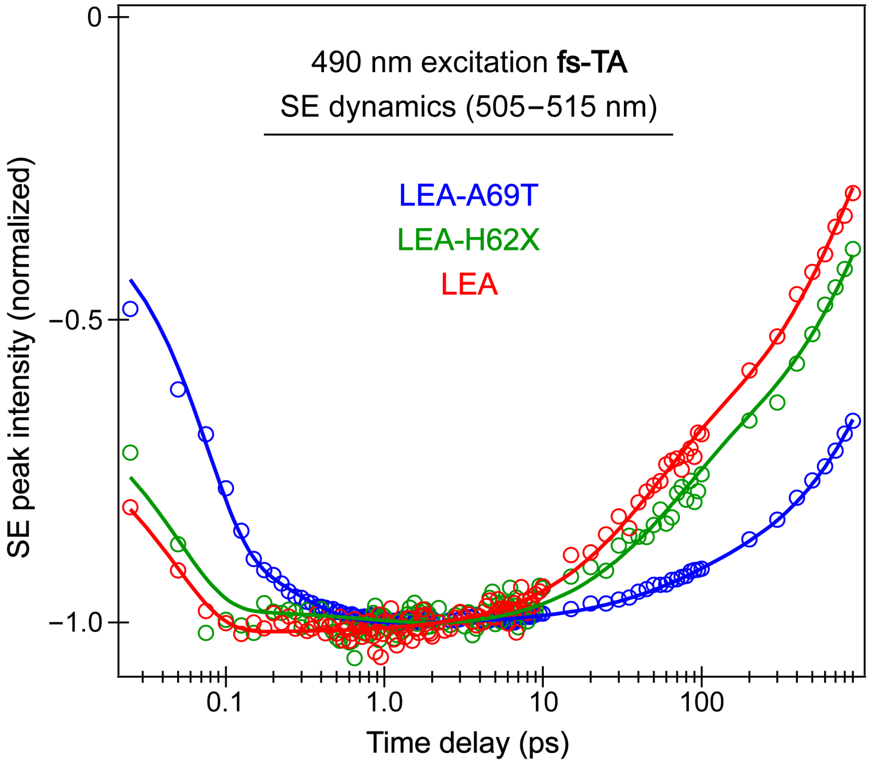

Figure S10. Normalized stimulated emission (SE) intensity dynamics from fs-TA measurements of LEA (red), LEA-H62X (green), and LEA-A69T (blue) after 490 nm excitation in pH 7.9 buffer solution. The probe-dependent intensity dynamics reflect a peak integration region from 505–515 nm, the color-coded data points (hollow circles) are overlaid with the least-squares multi-exponential fits (solid curves). The spectral traces are normalized at ~2 ps for a clear comparison.

The GB* main SE band intensity dynamics are compared between LEA, LEA-H62X, and LEA-A69T upon 490 nm excitation of GB species (Figure S10) to emphasize the lengthened excited-state lifetime of LEA-A69T and its greatly reduced intermediate decay on the 40–60 ps timescale compared to the other two pcFPs (Krueger et al., 2023b). The slightly lengthened and reduced intermediate decay of LEA-H62X is made apparent upon comparison to the intensity dynamics of LEA that shows the fastest overall decay on the tens to hundreds of ps timescale (red trace in Figure S10, also evident in Figure 7 in main text).

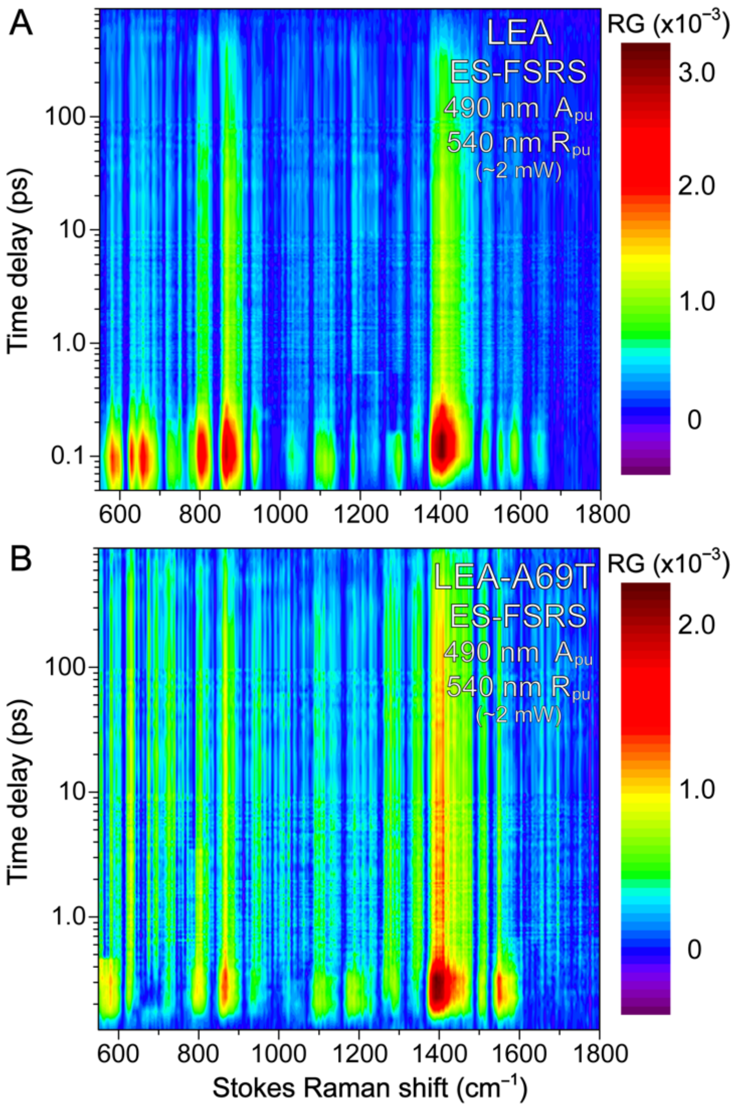

Figure S11. Contour plots for the excited state (ES)-FSRS spectra of **(A)** LEA and **(B)** LEA-A69T in pH 7.9 buffer solution. The spectra were collected with the actinic pump (A_{pu}) and Raman pump (R_{pu}) wavelengths of 490 and 540 nm, respectively, with an average R_{pu} power of ~2.5 mW. The Raman probe is on the red side (Stokes side) of R_{pu}. The color-coded signal intensity levels are shown to the right of each plot as Raman gain (RG, $\times 10^{-3}$). Note that the associated ES-FSRS data with about double the R_{pu} power were plotted in Figure 8A and C, respectively.

The 2D-contour plots for the ES-FSRS spectra of LEA and LEA-A69T collected with a lower R_{pu} power of ~2.5 mW are shown (Figure S11), providing a valuable comparison to the ES-FSRS spectra obtained with a higher power of ~4 mW (Figure 8 in main text). The ES-FSRS peak intensity dynamics from these plots in Figure S11 are shown in Figure 10 (main text), while the center peak frequency dynamics are displayed in Figure S13 with data analysis. In general, the transient vibrational peak frequencies and spectral patterns in ES-FSRS data are conserved, regardless of the R_{pu} power within the 2.5–4 mW range. The intensities of the ES-FSRS peaks, however, are noticeably lower with the lower R_{pu} power (see Figures 8 and S12), which also exhibits some effects on the observed peak intensity dynamics (McCamant et al., 2004; Dietze and Mathies, 2016; Fang et al., 2019).

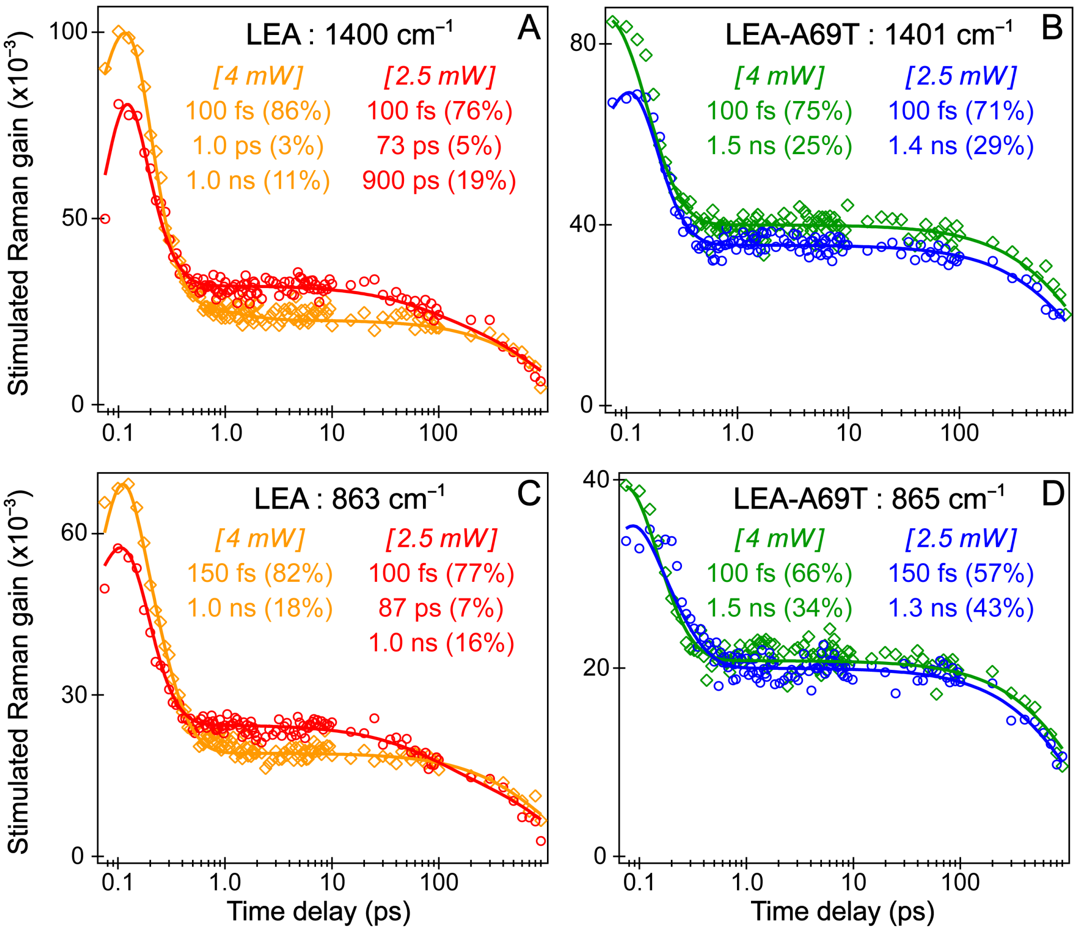

Figure S12. Comparison of Raman peak intensity dynamics from ES-FSRS measurements using a 4 mW versus 2.5 mW Raman pump. The actinic pump and Raman pump wavelengths for the ES-FSRS measurements were 490 and 540 nm, respectively, with Raman probe on the Stokes side. The ~1400 and 865 cm^{-1} excited-state Raman modes are compared between LEA **(A** and **C)** and LEA-A69T **(B** and **D)**. The unnormalized spectral data points are shown as hollow circles with the least-squares exponential fits overlaid as solid curves. The color-coded time constants and amplitude weights (in percentages) are listed accordingly in the panel insets.

The intensity dynamics of the ~1400 and 865 cm^{-1} ES-FSRS modes are displayed for LEA (Figures S12A,C) and LEA-A69T (Figures S12B,D) with relatively low and high Raman pump (R_{pu}) powers. In each case, the peaks collected with the higher R_{pu} powers are more intense upon photoexcitation, although the peak intensities are reduced by ~10–20% around time zero with the lower R_{pu} power, different from the expected ~38% reduction due to the R_{pu} power drop if a linear relationship applied (i.e., (4–2.5)/4 ≈ 38%). This finding exemplifies the nonlinear dependence of FSRS peak intensities on the pump powers during the measurements (Dietze and Mathies, 2016; Fang et al., 2019; Fang and Tang, 2020). Furthermore, there is a clear dependence of the sub-ps decay (100–150 fs time constant) on the R_{pu} power. For example, the weighted amplitude of the sub-ps component is reduced by ~4–10% with the lower R_{pu} power than the 4 mW R_{pu} case. Since the specific R_{pu} wavelength was chosen to be resonant with the vibronically-coupled shoulder at the SE band red edge from fs-TA experiments on LEA and LEA-A69T in this work (Figure 7), a higher R_{pu} power may force the first singlet excited state population (S_1) back to the electronic ground state (S_0) to a larger degree than the experiments with a reduced R_{pu} power. This point is evidenced by the larger sub-ps intensity decay of transient ES-

FSRS peaks, which represents the S_1 population moving out of the Franck-Condon region. Such a phenomenon can be termed a "pump-pump-dump" experiment (Redeckas et al., 2016; Fang et al., 2019; Wang et al., 2023). In particular, the ~1400 cm^{-1} mode in LEA shows the largest change of the sub-ps decay magnitude between 4 mW (86%) to 2.5 mW (76%) (see Figure S12A) when compared to the other cases (Figure S12B–D). This observation indicates that the vibronically-coupled mode is especially strong near the Franck-Condon region (Kumpulainen et al., 2017; Fang et al., 2019) and prone to "dumping" interactions with the high-power R_{pu} (see Section 3.3 in main text).

Interestingly, after the initial sub-ps decay, the stimulated Raman peak intensities remain consistently lower or similar for the LEA-A69T peaks (Figure S12B,D) with the lower R_{pu} power (after ~300–400 fs), whereas the peak intensities are notably reduced after the initial sub-ps decay from the experiments performed on LEA with the higher R_{pu} power (Figure S12A,C). This observation likely stems from the resonance conditions: due to the redshifted SE feature of LEA relative to LEA-A69T (Figure 7), the 540 nm R_{pu} is more on resonance with the SE feature and results in a larger degree of "dumping" of the excited-state population, in accord with the "inflated" initial decay component with 82–86% weighted amplitudes. Furthermore, the LEA ES-FSRS spectral comparisons reveal that the intermediate decay component (73–87 ps) becomes more pronounced with the lower R_{pu} power, providing a more robust assessment of the chromophore ring-twisting component in the excited state (Krueger et al., 2023b). Note that the Raman mode intensity dynamic plots here and in Figure 10 were fitted multiple times to examine the effects of inclusion of the intermediate time constant (tens of ps) on the least-squares residual so the sometimes small yet non-negligible temporal component can be systematically compared between various excited-state Raman modes, and between various relevant FP samples, to gain structural dynamics insights.

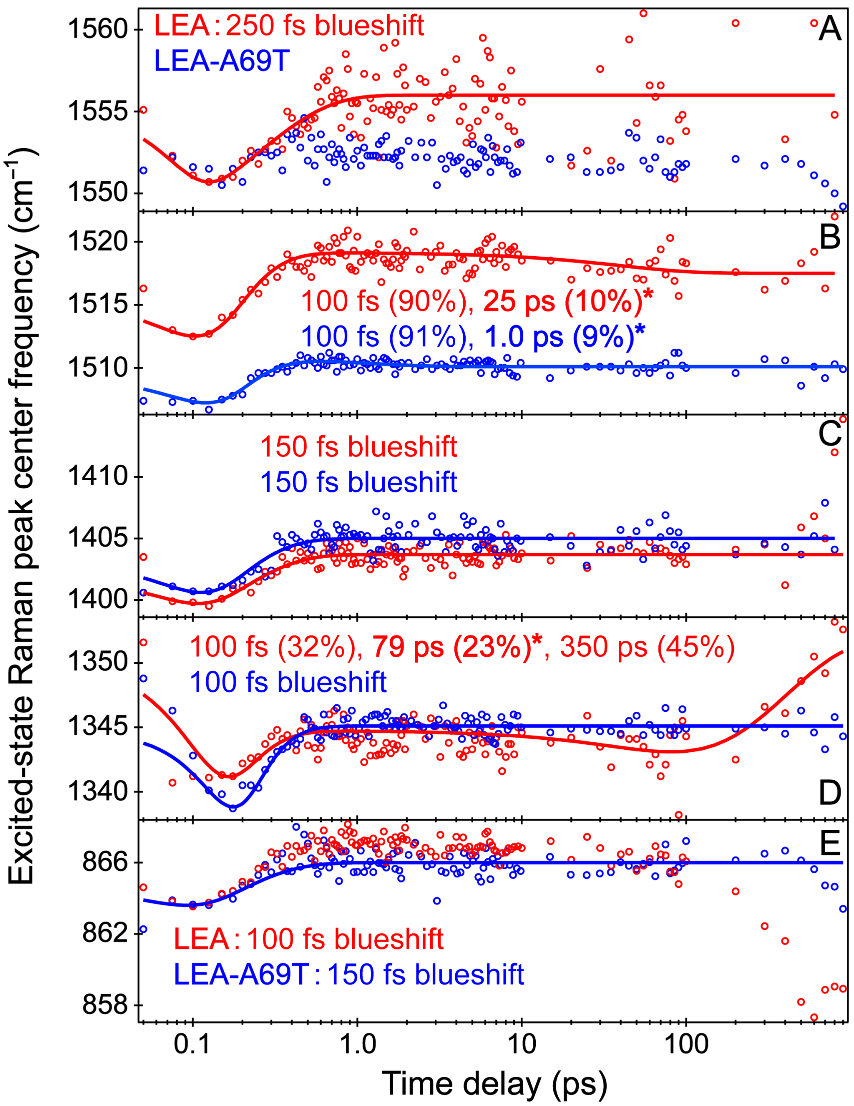

Figure S13. Comparison of the excited-state Raman mode frequency dynamics from ES-FSRS data on LEA (red) and LEA-A69T (blue). The ES-FSRS spectra were collected with actinic and Raman pump wavelengths of 490 and 540 nm, respectively, with a ~2.5 mW Raman pump. The **(A)** ~1550, **(B)** 1510, **(C)** 1400, **(D)** 1340, and **(E)** 865 cm^{-1} modes are compared. The data points (hollow circles) represent the least-squares gaussian-fit center frequencies of the respective peaks, and the exponential fits are shown as solid curves when applicable. The exponential time constants and amplitude weights (in percentages) are listed in the insets. The bolded asterisk denotes a frequency redshift.

Notably, the ES-FSRS frequency dynamics of LEA and LEA-A69T (Figure S13) can represent transient molecular processes such as vibrational cooling (Kumpulainen et al., 2017; Liu et al., 2017) that typically results in a frequency blueshift due to the anharmonicity of the potential energy surface (PES), conformational motions and/or electron redistribution, both of which can result in frequency blueshifts or redshifts depending on the nature of the motions or redistribution (Dietze and Mathies, 2016; Fang et al., 2019). The majority of the frequency shifts occur on the sub-ps timescale (100–250 fs): these blueshifts are indicative of the S_1 population rapidly exiting the Franck-Condon region after photoexcitation and sliding down the anharmonic PES. Moreover, the timescale of this sub-ps blueshift matches the prominent intensity decay of the ES-FSRS peaks for LEA and LEA-A69T (see Figure S12 above and Figure 10 in main text).

Several of the frequency shifts between LEA and LEA-A69T are similar, which include the ~1400 and 865 cm^{-1} modes (Figures S13C,E), although the latter peak shows a pronounced frequency redshift after ~100 ps in LEA that is greatly reduced in LEA-A69T. For comparison, the ~1550 cm^{-1} mode shows a pronounced frequency blueshift in LEA (250 fs time constant) which is not present in LEA-A69T (Figure S13A). Similarly, though the ~1340 cm^{-1} mode features a similar 100 fs blueshift in both pcFPs, the LEA frequency dynamics exhibit an additional redshift and blueshift on the ~80 and 350 ps timescales, respectively (Figure S13D), consistent with a more flexible chromophore that readily undergoes the *on→off* photoswitching in LEA versus LEA-A69T (Figure 1E). We note that this specific mode shows a delayed intensity rise which peaks around 1 ps in LEA-A69T (see Figure 10C in main text); therefore, the lack of further frequency shift following the delayed rise may be indicative of a GB*' state that could be a trapped or semi-trapped green chromophore species during the photoswitching events of a pcFP (Bourgeois and Adam, 2012; Nienhaus and Nienhaus, 2014; Krueger et al., 2020; Krueger et al., 2023a).

A majority of the ES-FSRS peak frequencies are similar between LEA and LEA-A69T, confirming the similar chromophore structure and local environment in the protein matrix. However, the ~1550 and 1510 cm^{-1} peaks noticeably differ with the LEA-A69T peaks being consistently redder than the LEA counterparts (Figure S13A,B). Given that these peaks are largely composed of C=N and C=O stretching modes of the chromophore I-ring (Tables S2 and S3), the observed frequency differences likely originate from the altered interactions between Arg66 and the I-ring in these two pcFPs (see Figure 5 in main text and Figure S9 above).

2. Supplementary Tables

Table S1. The ground-state (GS) and excited-state (ES) peak frequencies from FSRS measurements on the Stokes side of LEA, LEA-H62X, LEA-A69T, and ALL-Q62H in pH=7.9 buffer solution

LEA (GS[a]/ES[b] peak frequencies, cm^{-1})	LEA-H62X (GS peak frequencies, cm^{-1})	LEA-A69T (GS/ES peak frequencies, cm^{-1})	ALL-Q62H (GS/ES peak frequencies, cm^{-1})
1851/1864[c]	1854[c]	1875/1890[c]	1868/1875[c]
1630/1647	1629	1625/1652	1624/1648
1570/1582	1573	1571/1586	1569/1588
1538/1547	1542	1540/1555	1538/1545
1501/1511	1501	1500/1510	1498/1508
1465/1469	1460	1456/1459	1450/1459
1374/1397	1376	1374/1393	1373/1385
1332/1341	1334	1336/1342	1327/1336
1282/1296	1286	1281/1293	1282/1290
1263/1267	1264	1262/1270	1261/1268
1172/1183	1171	1169/1181	1167/1177
1084/1092	1086	1087/1100	1083/1094
927/937	927	929/940	925/937
848/862	849	850/866	848/863
790/802	793	789/801	786/799
767/777	770, 753	771/775	768/780
709/727	709	713/727	710/724
674/683	668	666/677	678/682
645/656	643	649/643	648/656
618/626	621	621/626	619/627
565/575	570	566/583	572/578

[a] The GS-FSRS peak frequencies were measured with a 540 nm Raman pump (~4 mW average power). See Figure 9 for the experimental spectra of LEA, LEA-A69T, and ALL-Q62H, and the least-squares gaussian peak fits were performed to retrieve the center peak frequencies. In addition, we took the GS-FSRS measurement of LEA-H62X at a lower sample volume (using a stir bar instead of flow cell) with a lower power (~2 mW) 540 nm Raman pump (see Figure 3, and the Materials and Methods Section 2.3), and the gaussian fit values are listed in the second column. Moreover, the GS-FSRS peak frequencies for LEA, LEA-A69T, and ALL-Q62H have also been reported with a 510–520 nm Raman pump (Krueger et al., 2020; Krueger et al., 2023b) that achieves a better resonance enhancement with the ground-state absorption peaks of these FPs, while some excited-state contributions could mix in (Quick et al., 2015; Chen et al., 2018; Fang et al., 2019), hence some slight variations are expected.

[b] The ES-FSRS peak frequencies were measured with a 490 nm actinic pump and a 540 nm Raman pump (~4 mW average power). Peak frequencies were determined near the time zero of photoexcitation when the peaks were at maximum intensity, as shown by the horizontal dashed lines in Figure 8. We note that the absence of ES-FSRS results of LEA-H62X was due to current experimental limitations (see Materials and Methods section in main text, particularly Sections 2.1.2 and 2.3).

[c] This high-frequency Raman peak is atypical for a photoexcited protein chromophore (Laptenok et al., 2018; Fang and Tang, 2020) which likely involves some adjacent residues and/or a combination band.

Table S2. The experimental GS-FSRS and calculated Raman peak frequencies of LEA from the optimized and unoptimized chromophore structures

Peak freq. (cm^{-1})[a]	Calc. freq. (cm^{-1})[b]	Calc. freq. (cm^{-1})[c]	Mode assignment[d]
1851	—	—	—
1630	1631	1625	bridge C=C str., P-ring C=C str., I-ring C=O str.
1570	1587	1573	I-ring C=N str., P-ring C=O str., bridge C=C str.
1538	1552	1525	I-ring C=N and C=O str., bridge C=C str., P-ring C=C/C=O str.
1501	1509	1495	P-ring C=C str., I-ring C=N str.
1465	1447	1432	His62 C=N str., imid. bridge-H bending
1374	1375	1368	imid. bridge H-rocking and I-ring i.p. deformation
1332	1327	1321	bridge H-rocking, P-ring H-rocking, I-ring i.p. deformation
1282	1300	1279	P-ring CC str. and I-ring C–N str., collective H-rocking across rings
1263	1271	1248	P-ring H-bending, bridge CC str., I-ring i.p. deformation, imid. bridge H-motions
1172	1165	1128[e]	P-ring H-scissoring, bridge H-rocking
1084	1083	1079	P-ring H-scissoring, imid. bridge CC str.
927	964	931	His62 i.p. deformation, imid. bridge bending motions
848	858	861	P-ring breathing, bridge CCC bending, I-ring i.p. deformation
790	788	789	P-ring HOOP and breathing
767	747	753	I-ring OOP deformation, imid. bridge bending, P-ring HOOP
709	721	713	imid. bridge motions and CC bending (global)
674	690	668	His62 HOOP
645	638	640	His62 HOOP, imid. bridge motions, I-ring OOP deformation
618	625	619	P-ring i.p. deformation, I-ring deformation
565	572	544	P-ring and P-to-I-ring bridge HOOP, I-ring OOP deformation

[a] The GS-FSRS peak frequencies were measured with a 540 nm Raman pump (~4 mW average power).

[b] The calculated Raman peak frequencies for the optimized chromophore structure are scaled by 0.98.

[c] The calculated Raman peak frequencies for the unoptimized chromophore structure are unscaled.

[d] The abbreviations for key vibrational motions are: phenolate ring (P-ring), imidazolinone ring (I-ring), histidine ring (His62), P-to-I-ring bridge (bridge), I-ring-to-His62 bridge (imid. bridge), stretching (str.), hydrogen rocking (H-rocking), in-plane (i.p.), hydrogen motions (H-motions), hydrogen scissoring (H-scissoring), hydrogen-out-of-plane (HOOP), out-of-plane (OOP).

[e] This significantly stronger mode in the unoptimized chromophore structure directly calculated from the LEA crystal structure (Kim et al., 2013) indicates the high sensitivity of P-ring H-scissoring with the nearby bridge H-rocking motions to dihedral angles between P- and I-rings, as well as the intrinsic electron density distribution across the chromophore ring systems (Martin et al., 2004; Altoè et al., 2005; Taylor et al., 2019; Fang and Tang, 2020). This mode typically displays a frequency blueshift upon photoexcitation of the chromophore, demonstrated for several GFPs and GFP-based biosensors (Oscar et al., 2014; Fang and Tang, 2020) as well as Figure 9 (see main text), likely in correlation with the light-induced intramolecular charge transfer from P- to I-ring and the associated conformational motions on molecular timescales (Kumpulainen et al., 2017; Lin et al., 2019; Chen and Fang, 2020; Jones et al., 2021). Using the unoptimized chromophore structure from LEA crystal structure, this mode also consists of I-ring deformation that primarily involves the CCN bending.

Table S3. The experimental GS-FSRS and calculated Raman peak frequencies of LEA-A69T from the optimized and unoptimized chromophore structures

Peak freq. (cm^{-1})[a]	Calc. freq. (cm^{-1})[b]	Calc. freq. (cm^{-1})[c]	Mode assignment[d]
1875	—	—	—
1625	1631	1644	bridge C=C str., P-ring C=C str., I-ring C=O str.
1571	1587	1601	I-ring C=N str., P-ring C=O str., bridge C=C str.
1540	1552	1557	I-ring C=N and C=O str., bridge C=C str., P-ring C=C/C=O str.
1500	1509	1501	P-ring C=C str., I-ring C=N str.
1456	1447	1471	His62 C=N str., imid. bridge-H bending
1374	1375	1368	imid. bridge H-rocking and I-ring i.p. deformation
1336	1327	1355	bridge H-rocking, P-ring H-rocking, I-ring i.p. deformation
1281	1300	1273	P-ring CC str. and I-ring C–N str., collective H-rocking across rings
1262	1271	1255	P-ring H-bending, bridge CC str., I-ring i.p. deformation, imid. bridge H-motions
1169	1165	1168	P-ring H-scissoring, bridge H-rocking
1087	1083	1069	P-ring H-scissoring, imid. bridge CC str.
929	964	945	His62 i.p. deformation, imid. bridge bending motions
850	858	864	P-ring breathing, bridge CCC bending, I-ring i.p. deformation
789	788	808	P-ring HOOP and breathing
771	747	767	I-ring OOP deformation, imid. bridge bending, P-ring HOOP
713	721	705	imid. bridge motions and CC bending (global)
666	690	667	His62 HOOP
649	638	629	His62 HOOP, imid. bridge motions, I-ring OOP deformation
621	625	615	P-ring i.p. deformation, I-ring deformation
566	572	539	P-ring and P-to-I-ring bridge HOOP, I-ring OOP deformation

[a] The GS-FSRS peak frequencies were measured with a 540 nm Raman pump (~4 mW average power). Due to the redder Raman pump at 540 nm used in this work (for a direct comparison with the other FPs in Figure 3B) than a previous report using a Raman pump at ~510–520 nm (Krueger et al., 2023b), the GS-FSRS peak intensities notably decrease herein due to the worsened resonance conditions with respect to the ground-state electronic absorption peak of the LEA-A69T green chromophore (GB) at ~496 nm (see Figure 1D in main text).

[b] The calculated Raman peak frequencies for the optimized anionic chromophore structure are scaled by 0.98. Due to the A69T mutation near the chromophore, the current calculations focusing on the HYG chromophore itself gave the same results as LEA in Table S2 above.

[c] The calculated Raman peak frequencies for the unoptimized chromophore structure are unscaled.

[d] The abbreviations for key vibrational motions are: phenolate ring (P-ring), imidazolinone ring (I-ring), histidine ring (His62), P-to-I-ring bridge (bridge), I-ring-to-His62 bridge (imid. bridge), stretching (str.), hydrogen rocking (H-rocking), in-plane (i.p.), hydrogen motions (H-motions), hydrogen scissoring (H-scissoring), hydrogen-out-of-plane (HOOP), out-of-plane (OOP) (Krueger et al., 2020; Chen et al., 2023a).

Table S4. The experimental GS-FSRS and calculated Raman peak frequencies of ALL-Q62H from the optimized chromophore structures

Peak freq. (cm^{-1})[a]	Calc. freq. (cm^{-1})[b]	Mode assignment[c]
1868	—	—
1624	1631	bridge C=C str., P-ring C=C str., I-ring C=O str.
1569	1587	I-ring C=N str., P-ring C=O str., bridge C=C str.
1538	1552	I-ring C=N and C=O str., bridge C=C str., P-ring C=C and C=O str.
1498	1509	P-ring C=C str., I-ring C=N str.
1450	1447	His62 C=N str., imid. bridge-H bending
1373	1375	imid. bridge H-rocking and I-ring i.p. deformation
1327	1327	bridge H-rocking, P-ring H-rocking, I-ring i.p. deformation
1282	1300	P-ring CC str. and I-ring C–N str., collective H-rocking across rings
1261	1271	P-ring H-bending, bridge CC str., I-ring i.p. deformation, imid. bridge H-motions
1167	1165	P-ring H-scissoring, bridge H-rocking
1083	1083	P-ring H-scissoring, imid. bridge CC str.
925	964	His62 i.p. deformation, imid. bridge bending motions
848	858	P-ring breathing, bridge CCC bending, I-ring i.p. deformation
786	788	P-ring HOOP and breathing
768	747	I-ring OOP deformation, imid. bridge bending, P-ring HOOP
710	721	imid. bridge motions and CC bending (global)
678	690	His62 HOOP
648	638	His62 HOOP, imid. bridge motions, I-ring OOP deformation
619	625	P-ring i.p. deformation, I-ring deformation
572	572	P-ring and P-to-I-ring bridge HOOP, I-ring OOP deformation

[a] The GS-FSRS peak frequencies were measured with a 540 nm Raman pump (~4 mW average power).

[b] The calculated Raman peak frequencies for the optimized chromophore structure are scaled by 0.98. The calculations on the HYG chromophore after geometrical optimization should be identical to the aforementioned LEA calculation results in Table S2 (listed in the second column therein).

[c] The abbreviations for key vibrational motions are: phenolate ring (P-ring), imidazolinone ring (I-ring), histidine ring (His62), P-to-I-ring bridge (bridge), I-ring-to-His62 bridge (imid. bridge), stretching (str.), hydrogen rocking (H-rocking), in-plane (i.p.), hydrogen motions (H-motions), hydrogen scissoring (H-scissoring), hydrogen-out-of-plane (HOOP), out-of-plane (OOP).

Table S5. The experimental GS-FSRS and calculated Raman peak frequencies of LEA-H62X from the optimized and unoptimized chromophore structures

Peak freq. (cm^{-1})[a]	Calc. freq. (cm^{-1})[b]	Calc. freq. (cm^{-1})[c]	Mode assignment[d]
1854	—	1800	I-ring C=O str.
1629	1635	1655	bridge C=C str., P-ring C=C str., I-ring C=O str.
1573	1584	1611	I-ring C=N str., P-ring C=O str., bridge C=C str.
1542	1553	1541	I-ring C=N and C=O str., bridge C=C str., P-ring C=C/C=O str.
1501	1509	1508	P-ring C=C str., I-ring C=N str.
1460	1447	1453	His62 C=N str., imid. bridge-H bending
1376	1366	1383	imid. bridge H-rocking and I-ring i.p. deformation
1334	1326	1355	bridge H-rocking, P-ring H-rocking, I-ring i.p. deformation
1286	1295	1277	P-ring CC str. and I-ring C–N str., collective H-rocking across rings
1264	1269	1255	P-ring H-bending, bridge CC str., I-ring i.p. deformation, imid. bridge H-motions
1171	1153	1177	P-ring H-scissoring, bridge H-rocking
1086	1073	1075	P-ring H-scissoring, imid. bridge CC str.
927	964	949	His62 i.p. deformation, imid. bridge bending motions
849	841	853	P-ring breathing, bridge CCC bending, I-ring i.p. deformation
793	806	796	P-ring HOOP and breathing
770, 753[e]	786	768, 753[e]	I-ring OOP deformation, imid. bridge bending, P-ring HOOP
709	707	685	imid. bridge motions and CC bending (global), His62X HOOP
668	675[f]	668[f]	His62X CNC bending with the methyl group, His62X HOOP[e]
643	627	636	His62X HOOP, imid. bridge motions, I-ring OOP deformation
621	605	598	P-ring i.p. deformation, I-ring deformation
570	577	564	P-ring and P-to-I-ring bridge HOOP, I-ring OOP deformation

[a] The GS-FSRS peak frequencies were measured with a 540 nm Raman pump (~2 mW average power).

[b] The calculated Raman peak frequencies for the optimized chromophore structure are scaled by 0.98. Due to the extra methyl group on the His62 ring in LEA-H62X, the calculated values listed in this column allow for a detailed comparison to the counterpart of HYG chromophore in LEA (see the second column in Table S2).

[c] The calculated Raman peak frequencies for the unoptimized chromophore structure are unscaled.

[d] The abbreviations for key vibrational motions are: phenolate ring (P-ring), imidazolinone ring (I-ring), histidine ring (His62), P-to-I-ring bridge (bridge), I-ring-to-His62 bridge (imid. bridge), stretching (str.), hydrogen rocking (H-rocking), in-plane (i.p.), hydrogen motions (H-motions), hydrogen scissoring (H-scissoring), hydrogen-out-of-plane (HOOP), out-of-plane (OOP).

[e] The 753 cm^{-1} mode that is shifted and more intense in LEA-H62X (compared to LEA, see Figure 3A inset) can be assigned to the rather unique His62X ring breathing that induces H-motions on the extra methyl group, and some I-ring deformation. A nearby mode (770 cm^{-1}) also involves the P-ring and P-to-I-ring bridge HOOP motions, similar to the 767 cm^{-1} mode in LEA.

[f] These calculation results support the key assignment of the experimentally observed ~668 cm^{-1} mode in LEA-H62X which gets stronger and redshifts from the 670 cm^{-1} mode in LEA (see Figure 3A), owing to the CNC bending motion due to the extra methyl group on His62 that mixes with the original His62 HOOP motions (e.g., 690 cm^{-1} calculated for the optimized HYG chromophore in Table S2). See Figure S4 above for the unoptimized chromophore structures.

Table S6. Crystallographic statistics for the LEA-A69T and LEA-H62X structures

Structure	LEA-A69T	LEA-H62X, X = 3mH
**Data collection**		
Space group[¶]	$P2_12_12_1$	$I222$
Cell dimensions<br>*a*, *b*, *c* (Å)<br>α, β, γ (deg)	 72.447, 106.084, 123.471<br>90, 90, 90	 46.778, 76.733, 120.070<br>90, 90, 90
Total Reflections	1,183,642 (33,660)*	209,736 (10,899)
Unique Reflections	152,026 (7,274)	24,128 (1,279)
Resolution (Å)[†]	48.74 – 1.50 (1.53 – 1.50)	43.59 – 1.70 (1.73 – 1.70)
$I/\sigma(I)$	18.3 (0.8)	10.7 (0.2)
R_{meas}	0.071 (2.050)	0.060 (4.242)
R_{pim}	0.025 (0.927)	0.020 (1.425)
$CC_{1/2}$	1.000 (0.331)	1.000 (0.367)
R_{merge}	0.066 (1.814)	0.056 (3.987)
Completeness (%)	99.8 (97.2)	99.7 (99.8)
**Refinement**		
PDB entry	8THS	8UB6
Resolution (Å)[†]	47.03 – 1.50	39.97 – 1.70
R_{work}	0.1623	0.2094
R_{free}	0.1881	0.2398
Number of protein atoms	7,146	1,698
Number of solvent atoms	1,010	82
Average *B* factor (Å^2)	24.73	48.25
Protein atoms (Å^2)	23.32	48.41
Solvent atoms (Å^2)	34.10	44.66
rmsd bond lengths (Å)	0.0130	0.0081
rmsd bond angles (deg)	1.7685	1.4971

[¶]For comparison, the space groups for the crystallized low-pH and high-pH LEA are $P2_12_12_1$ and $I222$, respectively, with four molecules and one molecule per asymmetric unit (Kim et al., 2013).

[*]The values in parentheses indicate statistics for the highest resolution shell.

[†]The resolution numbers outside of the parentheses indicate the range of diffraction data collected. The different large numbers are the top end of the resolution ranges for data collection and those shown below are the ranges used for refinement.

Table S7. Key crystallographic observables for LEA, LEA-A69T, and LEA-H62X

**Observable** [a]	**LEA**	**LEA-H62X**	**LEA-A69T**
P-ring to His193 (Å)	3.8	3.8	3.7
Glu211 to α-carbon (Å)	3.5	3.7	3.5
P-ring dihedral φ (°)	179	172	168
P-ring dihedral τ (°)	180	176	174

[a] All the observables quoted herein for the protein chromophore in its local environment are taken from crystallographic measurements of LEA (PDB ID: 4DXN) (Kim et al., 2013), as well as LEA-H62X (PDB ID: 8UB6) and LEA-A69T (PDB ID: 8THS) in the current work. The corresponding values (from top to bottom rows) can also be found in Figures 5B, S6, and S4, respectively.

3. Supplementary References

Altoè, P., Bernardi, F., Garavelli, M., Orlandi, G., and Negri, F. (2005). Solvent effects on the vibrational activity and photodynamics of the green fluorescent protein chromophore: A quantum-chemical study. *J. Am. Chem. Soc.* 127**,** 3952-3963. doi:10.1021/ja0451517

Bourgeois, D., and Adam, V. (2012). Reversible photoswitching in fluorescent proteins: A mechanistic view. *IUBMB Life* 64**,** 482-491. doi:10.1002/iub.1023

Chen, C., and Fang, C. (2020). Devising efficient red-shifting strategies for bioimaging: A generalizable donor-acceptor fluorophore prototype. *Chem. Asian J.* 15**,** 1514-1523. doi:10.1002/asia.202000175

Chen, C., Henderson, J. N., Ruchkin, D. A., Kirsh, J. M., Baranov, M. S., Bogdanov, A. M., Mills, J. H., Boxer, S. G., and Fang, C. (2023a). Structural characterization of fluorescent proteins using tunable femtosecond stimulated Raman spectroscopy. *Int. J. Mol. Sci.* 24**,** 11991. doi:10.3390/ijms241511991

Chen, C., Zhang, H., Zhang, J., Ai, H.-w., and Fang, C. (2023b). Structural origin and rational development of bright red noncanonical variants of green fluorescent protein. *Phys. Chem. Chem. Phys.* 25**,** 15624-15634. doi:10.1039/D3CP01315D

Chen, C., Zhu, L., and Fang, C. (2018). Femtosecond stimulated Raman line shapes: Dependence on resonance conditions of pump and probe pulses. *Chin. J. Chem. Phys.* 31**,** 492-502. doi:10.1063/1674-0068/31/cjcp1805125

Cui, Q., Pal, T., and Xie, L. (2021). Biomolecular QM/MM simulations: What are some of the "burning issues"? *J. Phys. Chem. B* 125**,** 689-702. doi:10.1021/acs.jpcb.0c09898

Dietze, D. R., and Mathies, R. A. (2016). Femtosecond stimulated Raman spectroscopy. *ChemPhysChem* 17**,** 1224–1251. doi:10.1002/cphc.201600104

Esposito, A. P., Schellenberg, P., Parson, W. W., and Reid, P. J. (2001). Vibrational spectroscopy and mode assignments for an analog of the green fluorescent protein chromophore. *J. Mol. Struct.* 569**,** 25-41. doi:10.1016/S0022-2860(00)00825-5

Fang, C., Frontiera, R. R., Tran, R., and Mathies, R. A. (2009). Mapping GFP structure evolution during proton transfer with femtosecond Raman spectroscopy. *Nature* 462**,** 200-204. doi:10.1038/nature08527

Fang, C., and Tang, L. (2020). Mapping structural dynamics of proteins with femtosecond stimulated Raman spectroscopy. *Annu. Rev. Phys. Chem.* 71**,** 239-265. doi:10.1146/annurev-physchem-071119-040154

Fang, C., Tang, L., and Chen, C. (2019). Unveiling coupled electronic and vibrational motions of chromophores in condensed phases. *J. Chem. Phys.* 151**,** 200901. doi:10.1063/1.5128388

Frisch, M. J., Trucks, G. W., Schlegel, H. B., Scuseria, G. E., Robb, M. A., Cheeseman, J. R., Scalmani, G., Barone, V., Petersson, G. A., Nakatsuji, H., Li, X., Caricato, M., Marenich, A. V., Bloino, J., Janesko, B. G., Gomperts, R., Mennucci, B., Hratchian, H. P., Ortiz, J. V., Izmaylov, A. F., Sonnenberg, J. L., Williams-Young, D., Ding, F., Lipparini, F., Egidi, F., Goings, J., Peng, B., Petrone, A., Henderson, T., Ranasinghe, D., Zakrzewski, V. G., Gao, J., Rega, N., Zheng, G., Liang, W., Hada, M., Ehara, M., Toyota, K., Fukuda, R., Hasegawa, J., Ishida, M., Nakajima, T., Honda, Y., Kitao, O., Nakai, H., Vreven, T., Throssell, K., Montgomery Jr., J. A., Peralta, J. E., Ogliaro, F., Bearpark, M. J., Heyd, J. J., Brothers, E. N., Kudin, K. N., Staroverov, V. N., Keith, T. A., Kobayashi, R., Normand, J., Raghavachari, K., Rendell, A. P., Burant, J. C.,

Iyengar, S. S., Tomasi, J., Cossi, M., Millam, J. M., Klene, M., Adamo, C., Cammi, R., Ochterski, J. W., Martin, R. L., Morokuma, K., Farkas, O., Foresman, J. B., and Fox, D. J. (2016). *Gaussian 16, Revision C.01* (Wallingford, CT: Gaussian, Inc.)

Jones, C. M., List, N. H., and Martínez, T. J. (2021). Resolving the ultrafast dynamics of the anionic green fluorescent protein chromophore in water. *Chem. Sci.* 12**,** 11347-11363. doi:10.1039/D1SC02508B

Kim, H., Grunkemeyer, T. J., Modi, C., Chen, L., Fromme, R., Matz, M. V., and Wachter, R. M. (2013). Acid–base catalysis and crystal structures of a least evolved ancestral GFP-like protein undergoing green-to-red photoconversion. *Biochemistry* 52**,** 8048-8059. doi:10.1021/bi401000e

Kim, H., Zou, T., Modi, C., Dörner, K., Grunkemeyer, Timothy J., Chen, L., Fromme, R., Matz, Mikhail V., Ozkan, S. B., and Wachter, Rebekka M. (2015). A hinge migration mechanism unlocks the evolution of green-to-red photoconversion in GFP-like proteins. *Structure* 23**,** 34-43. doi:10.1016/j.str.2014.11.011

Konold, P. E., Arik, E., Weißenborn, J., Arents, J. C., Hellingwerf, K. J., van Stokkum, I. H. M., Kennis, J. T. M., and Groot, M. L. (2020). Confinement in crystal lattice alters entire photocycle pathway of the photoactive yellow protein. *Nat. Commun.* 11**,** 4248. doi:10.1038/s41467-020-18065-9

Krueger, T. D., Chen, C., and Fang, C. (2023a). Targeting ultrafast spectroscopic insights into red fluorescent proteins. *Chem. Asian J.* 18**,** e202300668. doi:10.1002/asia.202300668

Krueger, T. D., Tang, L., Chen, C., Zhu, L., Breen, I. L., Wachter, R. M., and Fang, C. (2023b). To twist or not to twist: From chromophore structure to dynamics inside engineered photoconvertible and photoswitchable fluorescent proteins. *Protein Sci.* 32**,** e4517. doi:10.1002/pro.4517

Krueger, T. D., Tang, L., Zhu, L., Breen, I. L., Wachter, R. M., and Fang, C. (2020). Dual illumination enhances transformation of an engineered green-to-red photoconvertible fluorescent protein. *Angew. Chem. Int. Ed.* 59**,** 1644-1652. doi:10.1002/anie.201911379

Kumpulainen, T., Lang, B., Rosspeintner, A., and Vauthey, E. (2017). Ultrafast elementary photochemical processes of organic molecules in liquid solution. *Chem. Rev.* 117**,** 10826-10939. doi:10.1021/acs.chemrev.6b00491

Laptenok, S. P., Gil, A. A., Hall, C. R., Lukacs, A., Iuliano, J. N., Jones, G. A., Greetham, G. M., Donaldson, P., Miyawaki, A., Tonge, P. J., and Meech, S. R. (2018). Infrared spectroscopy reveals multi-step multi-timescale photoactivation in the photoconvertible protein archetype Dronpa. *Nat. Chem.* 10**,** 845-852. doi:10.1038/s41557-018-0073-0

Lin, C.-Y., Romei, M. G., Oltrogge, L. M., Mathews, I. I., and Boxer, S. G. (2019). Unified model for photophysical and electro-optical properties of green fluorescent proteins. *J. Am. Chem. Soc.* 141**,** 15250-15265. doi:10.1021/jacs.9b07152

Liu, W., Tang, L., Oscar, B. G., Wang, Y., Chen, C., and Fang, C. (2017). Tracking ultrafast vibrational cooling during excited state proton transfer reaction with anti-Stokes and Stokes femtosecond stimulated Raman spectroscopy. *J. Phys. Chem. Lett.* 8**,** 997–1003. doi:10.1021/acs.jpclett.7b00322

Martin, M. E., Negri, F., and Olivucci, M. (2004). Origin, nature, and fate of the fluorescent state of the green fluorescent protein chromophore at the CASPT2//CASSCF resolution. *J. Am. Chem. Soc.* 126**,** 5452-5464. doi:10.1021/ja037278m

McCamant, D. W., Kukura, P., Yoon, S., and Mathies, R. A. (2004). Femtosecond broadband stimulated Raman spectroscopy: Apparatus and methods. *Rev. Sci. Instrum.* 75**,** 4971-4980. doi:10.1063/1.1807566

Meech, S. R. (2009). Excited state reactions in fluorescent proteins. *Chem. Soc. Rev.* 38**,** 2922-2934. doi:10.1039/B820168B

Mukherjee, S., Manna, P., Hung, S.-T., Vietmeyer, F., Friis, P., Palmer, A. E., and Jimenez, R. (2022). Directed evolution of a bright variant of mCherry: Suppression of nonradiative decay by fluorescence lifetime selections. *J. Phys. Chem. B* 126**,** 4659-4668. doi:10.1021/acs.jpcb.2c01956

Nienhaus, K., and Nienhaus, G. U. (2014). Fluorescent proteins for live-cell imaging with super-resolution. *Chem. Soc. Rev.* 43**,** 1088-1106. doi:10.1039/C3CS60171D

Oscar, B. G., Liu, W., Zhao, Y., Tang, L., Wang, Y., Campbell, R. E., and Fang, C. (2014). Excited-state structural dynamics of a dual-emission calmodulin-green fluorescent protein sensor for calcium ion imaging. *Proc. Natl. Acad. Sci. U.S.A.* 111**,** 10191-10196. doi:10.1073/pnas.1403712111

Quick, M., Dobryakov, A. L., Kovalenko, S. A., and Ernsting, N. P. (2015). Resonance femtosecond-stimulated raman spectroscopy without actinic excitation showing low-frequency vibrational activity in the S_2 state of all-trans *β*-carotene. *J. Phys. Chem. Lett.* 6**,** 1216-1220. doi:10.1021/acs.jpclett.5b00243

Redeckas, K., Voiciuk, V., and Vengris, M. (2016). Investigation of the S_1/ICT equilibrium in fucoxanthin by ultrafast pump–dump–probe and femtosecond stimulated Raman scattering spectroscopy. *Photosynth. Res.* 128**,** 169-181. doi:10.1007/s11120-015-0215-9

Schapiro, I., Gueye, M., Paolino, M., Fusi, S., Marchand, G., Haacke, S., Martin, M. E., Huntress, M., Vysotskiy, V. P., Veryazov, V., Léonard, J., and Olivucci, M. (2019). Synthesis, spectroscopy and QM/MM simulations of a biomimetic ultrafast light-driven molecular motor. *Photochem. Photobiol. Sci.* 18**,** 2259-2269. doi:10.1039/C9PP00223E

Subach, F. V., and Verkhusha, V. V. (2012). Chromophore transformations in red fluorescent proteins. *Chem. Rev.* 112**,** 4308-4327. doi:10.1021/cr2001965

Tang, L., and Fang, C. (2022). Photoswitchable fluorescent proteins: Mechanisms on ultrafast timescales. *Int. J. Mol. Sci.* 23**,** 6459. doi:10.3390/ijms23126459

Taylor, M. A., Zhu, L., Rozanov, N. D., Stout, K. T., Chen, C., and Fang, C. (2019). Delayed vibrational modulation of the solvated GFP chromophore into a conical intersection. *Phys. Chem. Chem. Phys.* 21**,** 9728-9739. doi:10.1039/C9CP01077G

Tozzini, V., Bizzarri, A. R., Pellegrini, V., Nifosi, R., Giannozzi, P., Iuliano, A., Cannistraro, S., and Beltram, F. (2003). The low frequency vibrational modes of green fluorescent proteins. *Chem. Phys.* 287**,** 33-42. doi:10.1016/S0301-0104(02)00976-X

Wang, Z., Zhang, Y., Chen, C., Zhu, R., Jiang, J., Weng, T.-C., Ji, Q., Huang, Y., Fang, C., and Liu, W. (2023). Mapping the complete photocycle that powers a large Stokes shift red fluorescent protein. *Angew. Chem. Int. Ed.* 62**,** e202212209. doi:10.1002/anie.202212209
